# Supplementary material for: Novel Coumarin 7-Carboxamide/Sulfonamide Derivatives as Potential Fungicidal Agents: Design, Synthesis, and Biological Evaluation
Source: Molecules. 2022 Oct 14;27(20):6904. doi: 10.3390/molecules27206904 (PMC9611003; doi:10.3390/molecules27206904)

Supplemental Material For:

Novel Coumarin 7-Carboxamide/Sulfonamide Derivatives as Potential Fungicidal Agents: Design, Synthesis, and Biological Evaluation

Shu-Guang Zhang, Yu-Qiang Wan, Ya Wen and Wei-Hua Zhang *

Jiangsu Key Laboratory of Pesticide Science, College of Sciences, Nanjing Agricultural University, Nanjing 210095, China; shuguangz@njau.edu.cn (S.-G.Z.); 2021111025@stu.njau.edu.cn (Y.-Q.W.); 2016111016@njau.edu.cn (Y.W.)

* Correspondence: zhangweihua@njau.edu.cn; Tel.: +86-025-8439-5255

Table of Contents for Supplemental Material

1. General Procedure for Synthesis of Starting Materials **4**··············· ·············S2

2. Biological Assays ···S2

3. ^{1}HNMR, ^{13}CNMR and HNMR Spectra of Products····················· ···········S4–S60

1. General Procedure for Synthesis of Starting Materials *4*

Preparation of 7-Aminocoumarins (*4*). 7-Aminocoumarins **4** were synthesized through the reported synthetic route.

Benzyl chloroformate (100.0 mmol) and *m*-aminophenol **1** (100.0 mmol) were employed as the starting materials to generate 3-hydroxyphenylurethane **2**. A solution of 3-hydroxyphenylurethylan **2** (2.0 g) and un/substituted ethyl acetoacetate (1.2 eq) suspended in 20.0 mL of 70% ethanolic H_2SO_4 was stirred at room temperature for 4–6 h, and the crude products were crystallized from ethanol to obtain compounds **3** in moderate to good yields. To a 45% KOH (W/W) solution, compounds **3** were added. The mixture was heated at 100 °C for 30 min and then poured into 100 mL of ice water. Concentrated HCl was added to adjust the pH to 1–2, and then the pH was adjusted to 4–5 with ammonia. The resulting solid was dried by suction filtration and purified by flash column chromatography using petroleum ether/ethyl acetate as the eluent to yield the desired products **4**.

Please see: Zhang, S.G.; Liang, C.G.; Sun, Y.Q.; Teng, P.; Wang, J.Q.; Zhang, W.H. Design, synthesis and antifungal activities of novel pyrrole-and pyrazole-substituted coumarin derivatives. *Mol. Divers.* **2019**, *23*, 915-925; Yu, H.N.; Hou, Z.; Tian, Y.; Mou, Y.H.; Guo, C. Design, synthesis, cytotoxicity and mechanism of novel dihydroartemisinin-coumarin hybrids as potential anti-cancer agents. *Eur. J. Med. Chem.* **2018**, *151*, 434-448.

2. Biological Assays

The assay of antifungal activity toward *Botrytis cinerea*, *Alternaria solani*, *Gibberellazeae*, *Rhizoctorzia solani*, *Cucumber anthrax*, and *Alternariamali* was carried out on 100 mm × 15 mm Petri plates, each containing 10 mL of potato dextrose agar, under sterile conditions, on a clean bench in a sterile room. Sterile blank paper disks (0.65 cm in diameter) were placed at a distance 2.5 cm away from the rim of the mycelial

colony. The plates were sealed with parafilm and incubated at 25 ºC until mycelial growth had enveloped the disks containing the control and had formed crescents of inhibition around disks containing samples with antifungal activity. When the mycelia colony of the control had grown to almost fill the plate, the area of the mycelia colony was measured, and the inhibition of fungal growth in the other plates was determined by calculating the percent reduction in the area of the mycelia colony. The resulting data were collated for each compound, and averages across replicates were used to make a judgment of the overall activity level of the compound.

4. ^{1}H NMR, ^{13}C NMR, and HRMS Spectra of Products

^{1}H NMR Spectrum of 5a (400 MHz, DMSO-d_6)

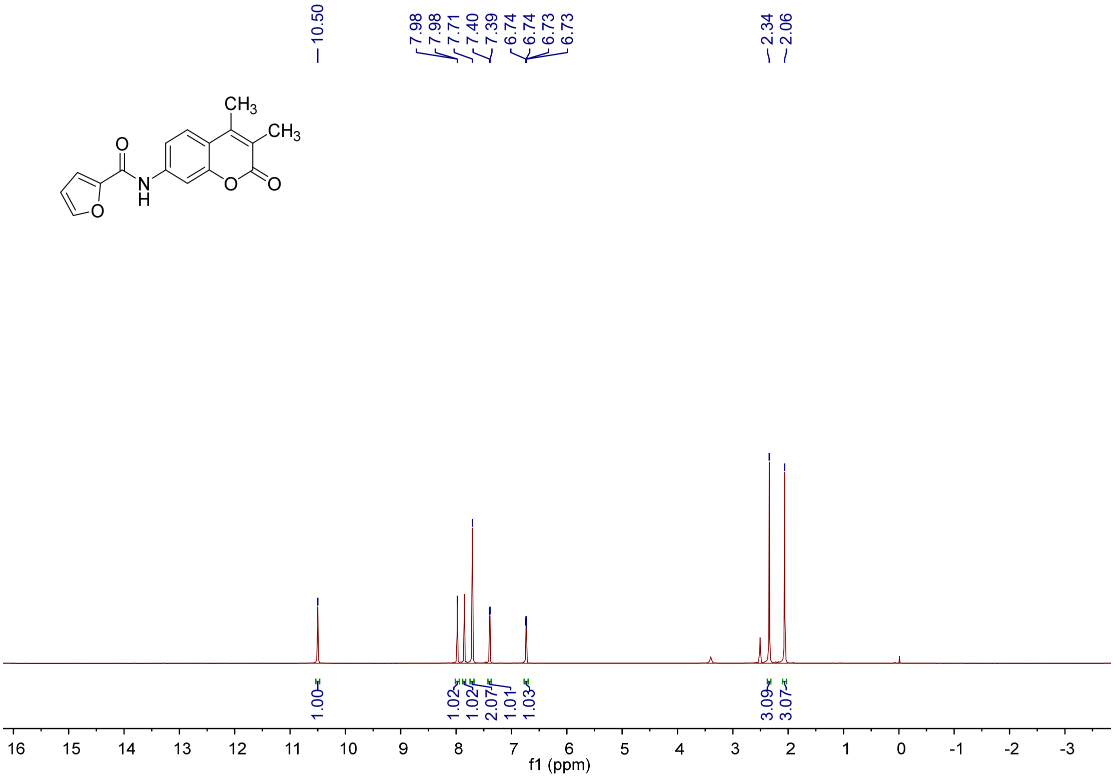

^{13}C NMR Spectrum of 5a (101 MHz, DMSO-d_6)

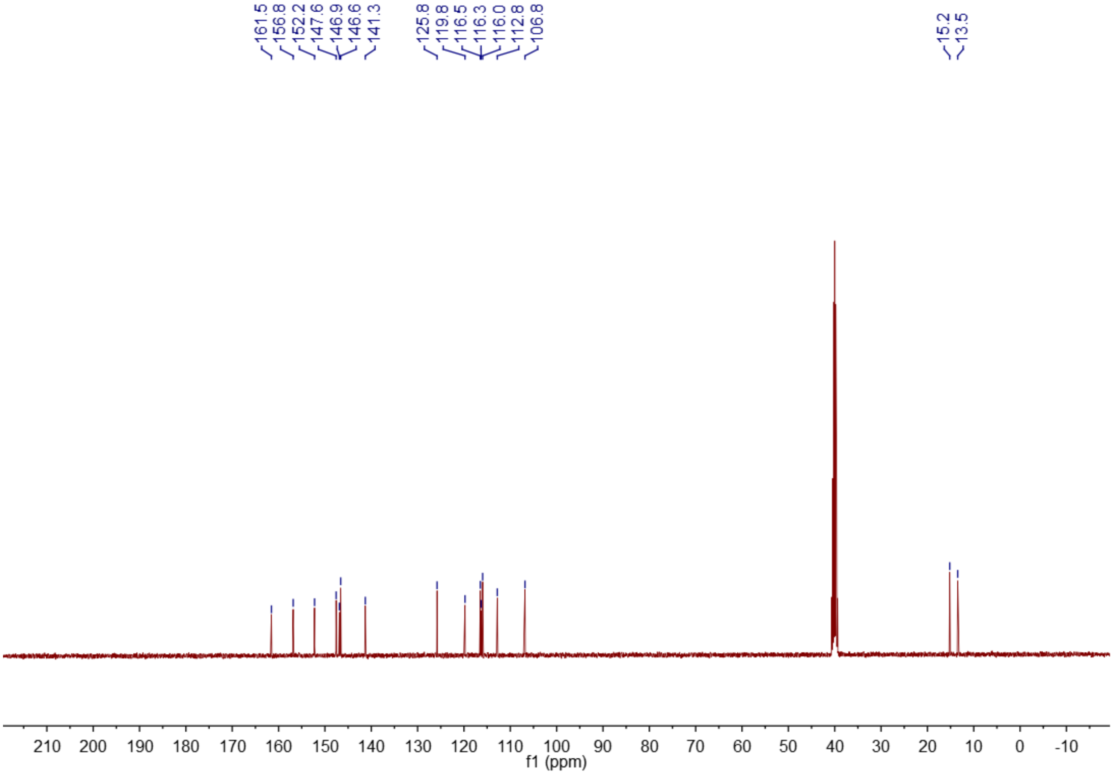

HRMS Spectrum of 5a

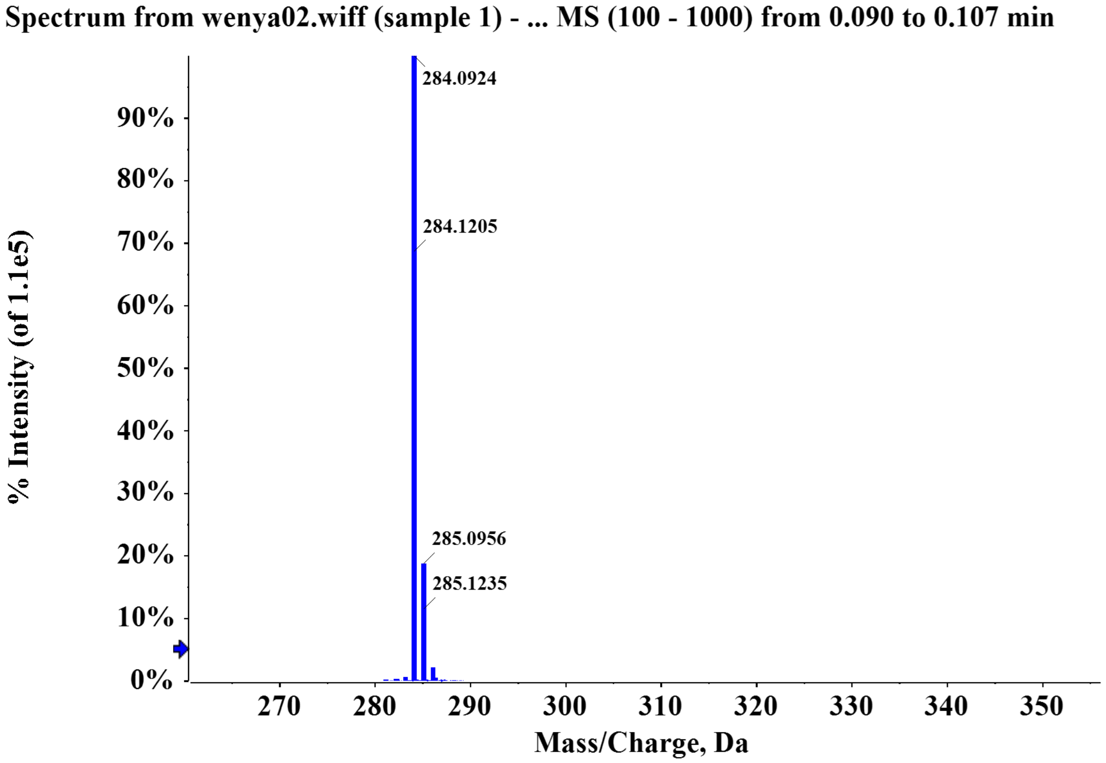

^{1}H NMR Spectrum of 5b (400 MHz, DMSO-d_6)

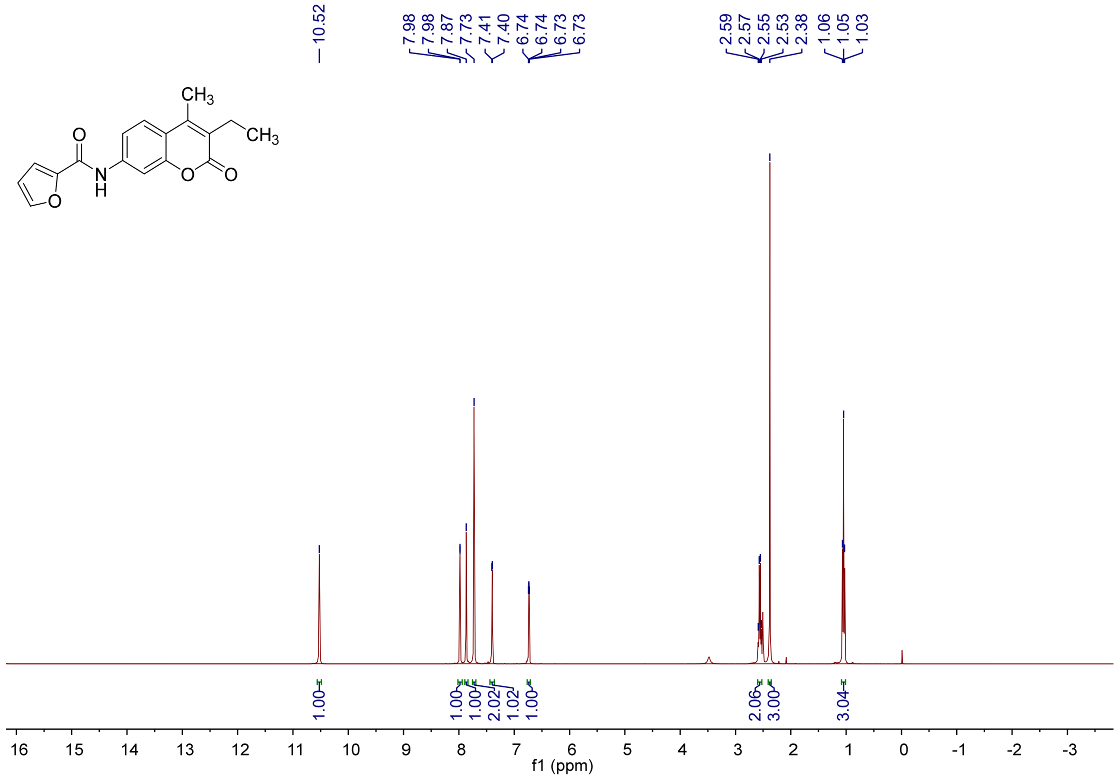

^{13}C NMR Spectrum of 5b (101 MHz, DMSO-d_6)

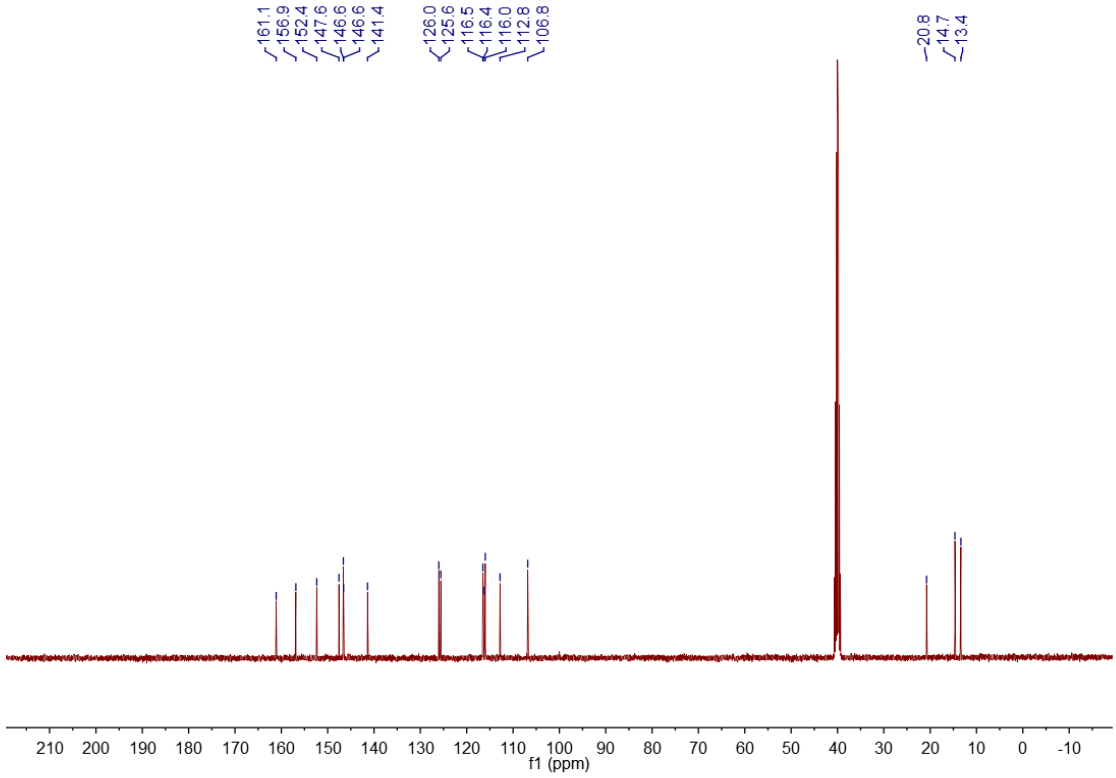

HRMS Spectrum of 5b

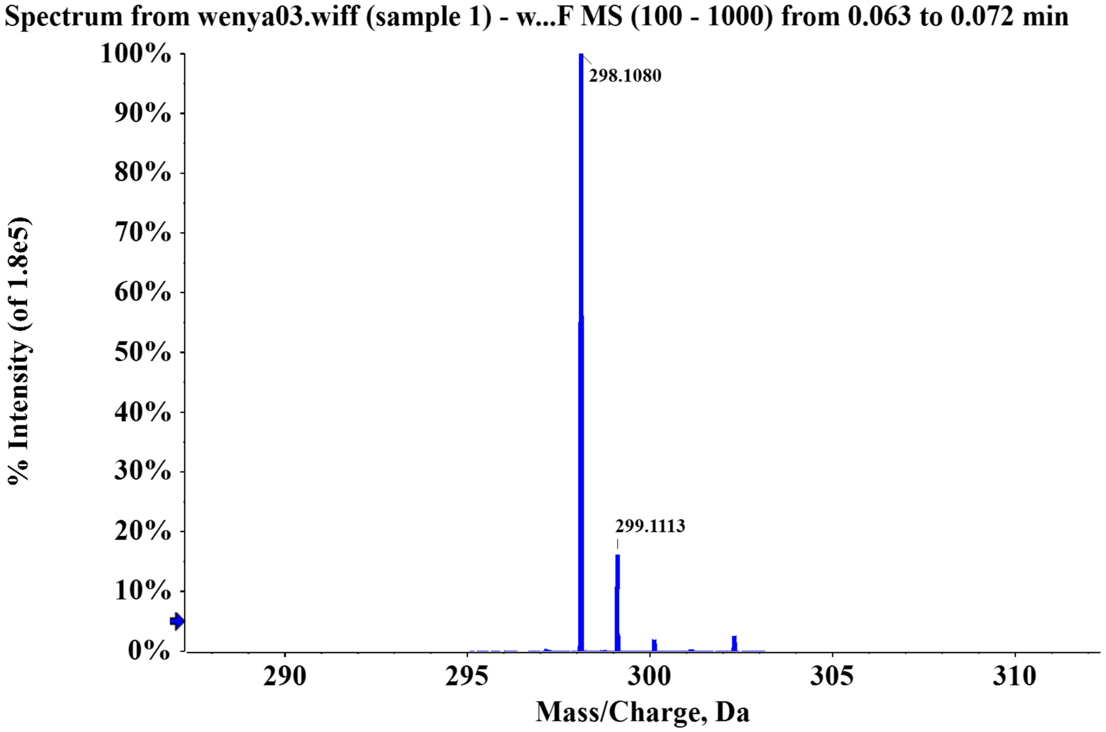

^{1}H NMR Spectrum of 5c (400 MHz, DMSO-d_6)

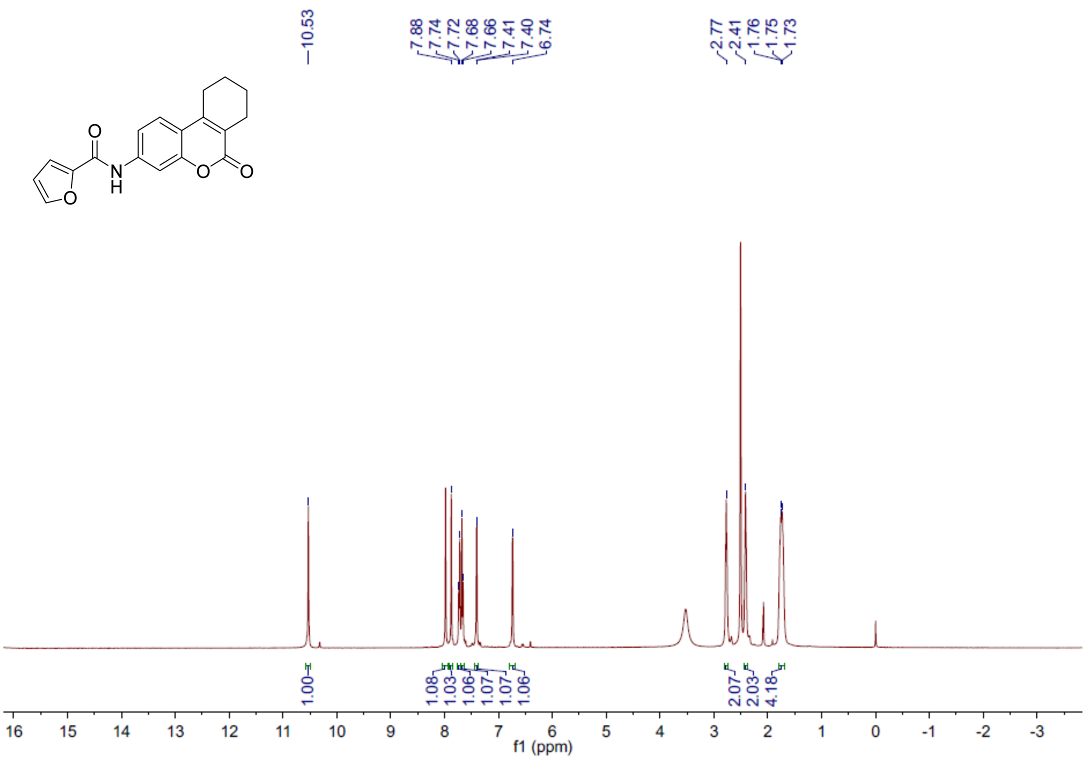

^{13}C NMR Spectrum of 5c (126 MHz, DMSO-d_6)

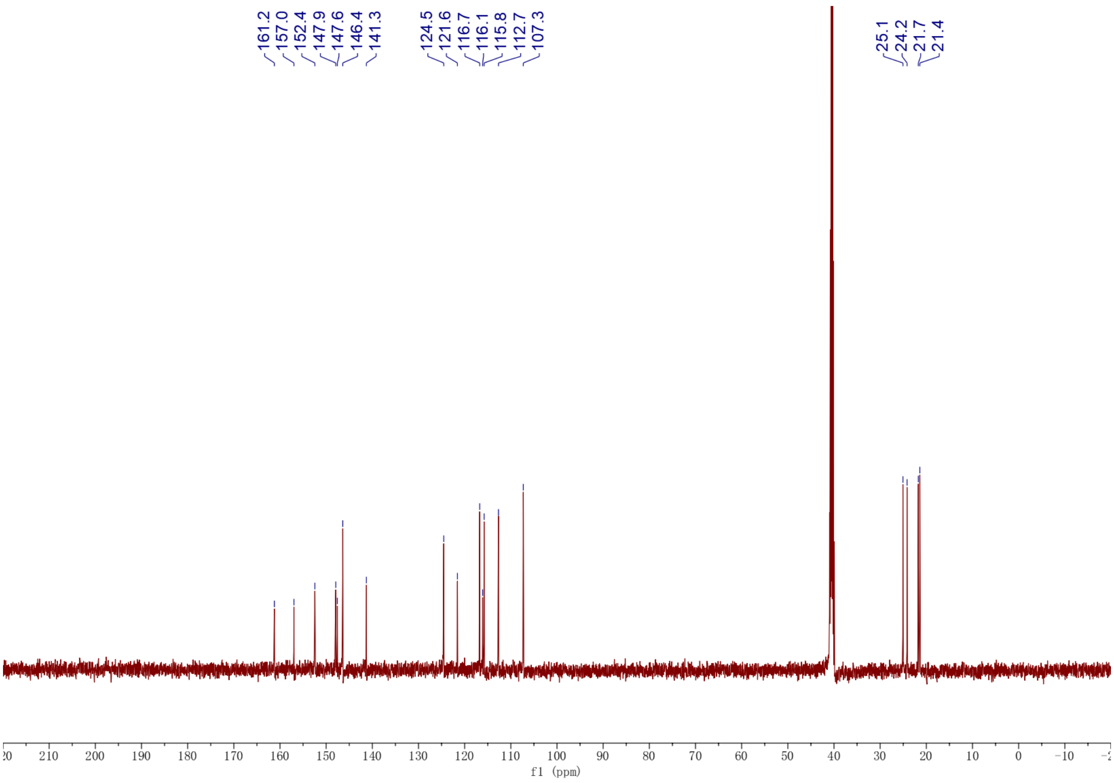

HRMS Spectrum of 5c

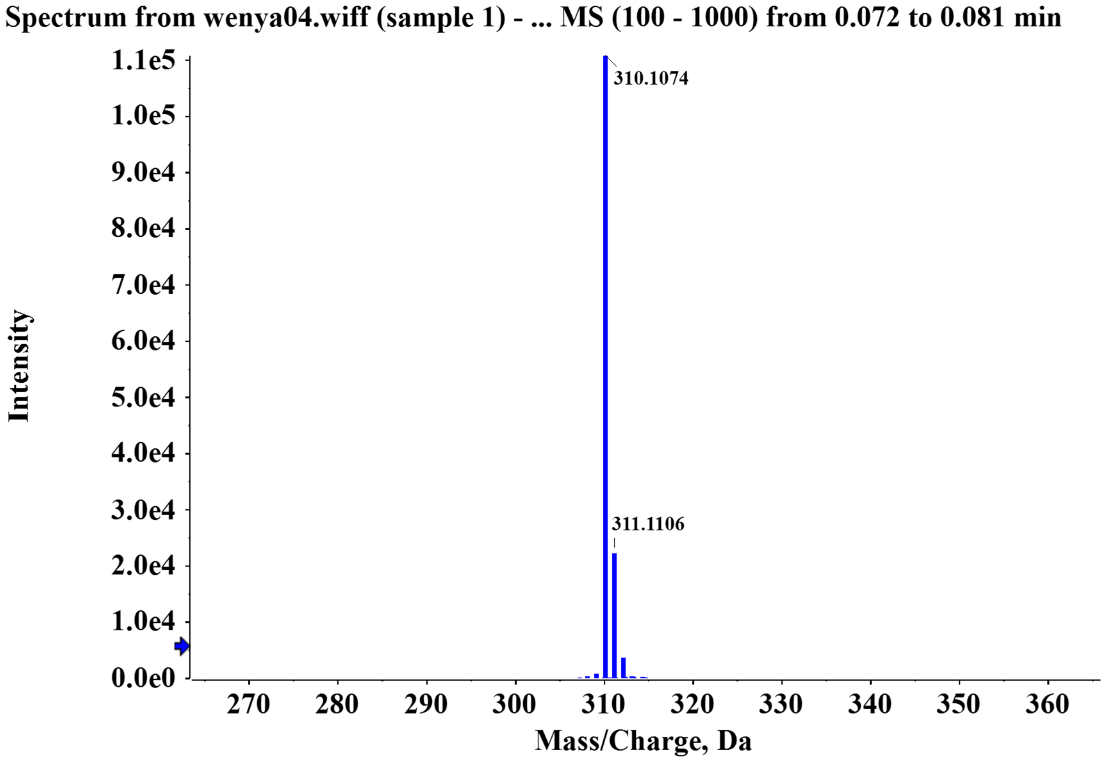

^{1}H NMR Spectrum of 5d (400 MHz, DMSO-d_6)

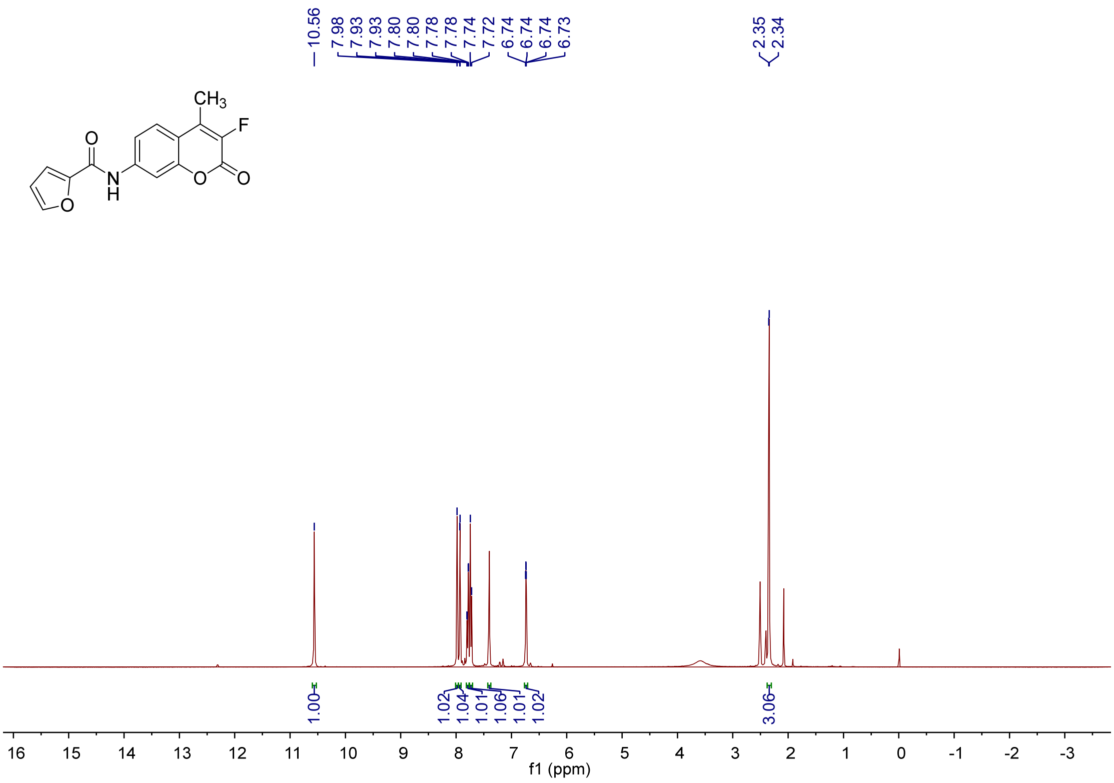

^{13}C NMR Spectrum of 5d (101 MHz, DMSO-d_6)

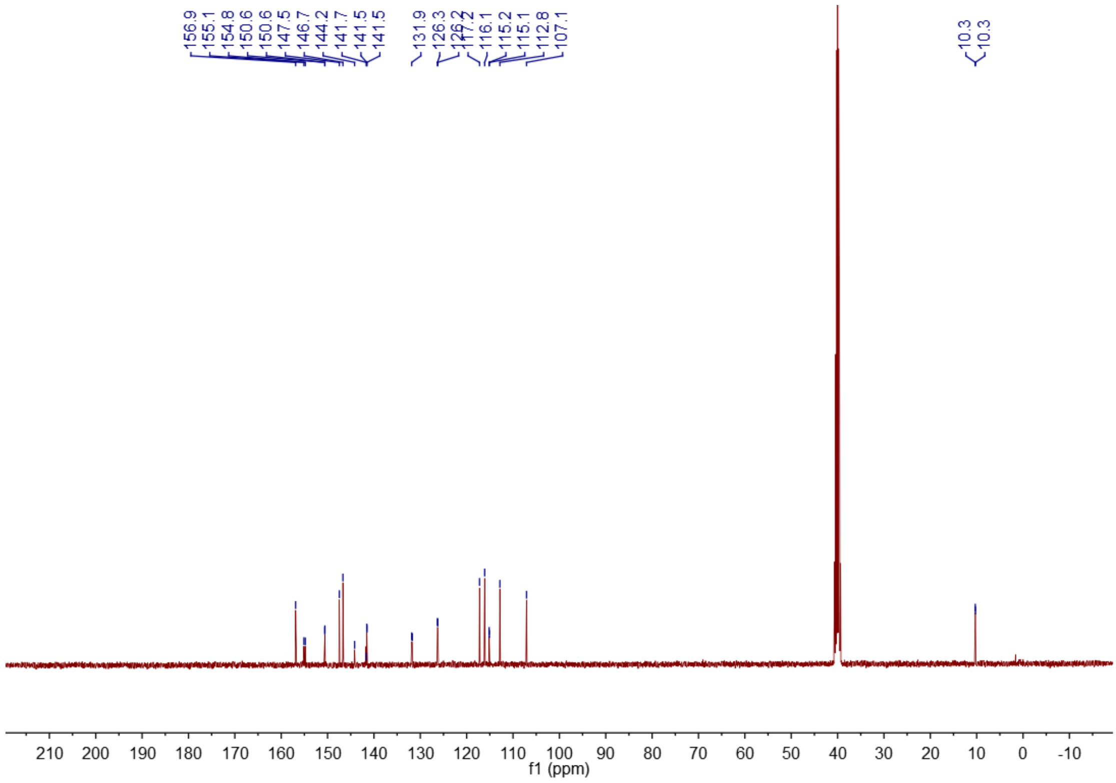

HRMS Spectrum of 5d

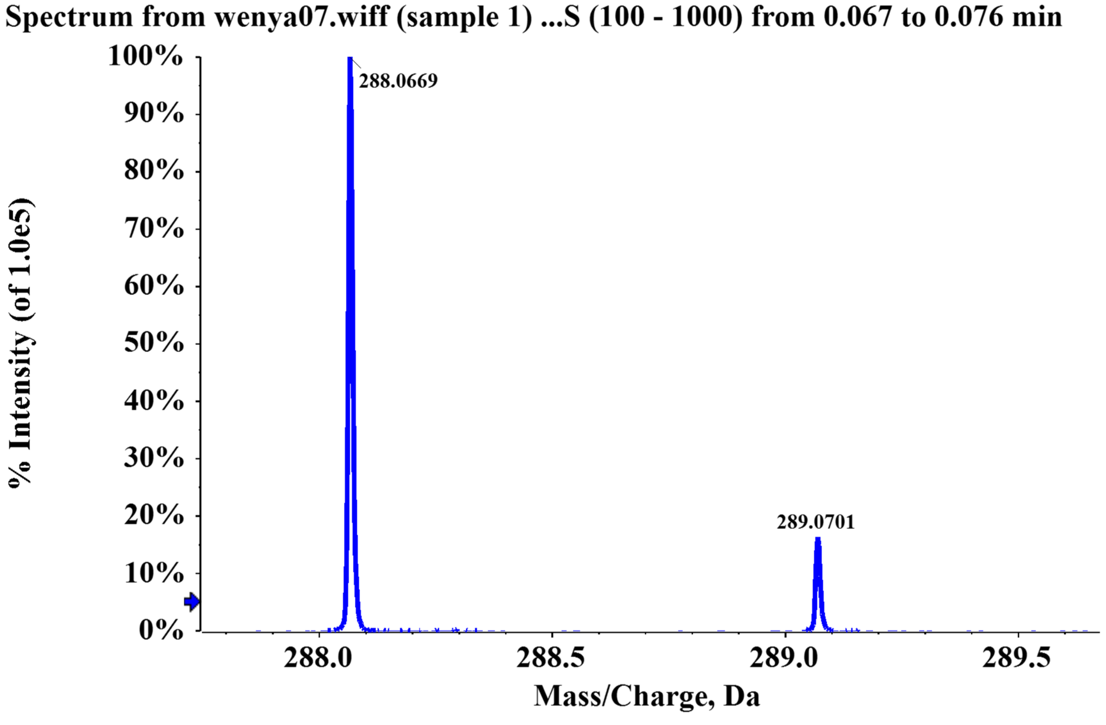

^{1}H NMR Spectrum of 5e (400 MHz, DMSO-d_6)

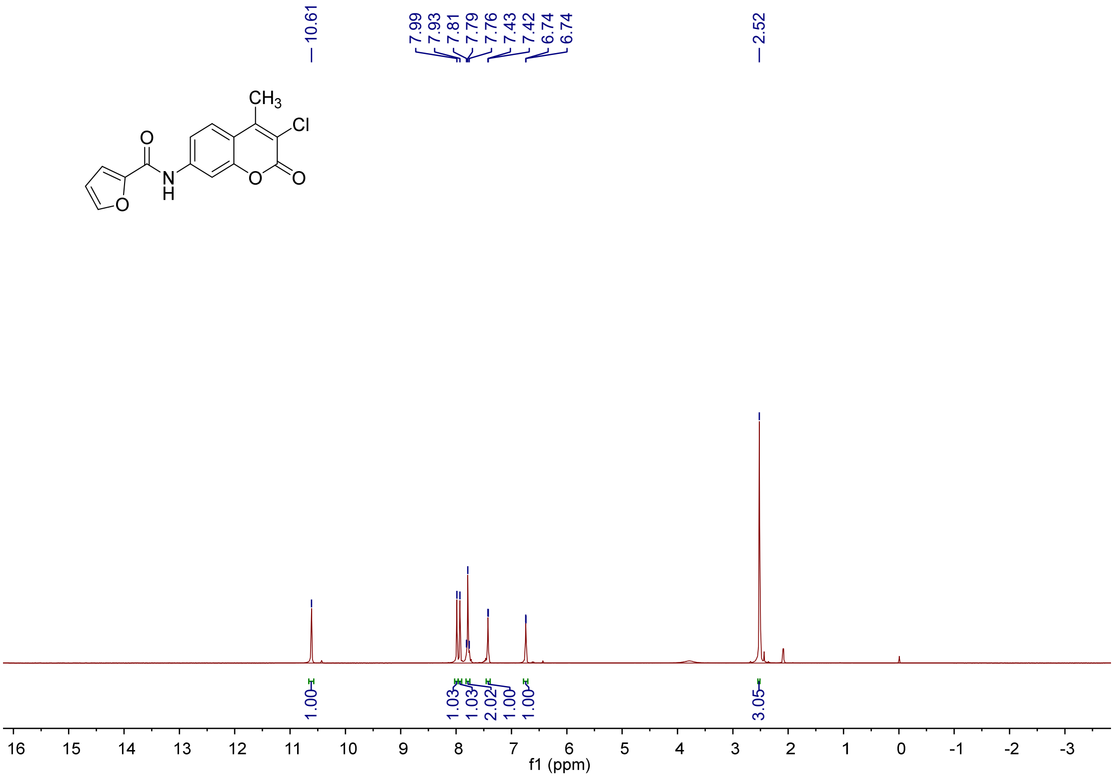

^{13}C NMR Spectrum of 5e (101 MHz, DMSO-d_6)

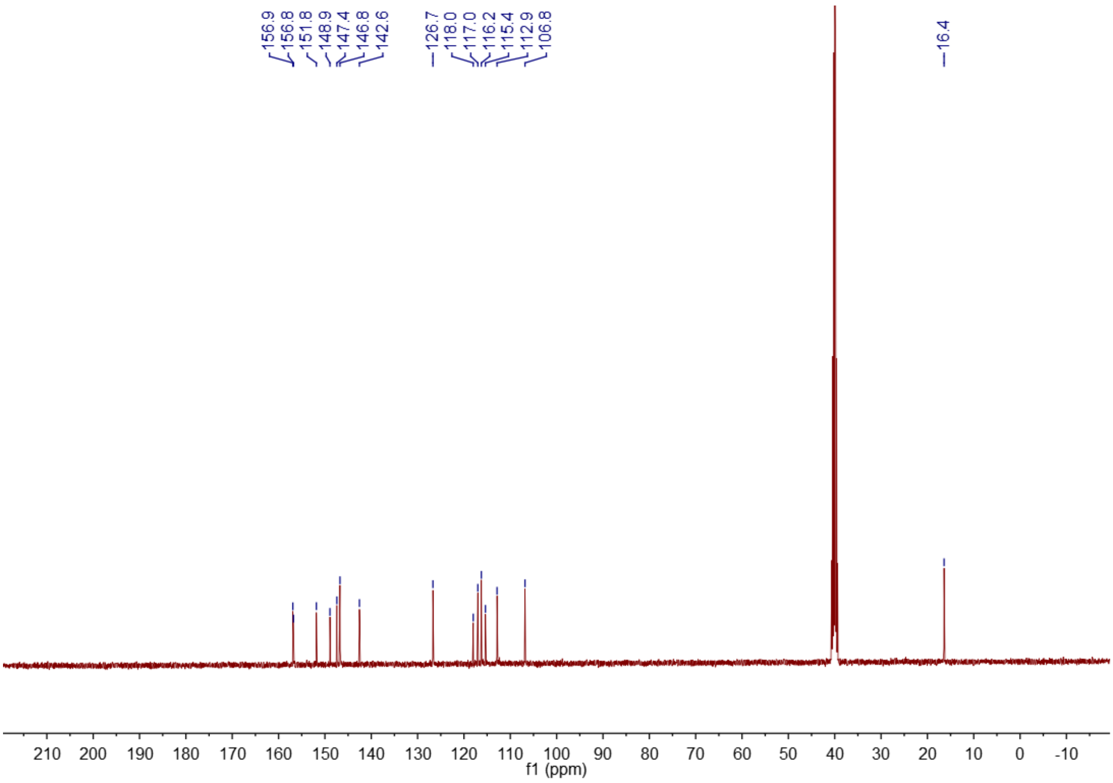

HRMS Spectrum of 5e

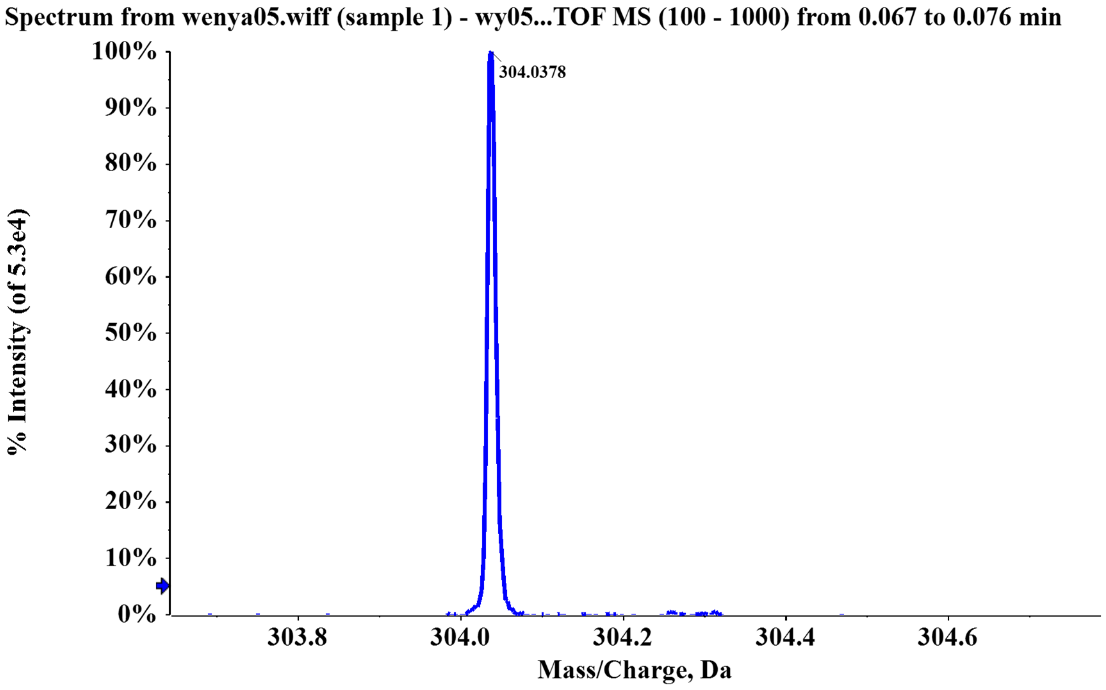

^{1}H NMR Spectrum of 5f (400 MHz, DMSO-d_6)

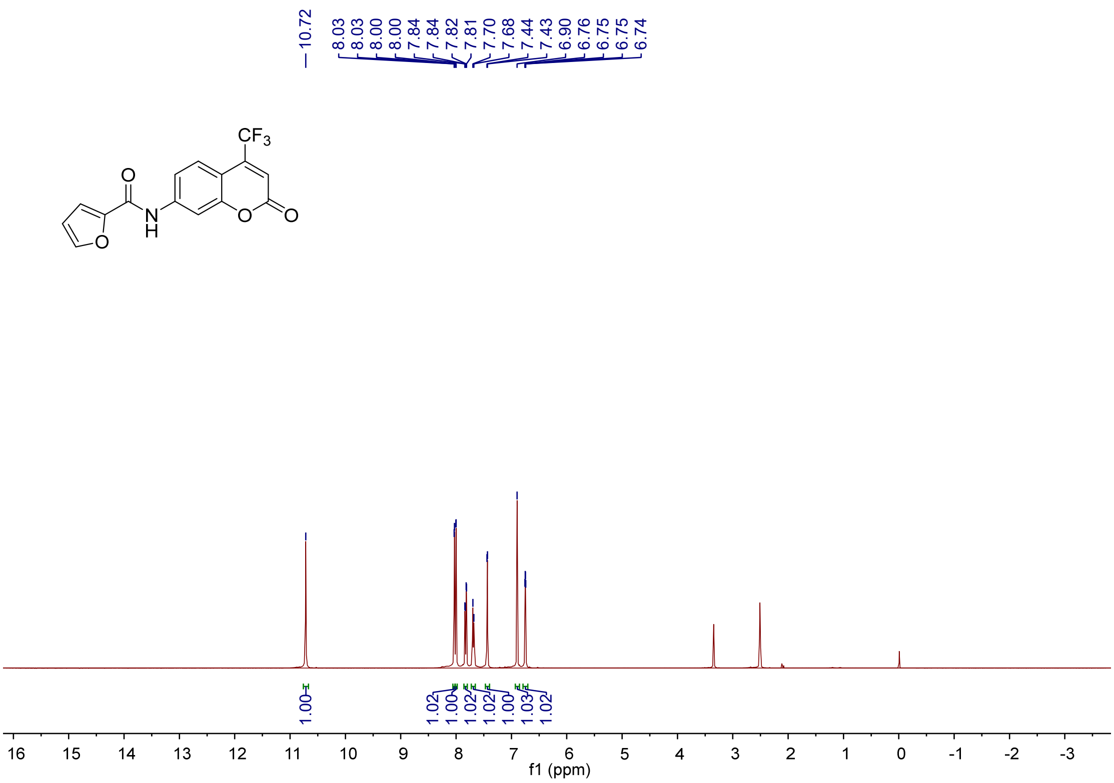

^{13}C NMR Spectrum of 5f (126 MHz, DMSO-d_6)

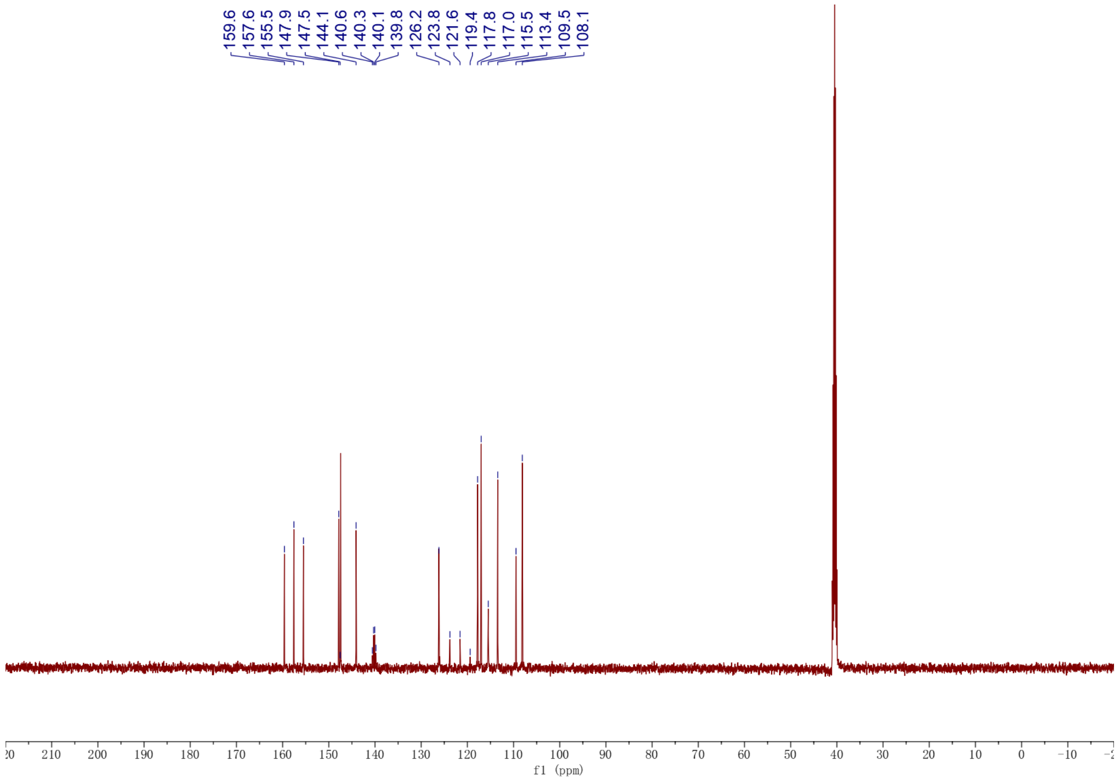

HRMS Spectrum of 5f

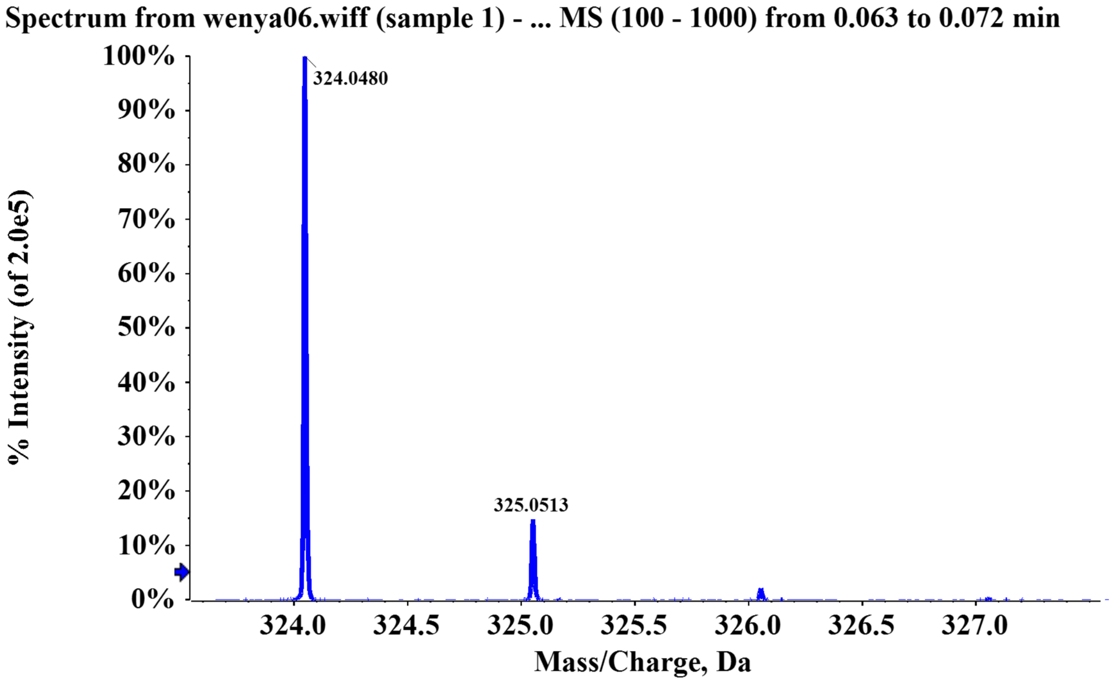

^{1}H NMR Spectrum of 5g (400 MHz, DMSO-d_6)

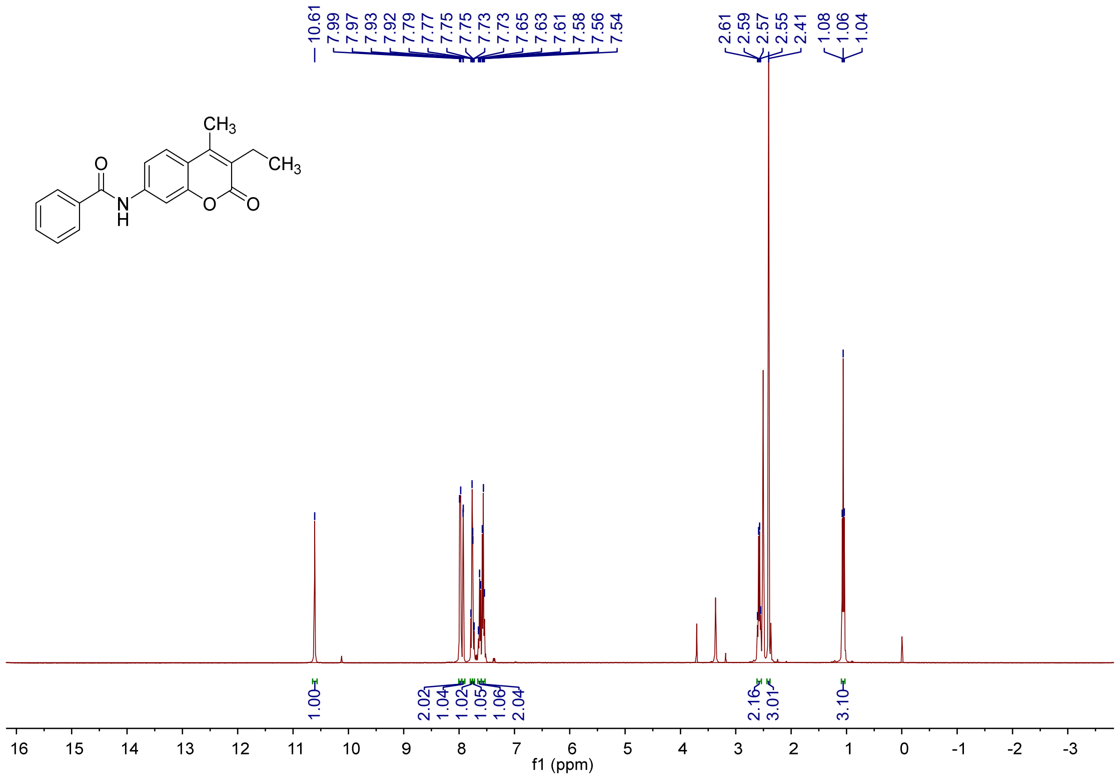

^{13}C NMR Spectrum of 5g (101 MHz, DMSO-d_6)

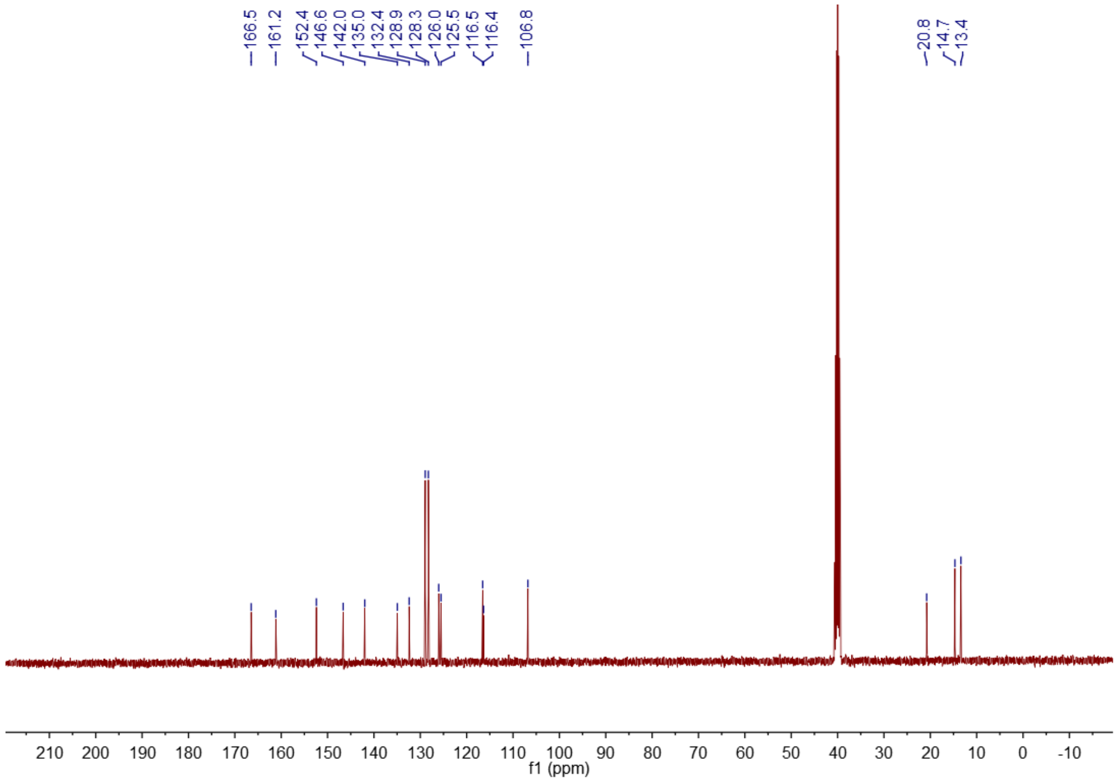

HRMS Spectrum of 5g

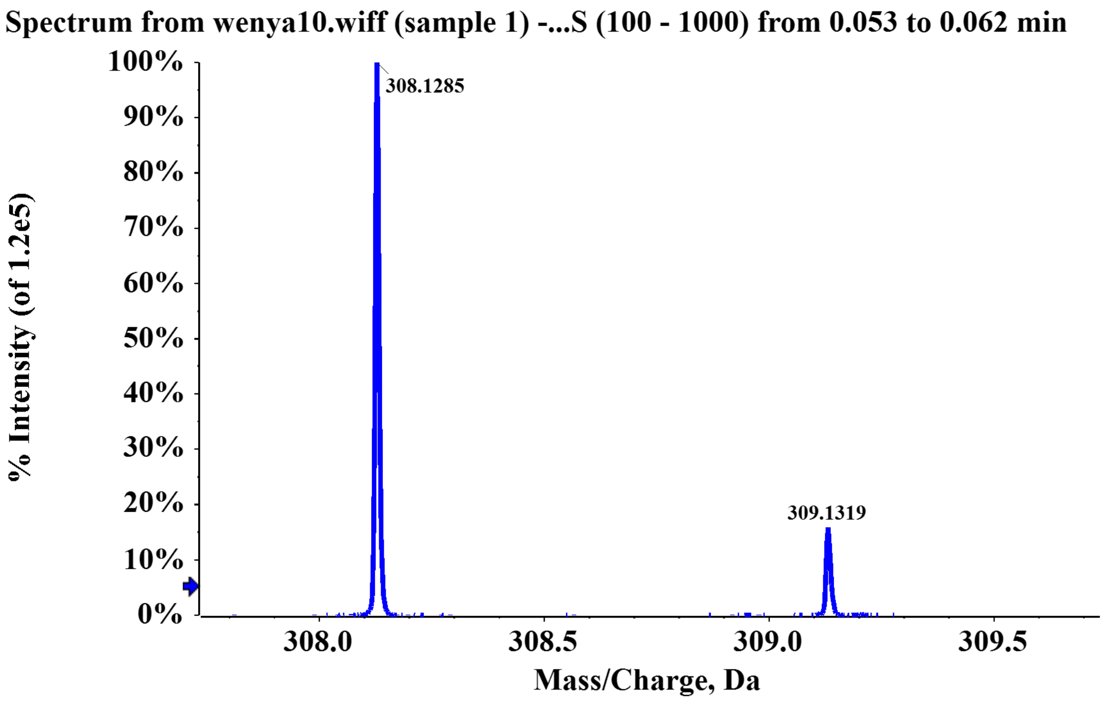

^{1}H NMR Spectrum of 5h (400 MHz, DMSO-d_6)

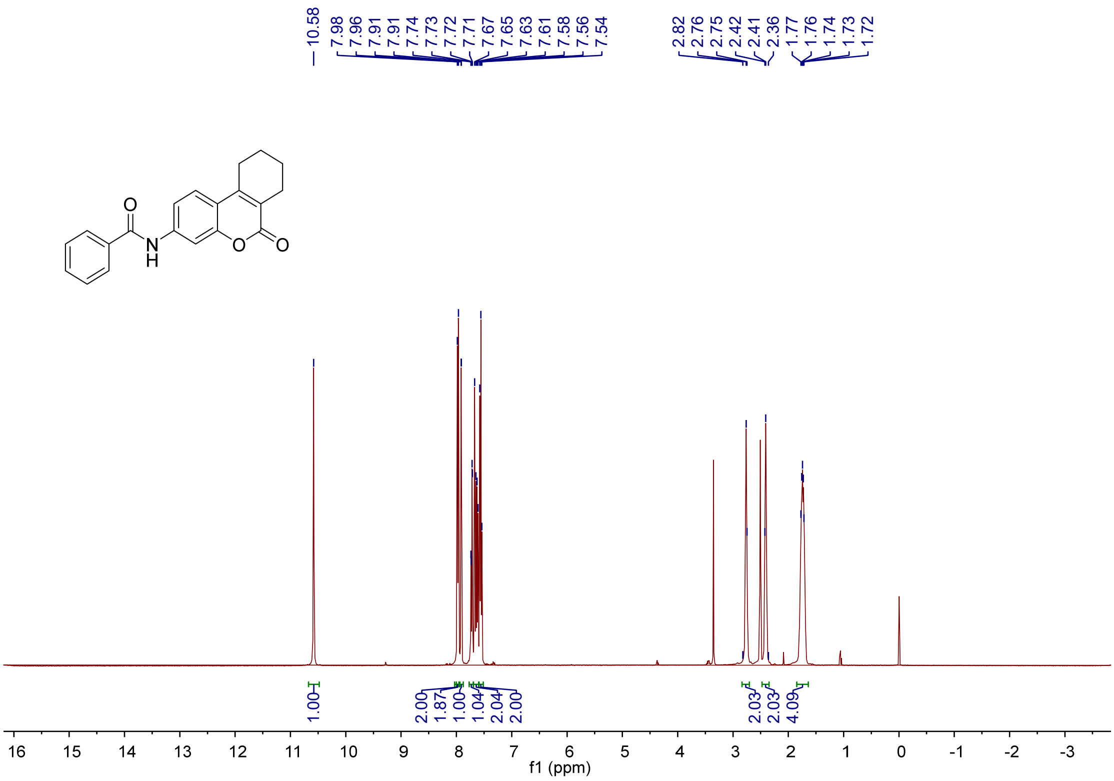

^{13}C NMR Spectrum of 5h (101 MHz, DMSO-d_6)

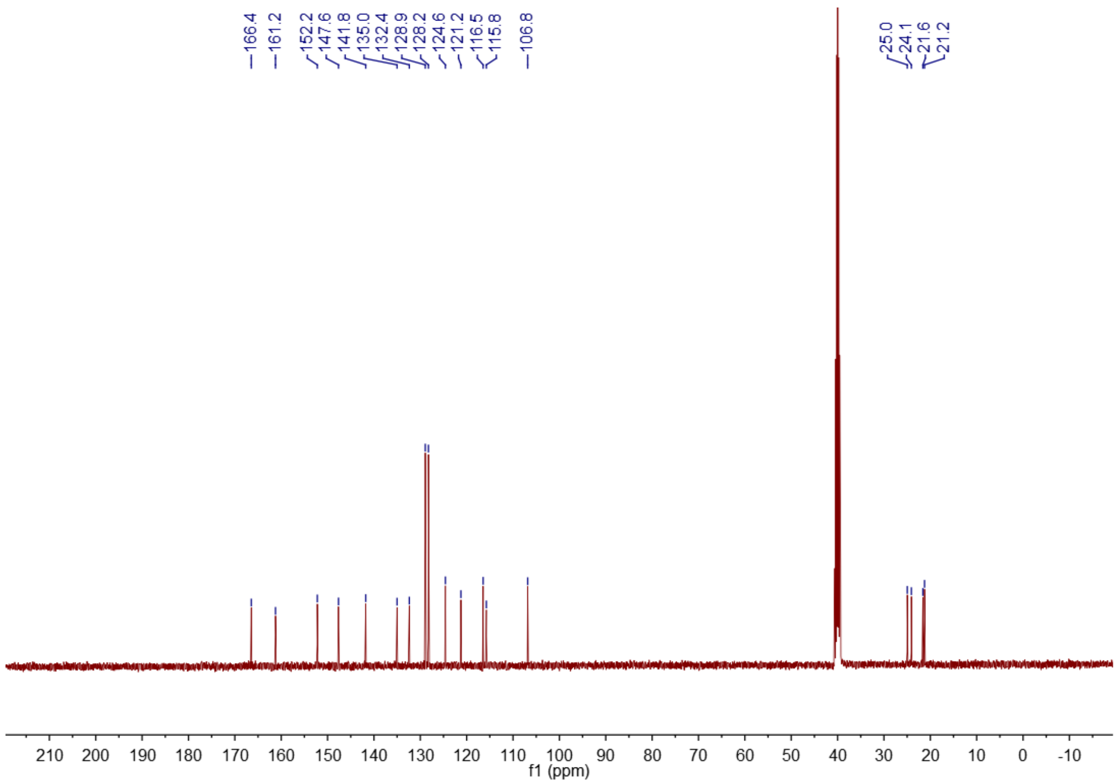

HRMS Spectrum of 5h

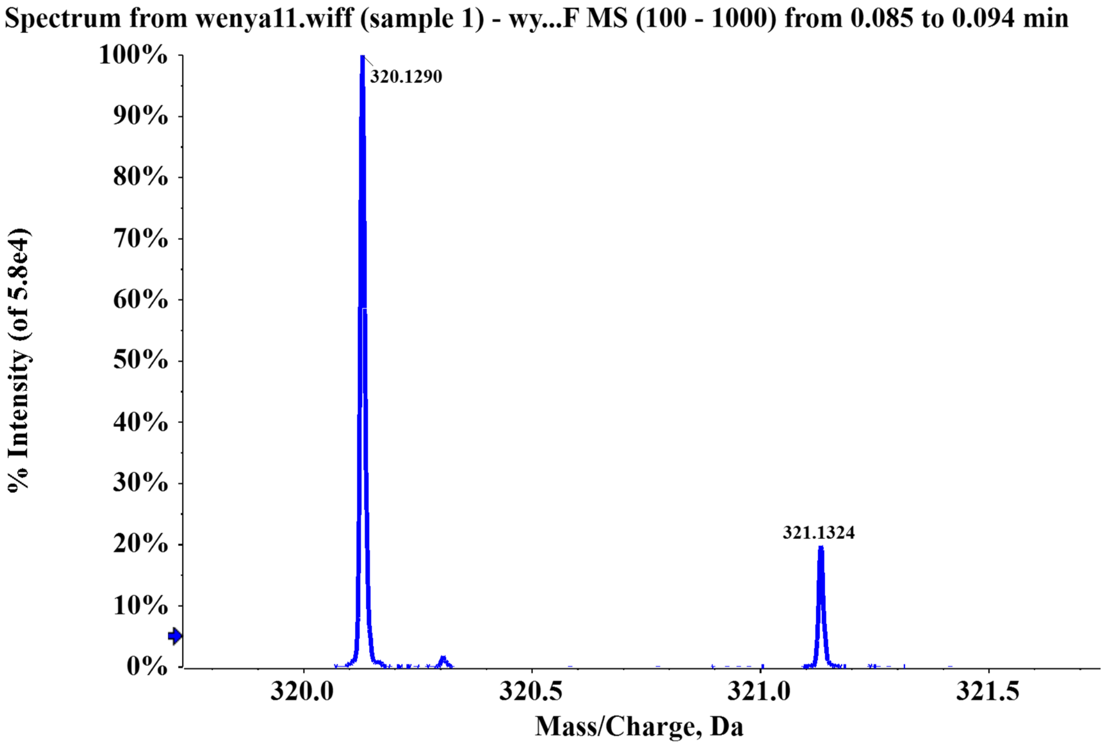

^{1}H NMR Spectrum of 5i (400 MHz, DMSO-d_6)

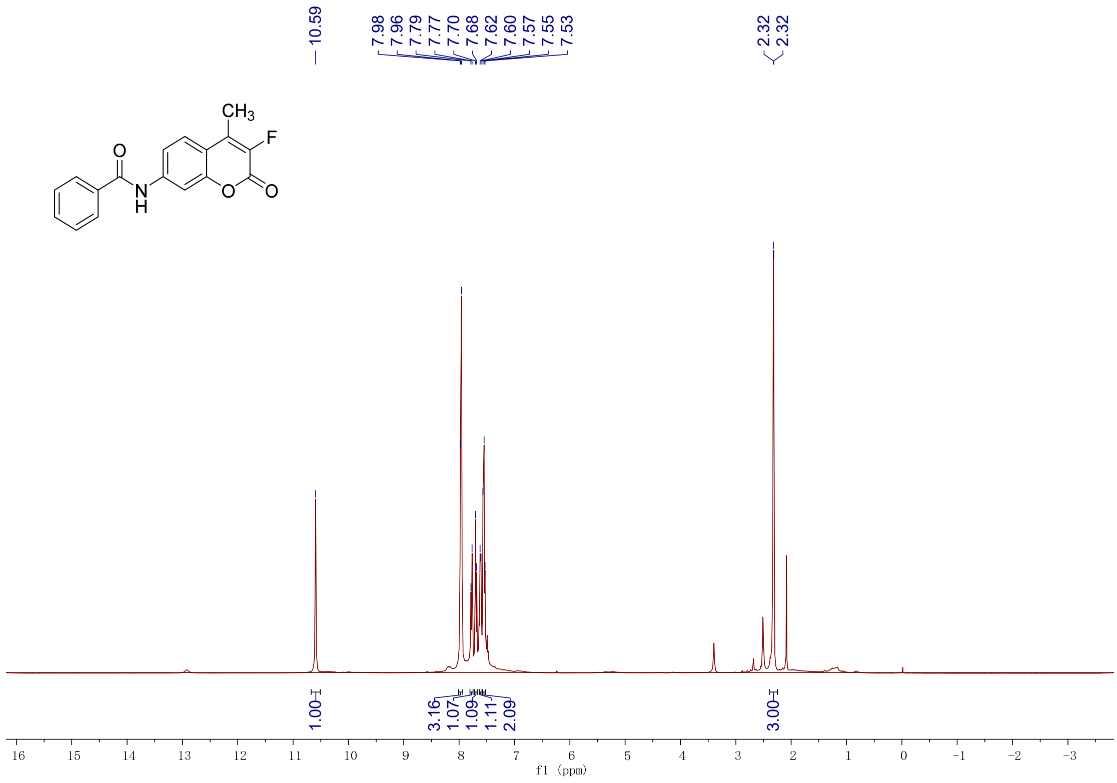

^{13}C NMR Spectrum of 5i (101 MHz, DMSO-d_6)

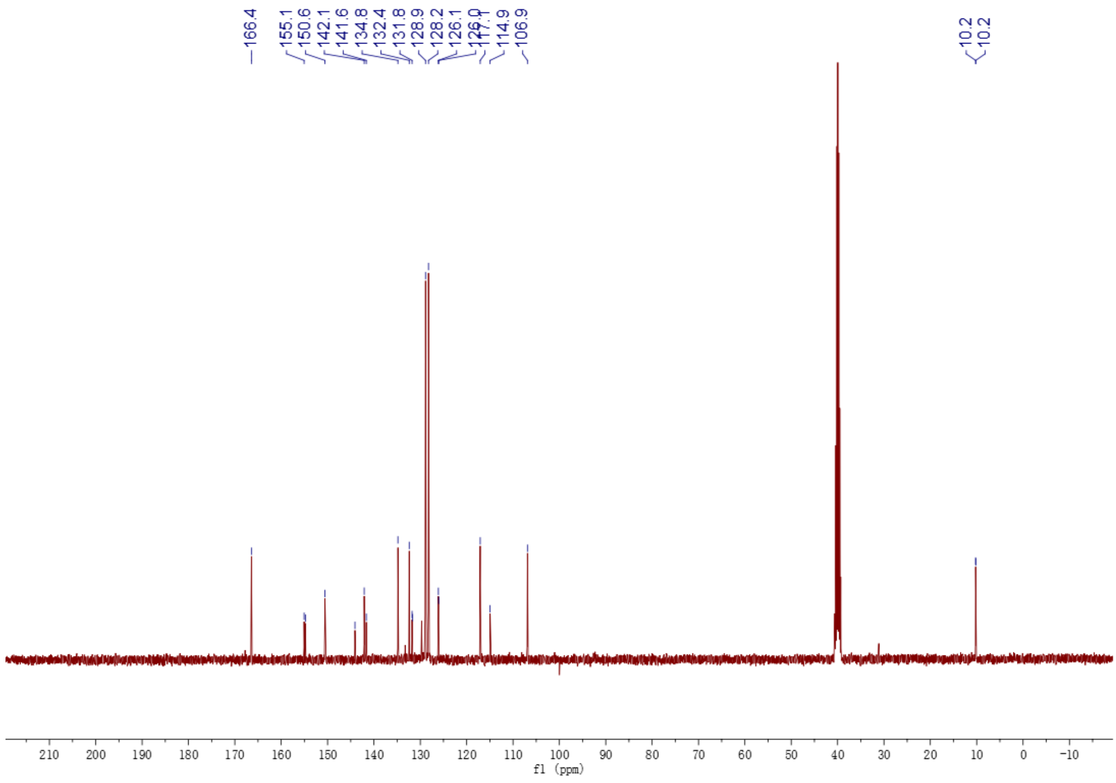

HRMS Spectrum of 5i

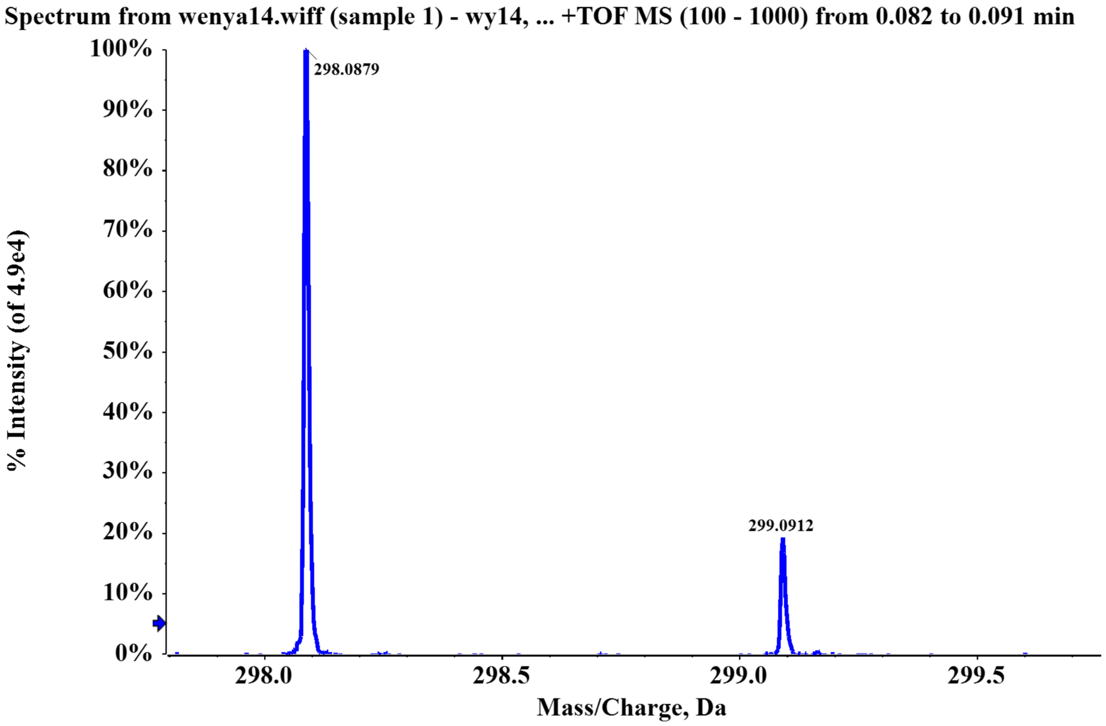

^{1}H NMR Spectrum of 5j (400 MHz, DMSO-d_6)

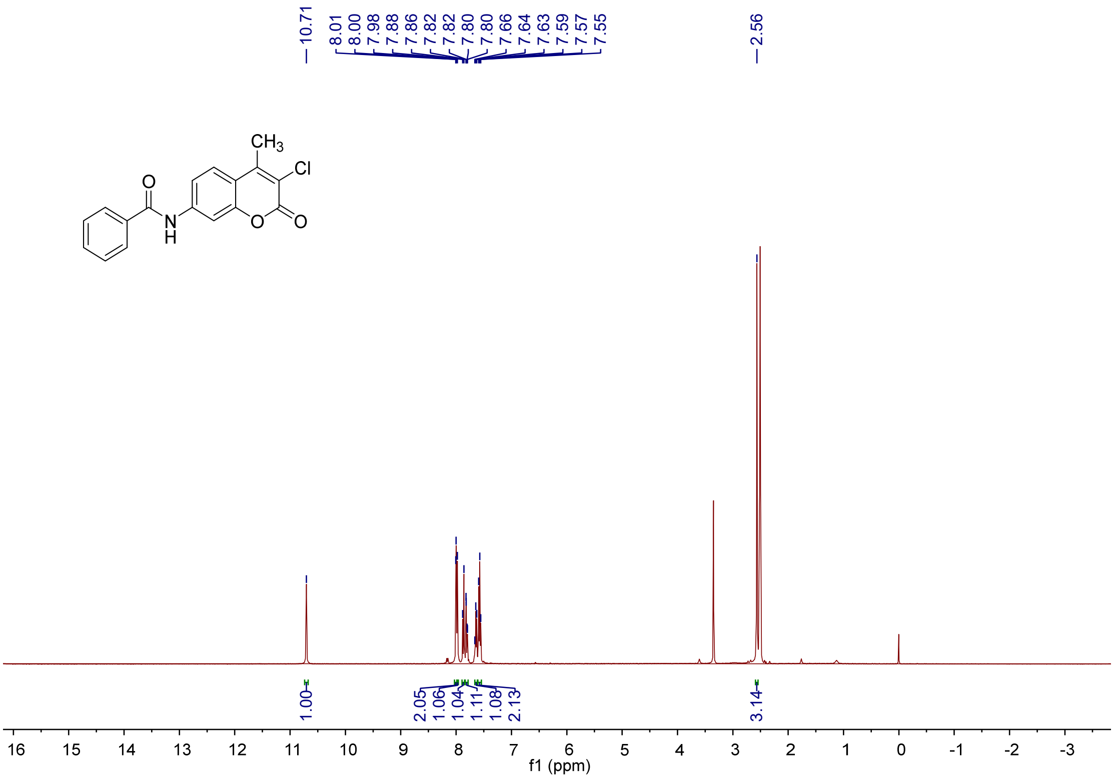

^{13}C NMR Spectrum of 5j (101 MHz, DMSO-d_6)

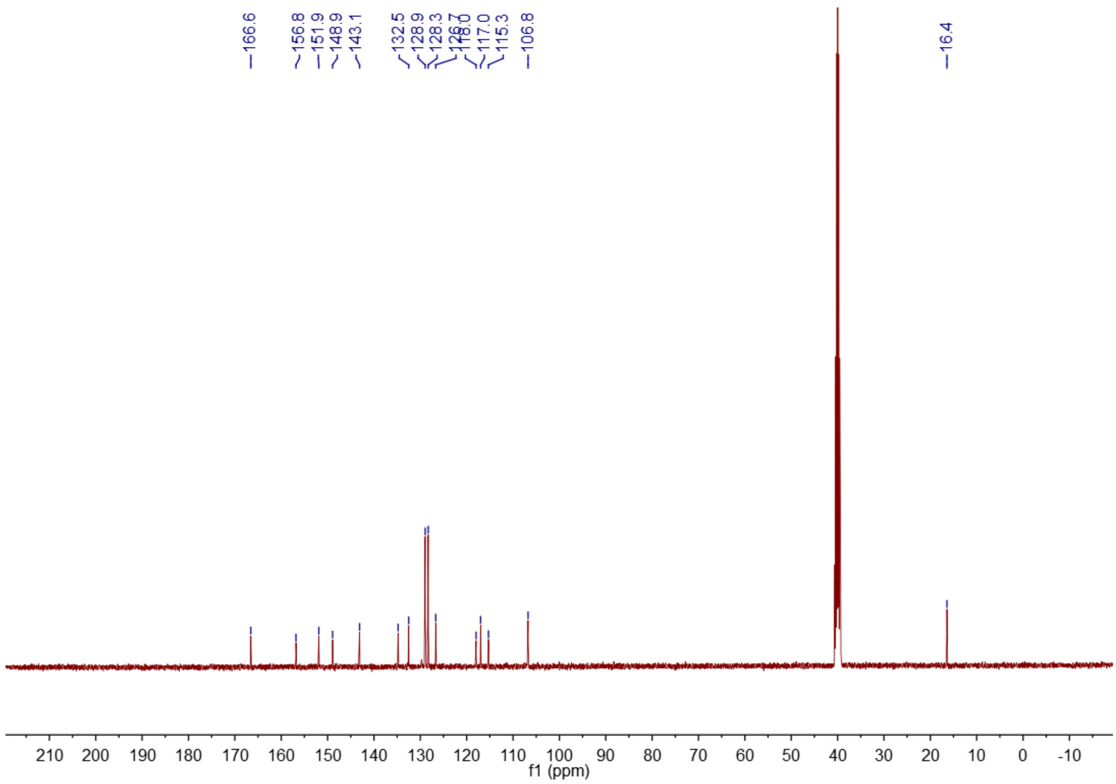

HRMS Spectrum of 5j

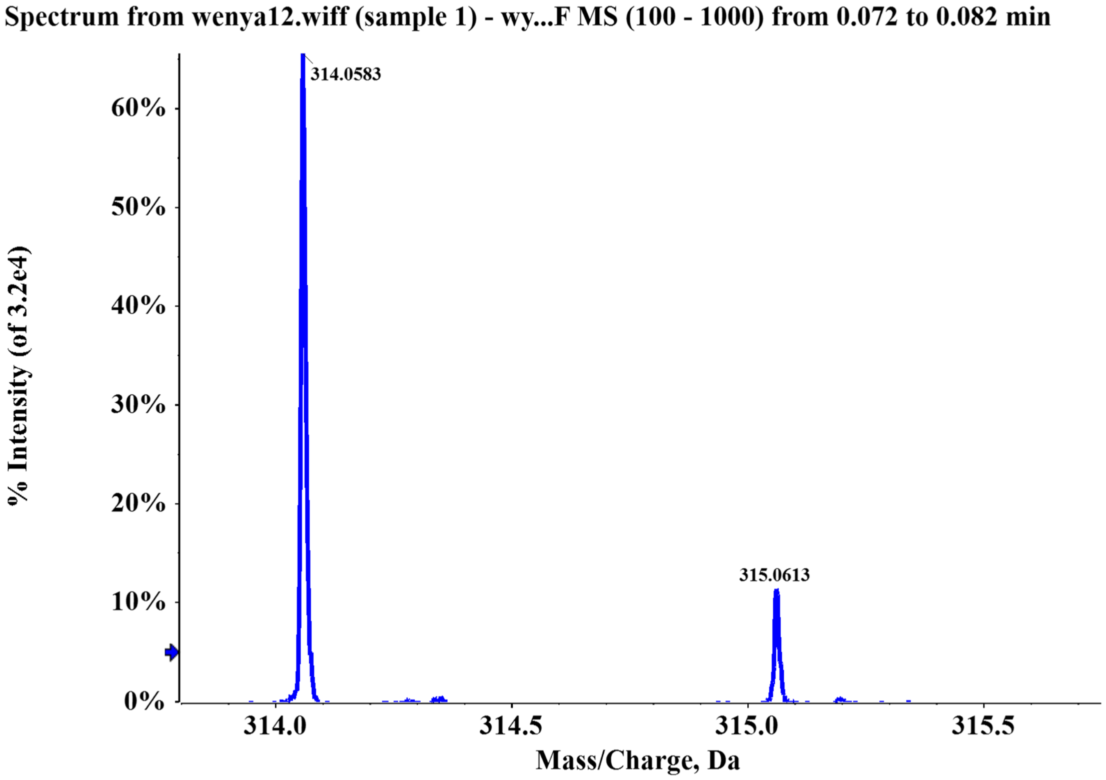

^{1}H NMR Spectrum of 5k (400 MHz, DMSO-d_6)

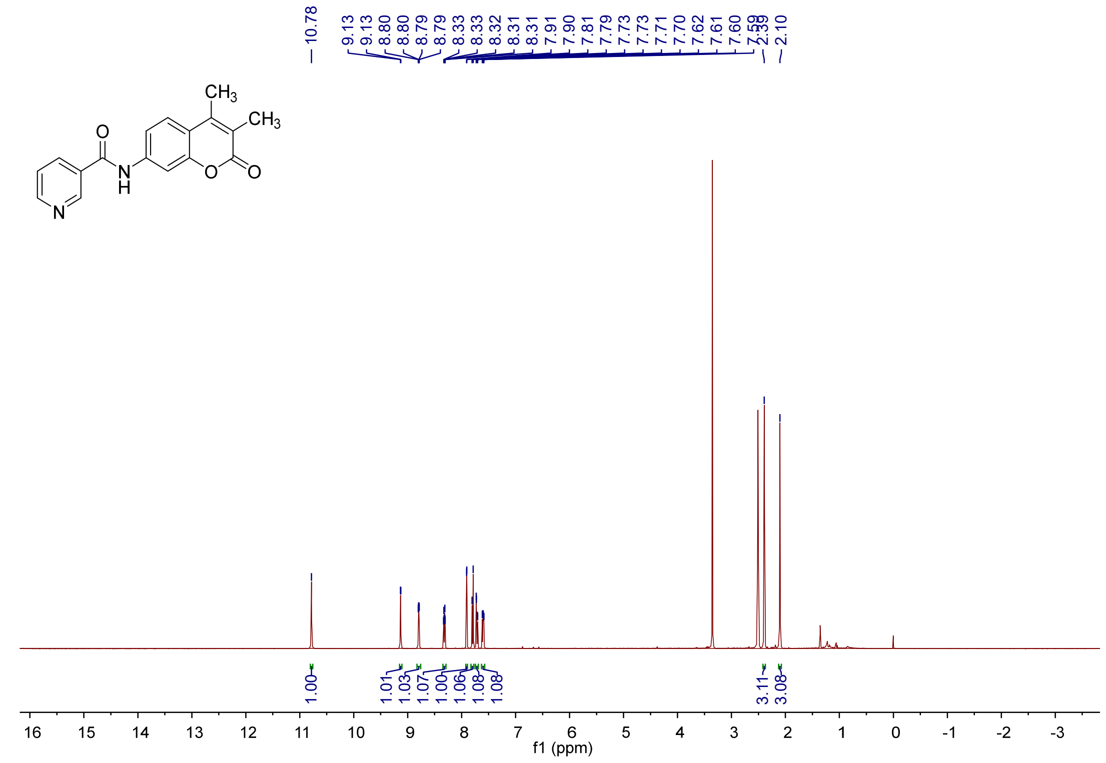

^{13}C NMR Spectrum of 5k (101 MHz, DMSO-*d*$_6$)

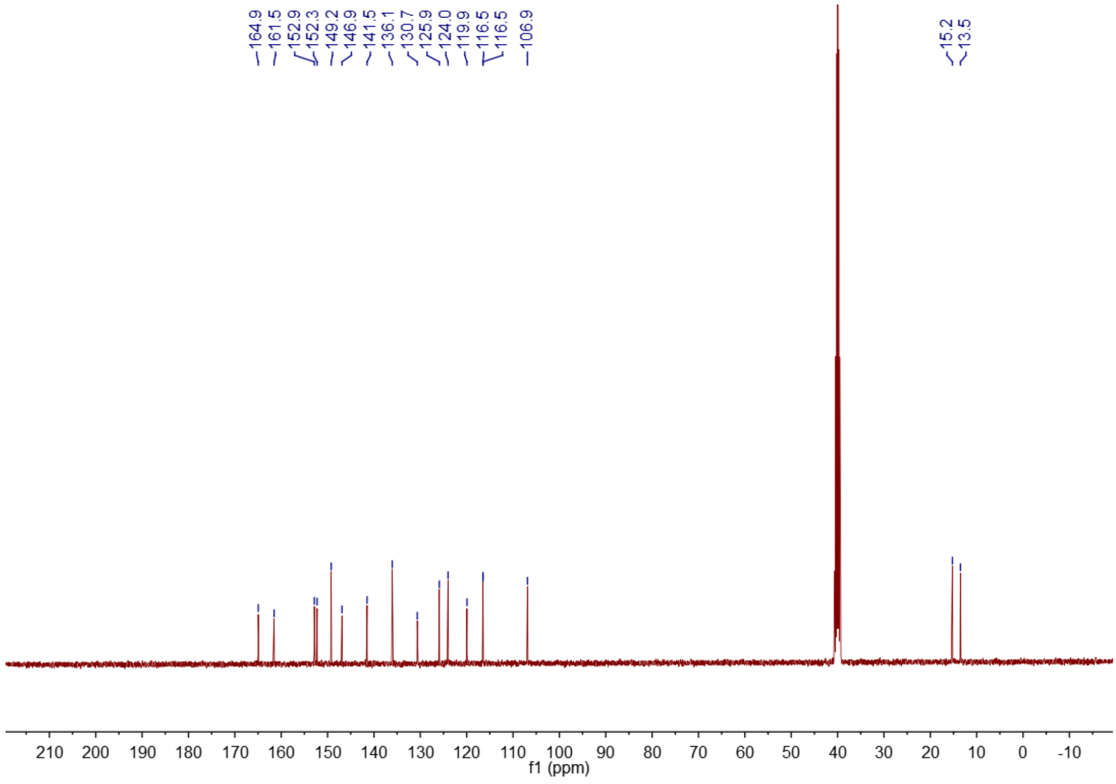

HRMS Spectrum of 5k

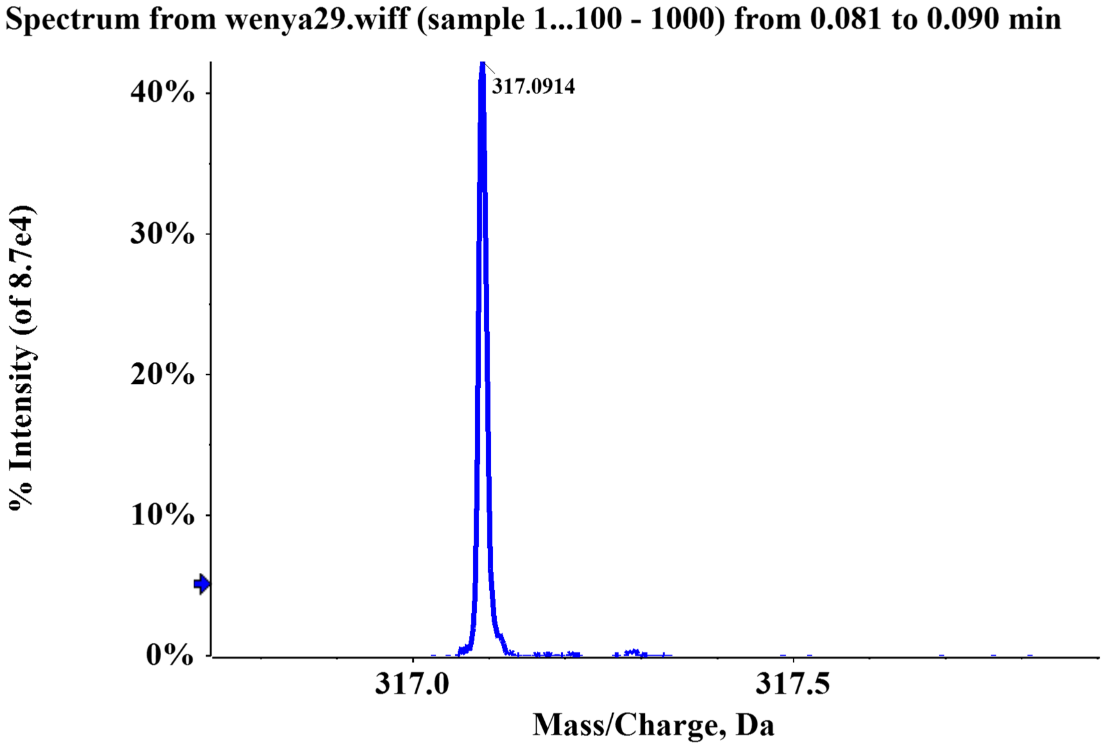

^{1}H NMR Spectrum of 5l (400 MHz, DMSO-d_6)

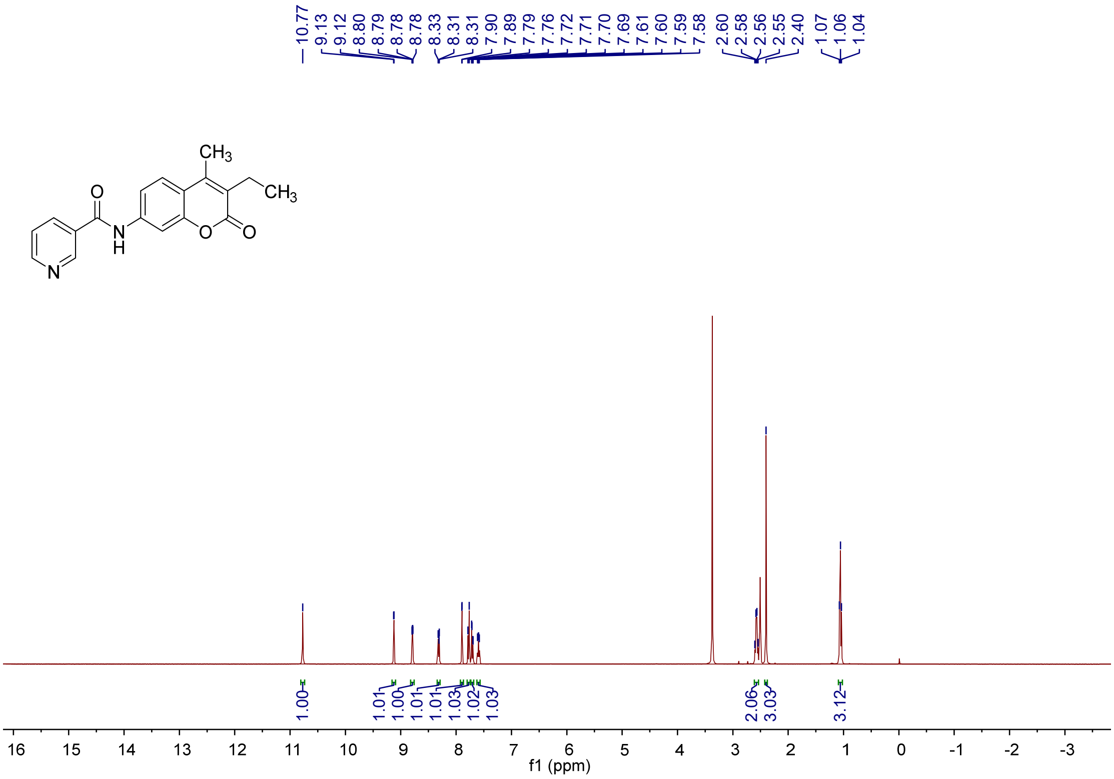

^{13}C NMR Spectrum of 5l (101 MHz, DMSO-d_6)

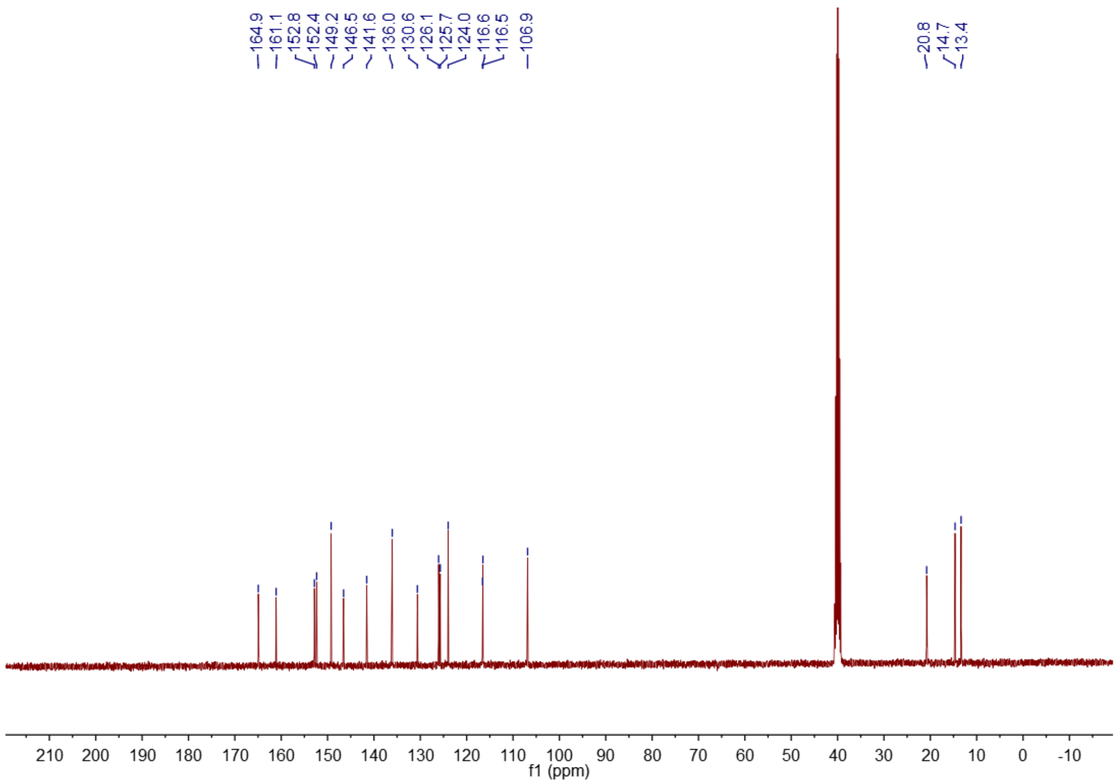

HRMS Spectrum of 5l

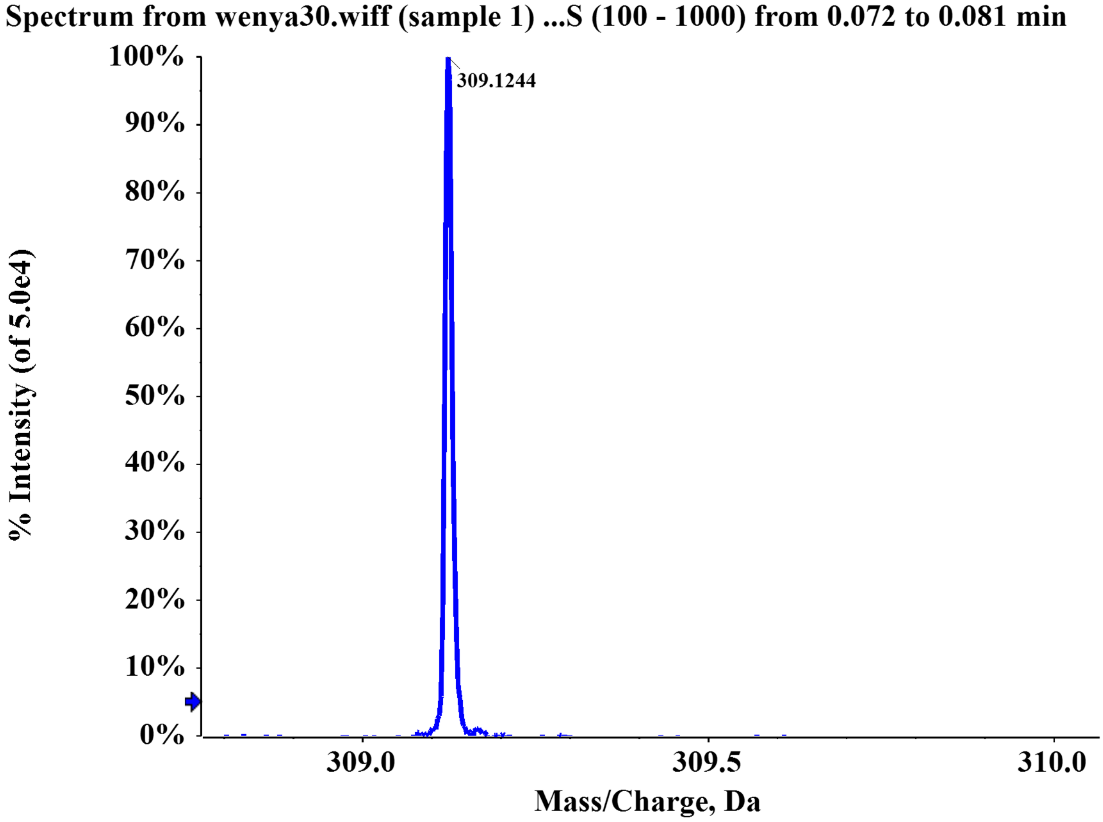

^{1}H NMR Spectrum of 5m (400 MHz, DMSO-d_6)

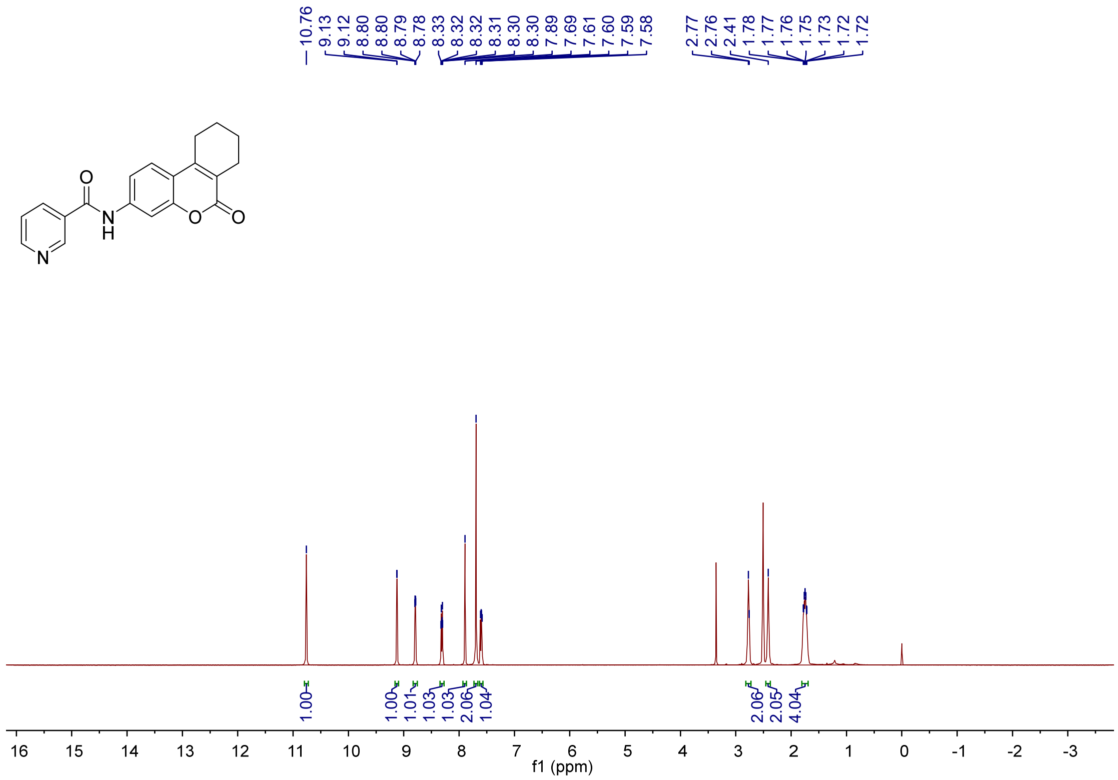

^{13}C NMR Spectrum of 5m (101 MHz, DMSO-d_6)

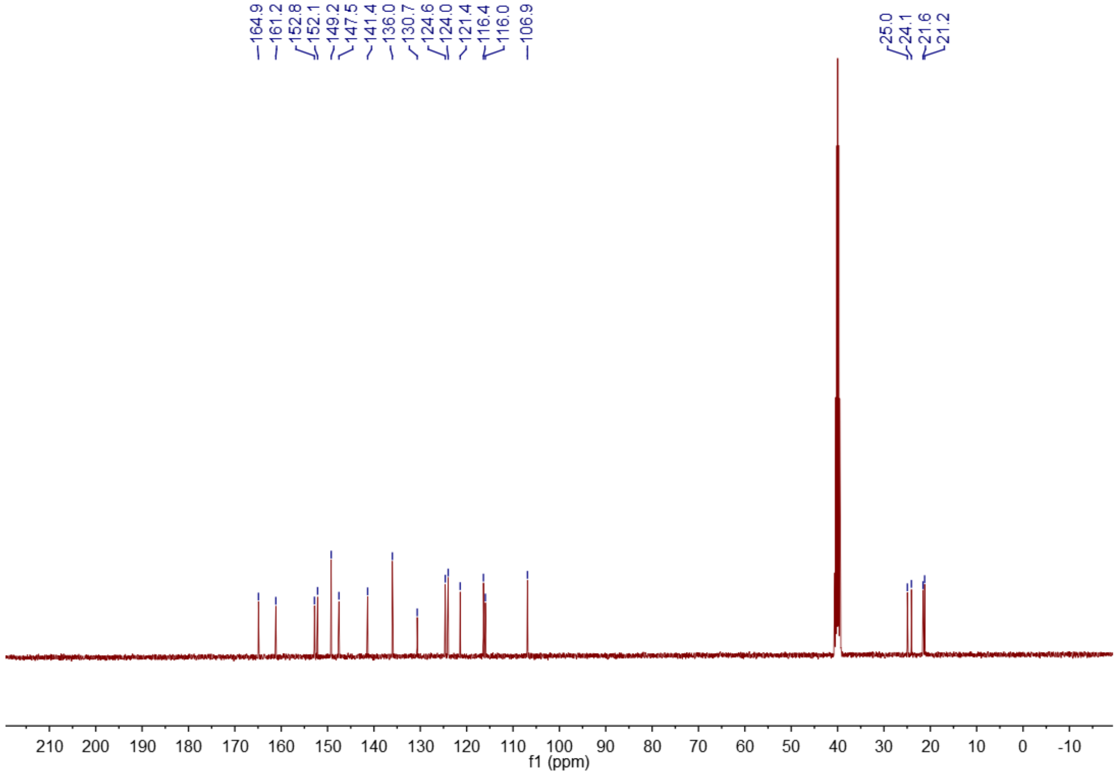

HRMS Spectrum of 5m

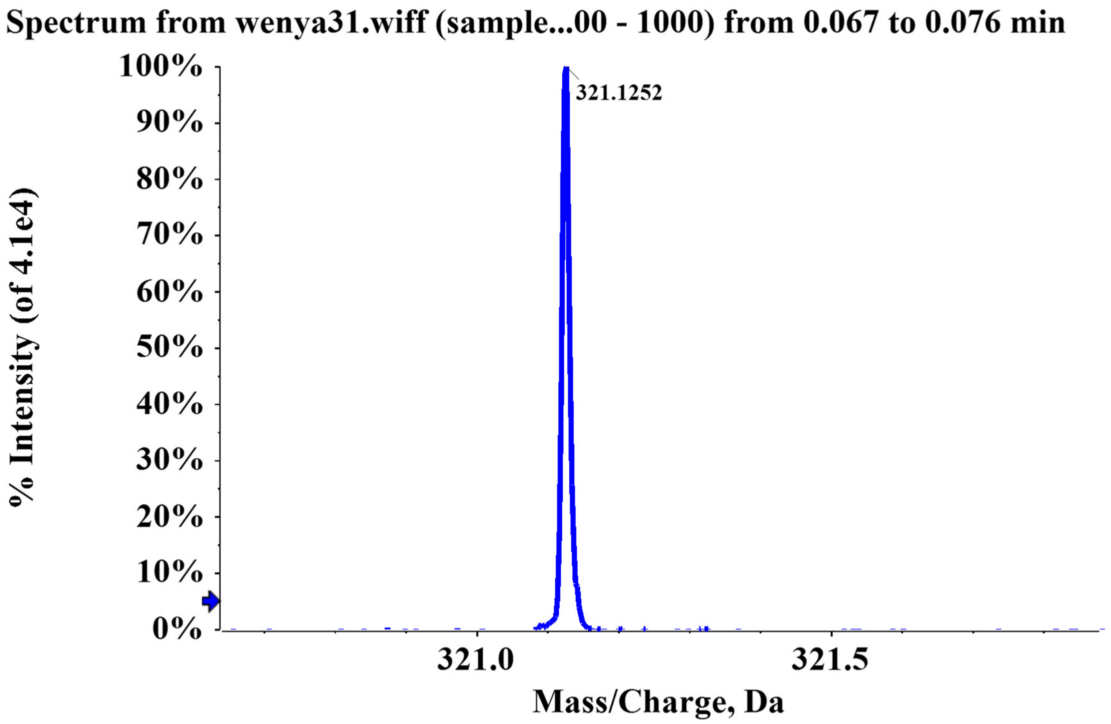

^{1}H NMR Spectrum of 5n (400 MHz, DMSO-d_6)

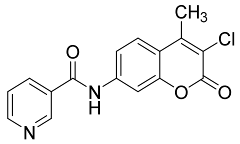

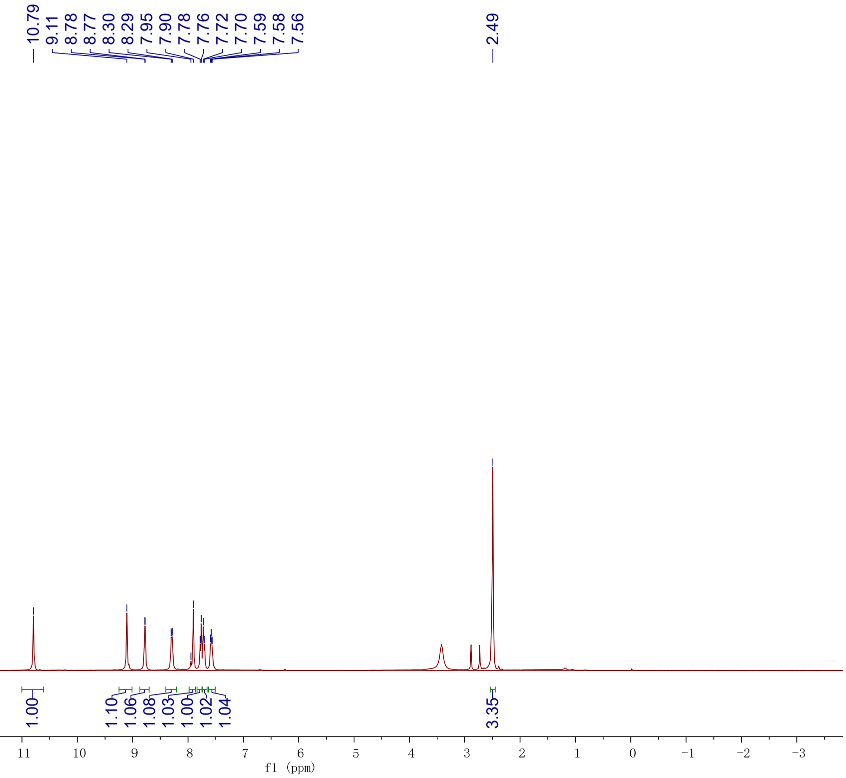

^{13}C NMR Spectrum of 5n (101 MHz, DMSO-d_6)

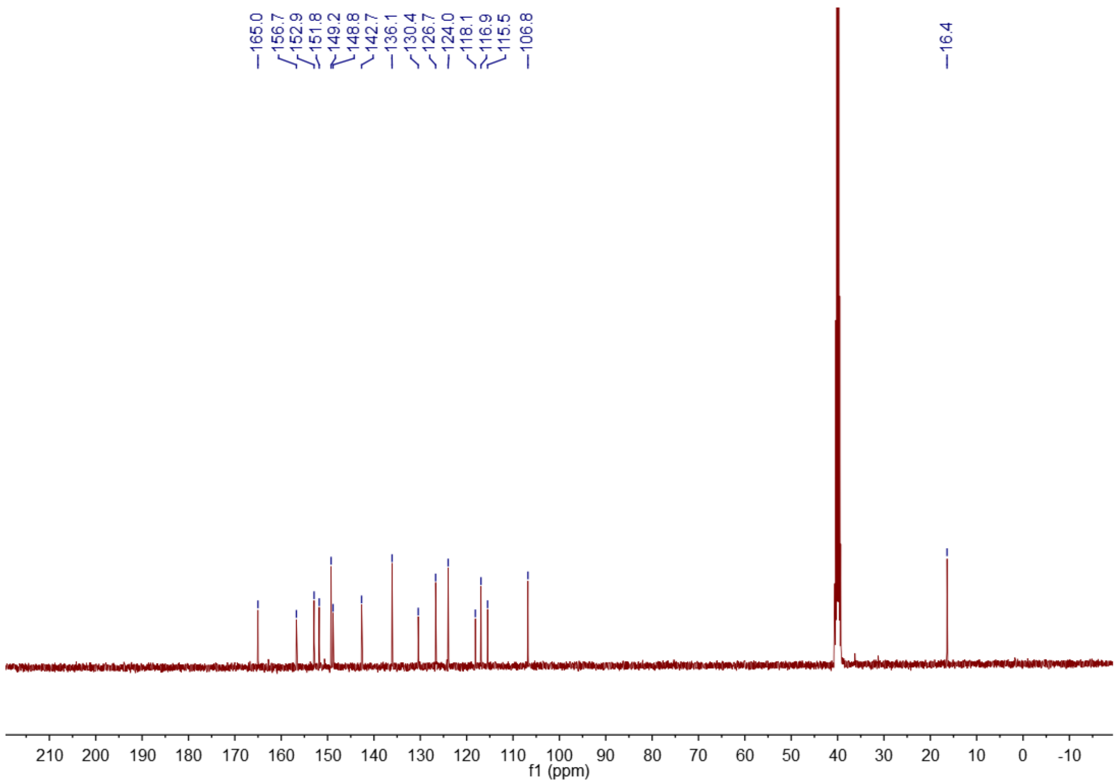

HRMS Spectrum of 5n

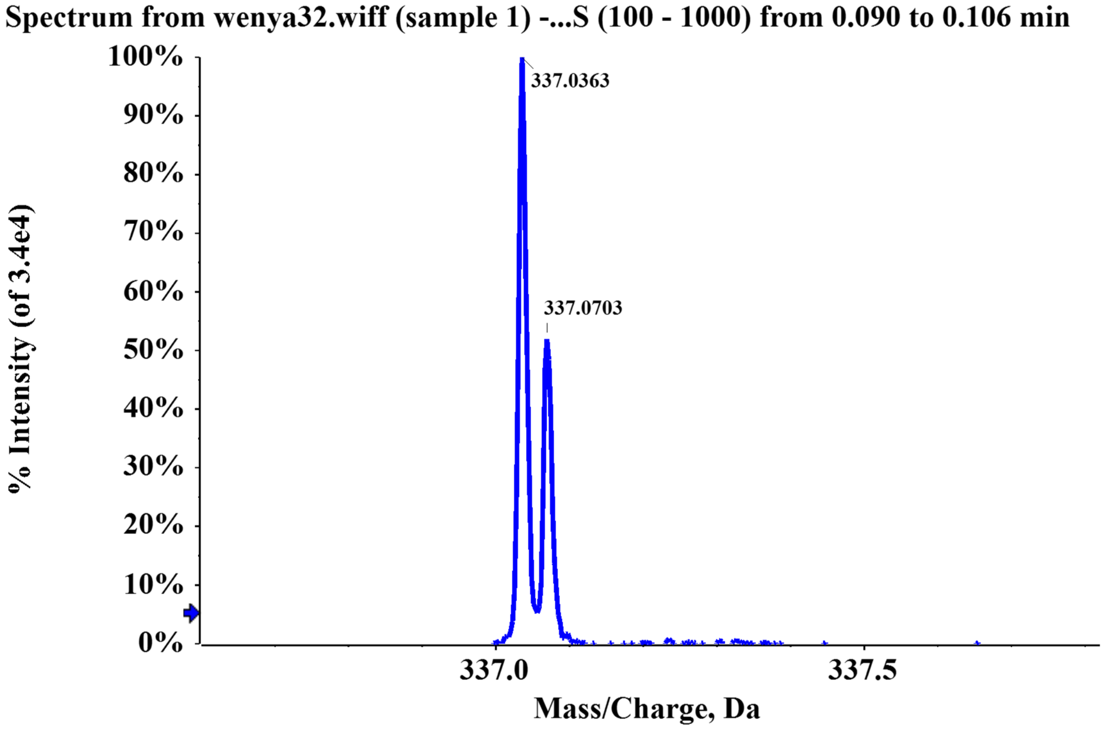

^{1}H NMR Spectrum of 5o (400 MHz, DMSO-d_6)

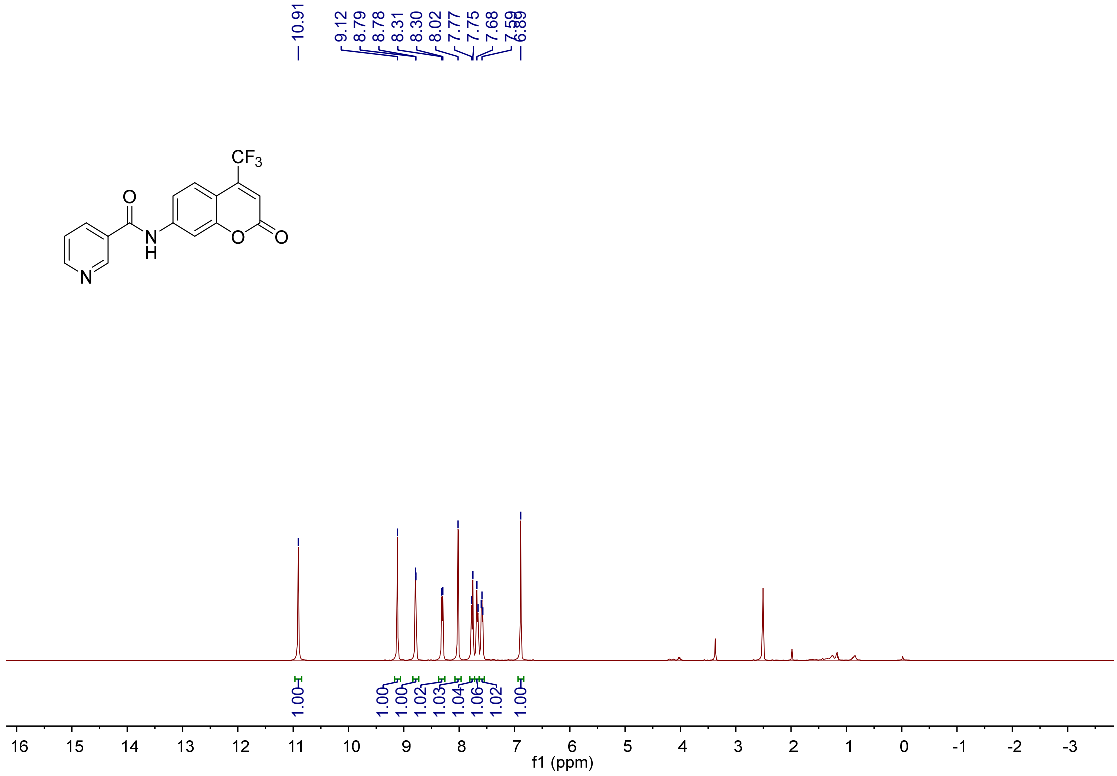

^{13}C NMR Spectrum of 5o (101 MHz, CF_3COOD)

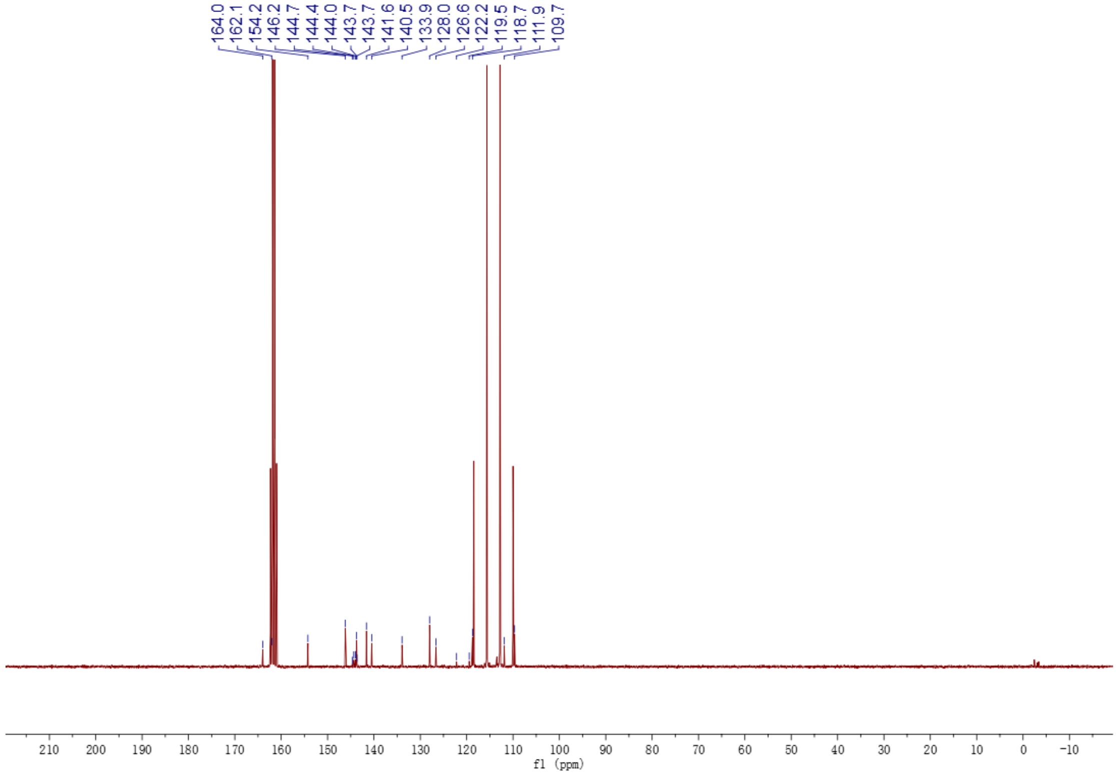

HRMS Spectrum of 5o

Spectrum from wenya33.wiff (sample...0 - 1000) from 0.067 to 0.076 min

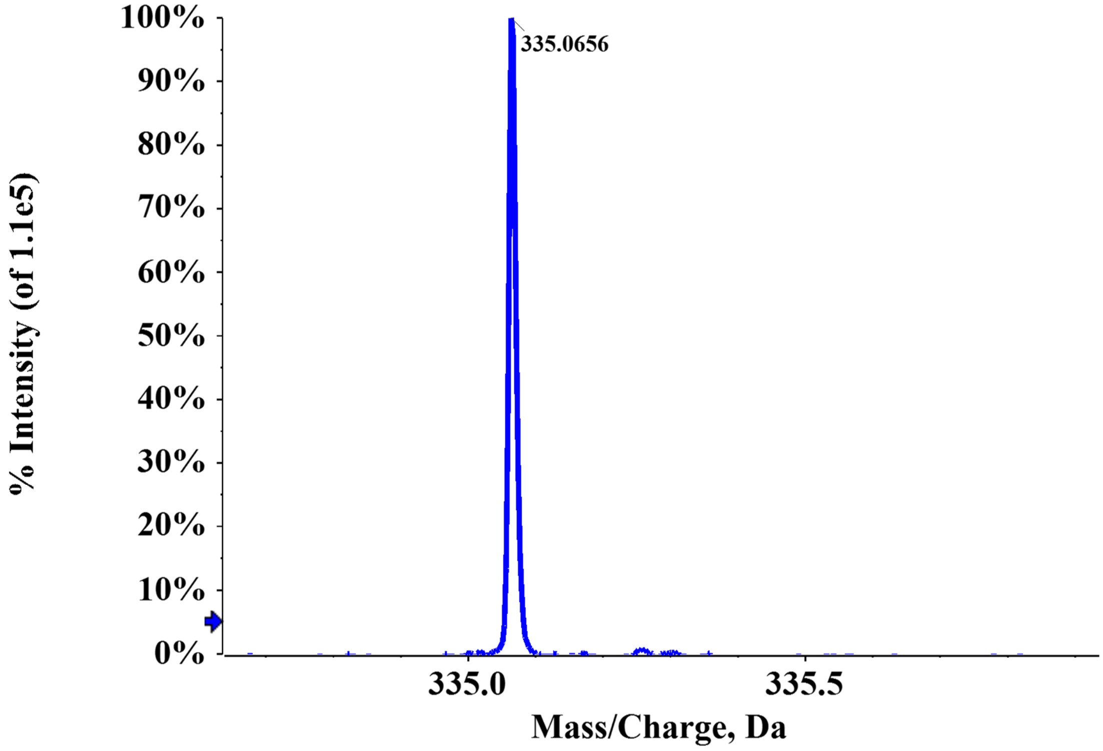

^{1}H NMR Spectrum of 5p (400 MHz, DMSO-d_6)

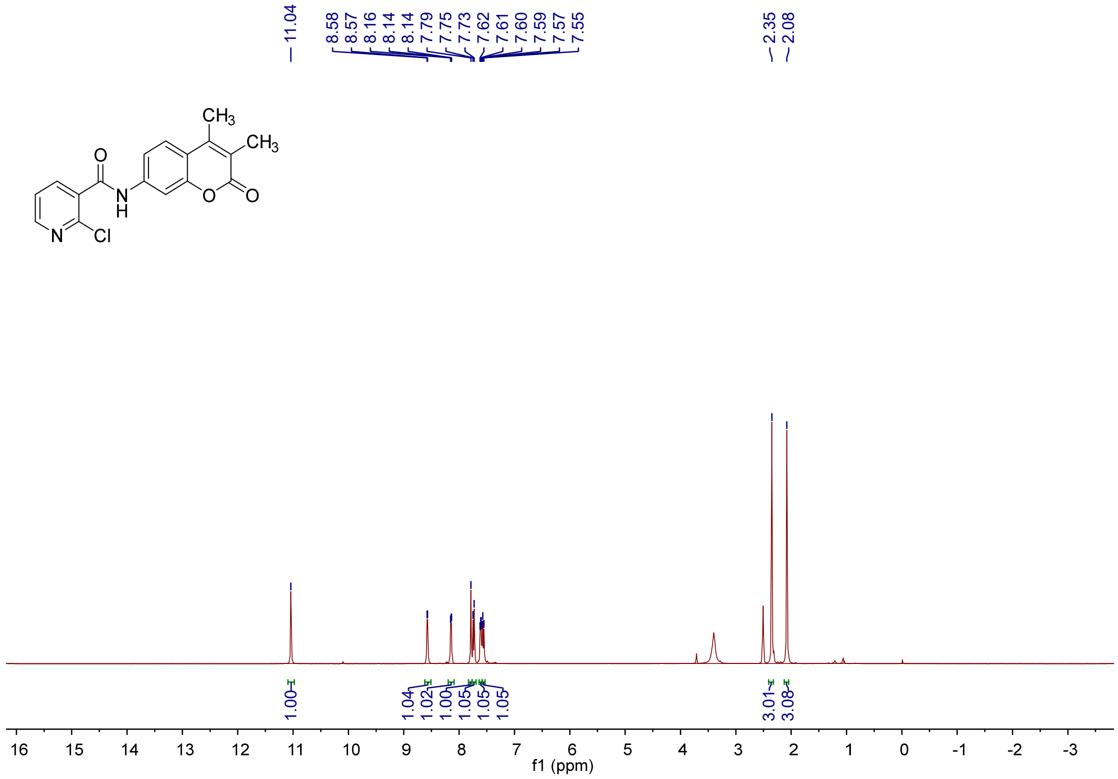

^{13}C NMR Spectrum of 5p (101 MHz, DMSO-d_6)

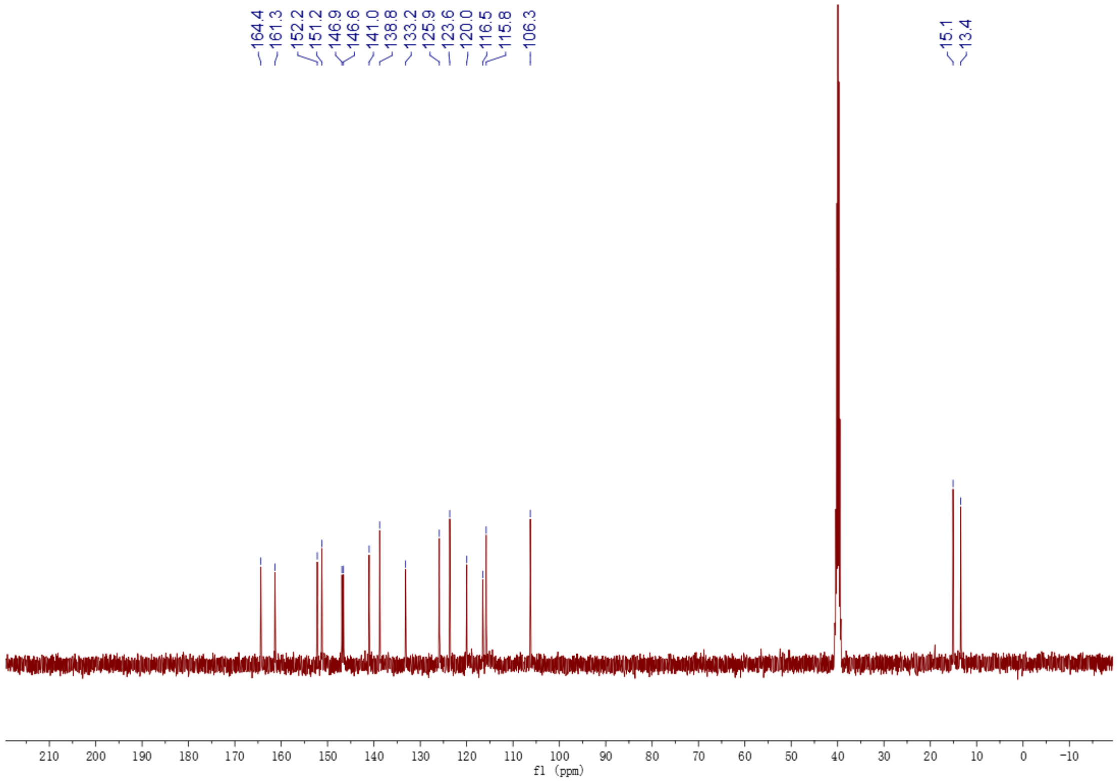

HRMS Spectrum of 5p

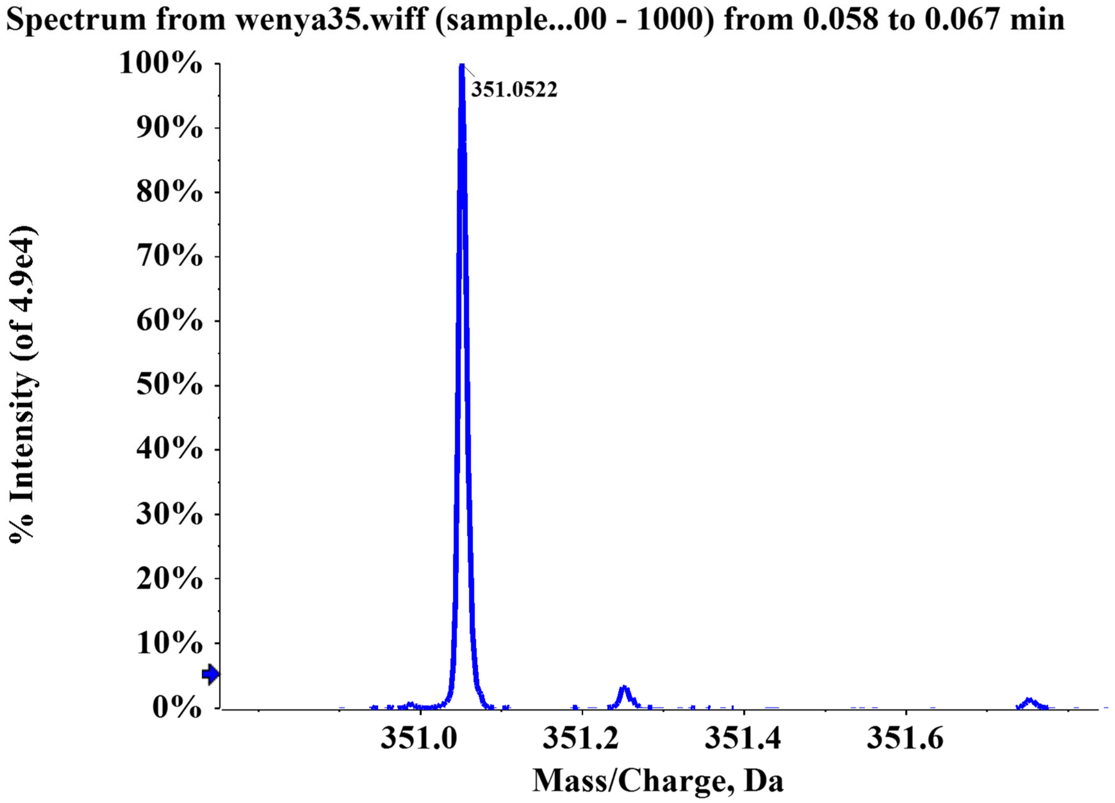

^{1}H NMR Spectrum of 5q (400 MHz, DMSO-*d*$_6$)

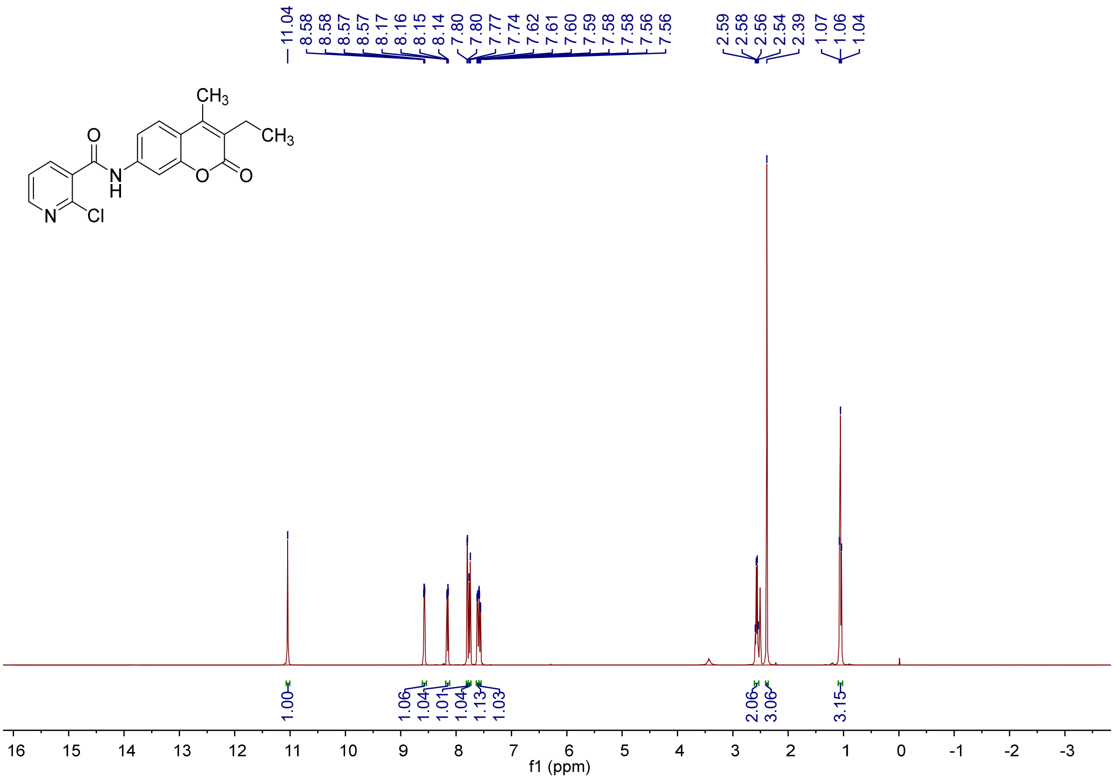

^{13}C NMR Spectrum of 5q (101 MHz, DMSO-d_6)

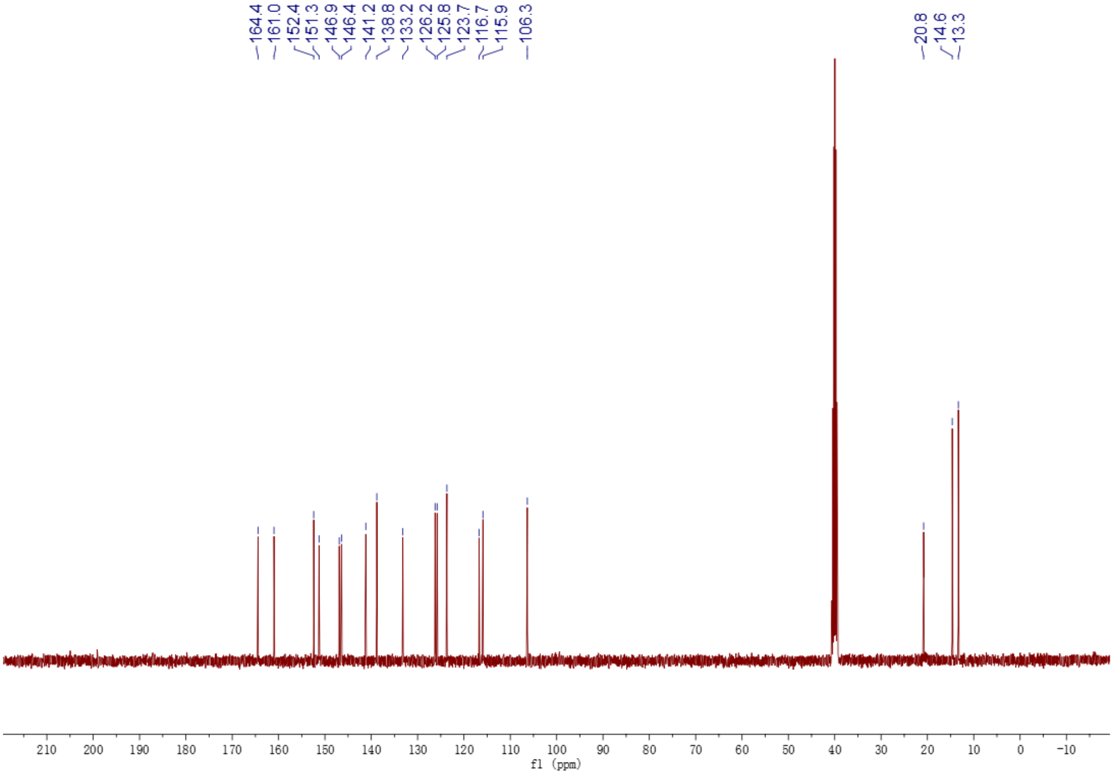

HRMS Spectrum of 5q

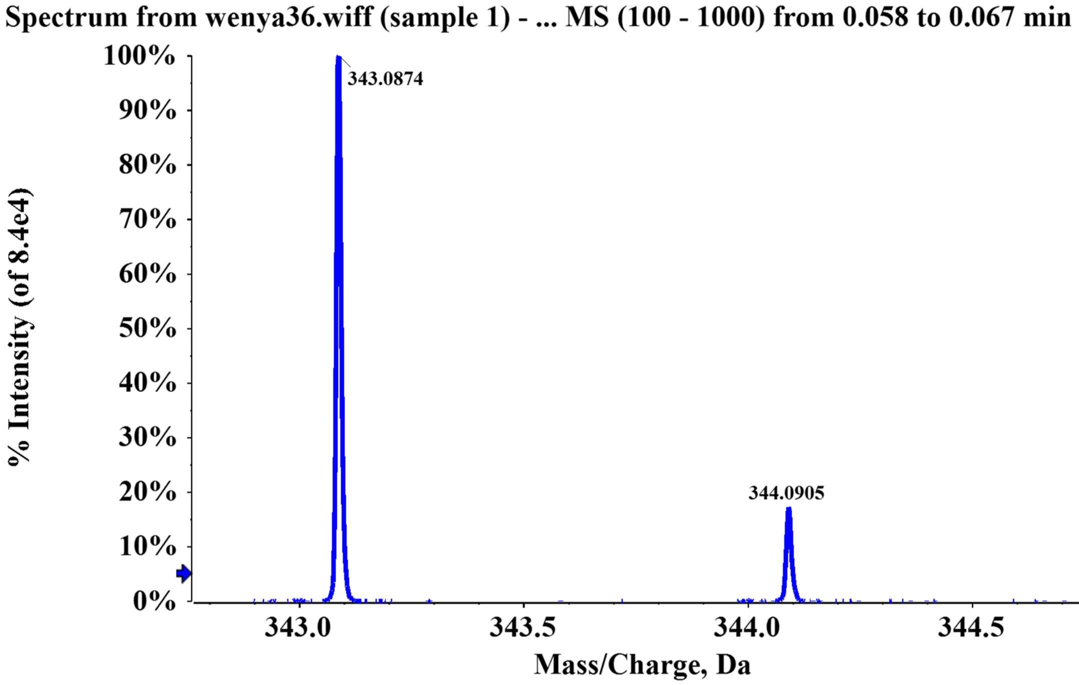

^{1}H NMR Spectrum of 5r (400 MHz, DMSO-d_6)

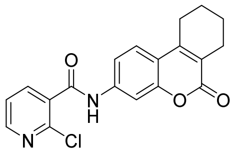

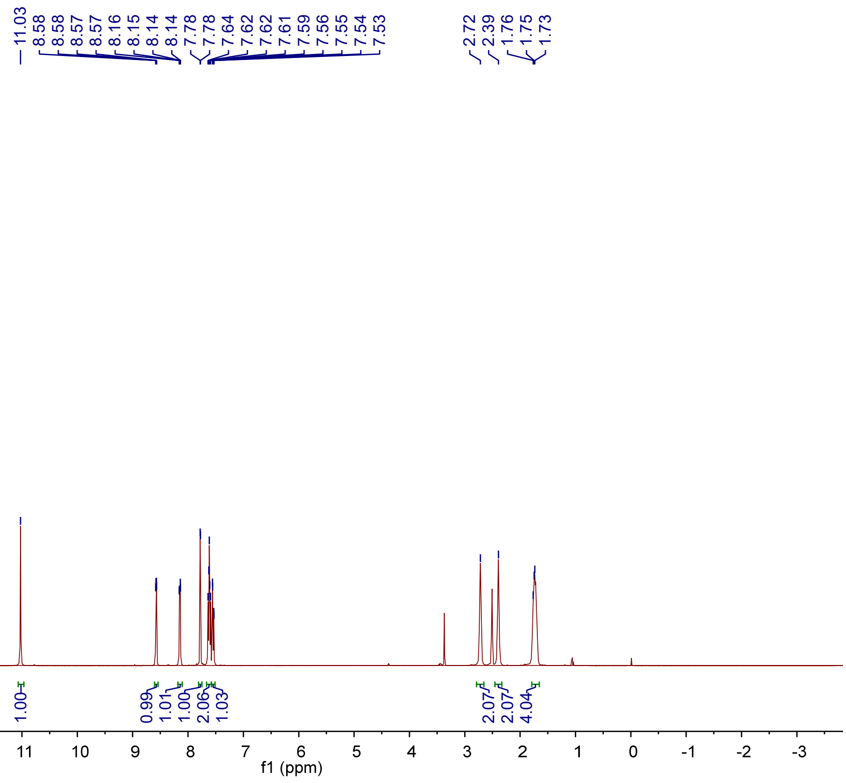

^{13}C NMR Spectrum of 5r (101 MHz, DMSO-d_6)

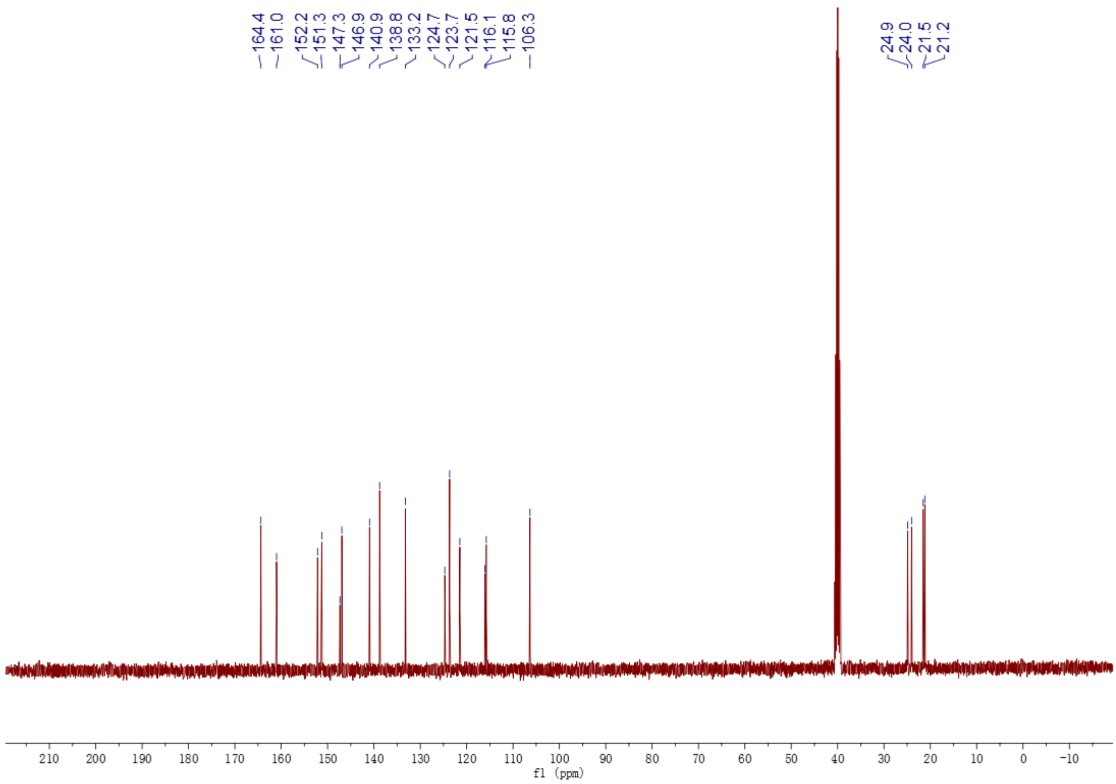

HRMS Spectrum of 5r

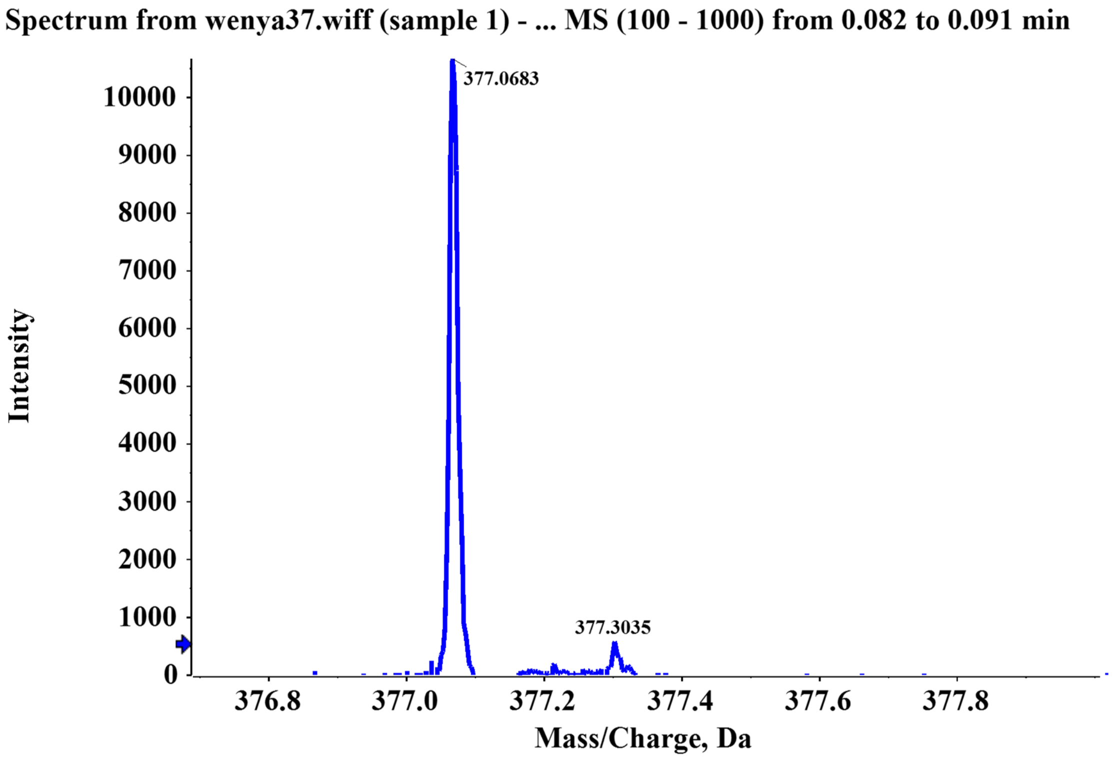

^{1}H NMR Spectrum of 5s (400 MHz, DMSO-d_6)

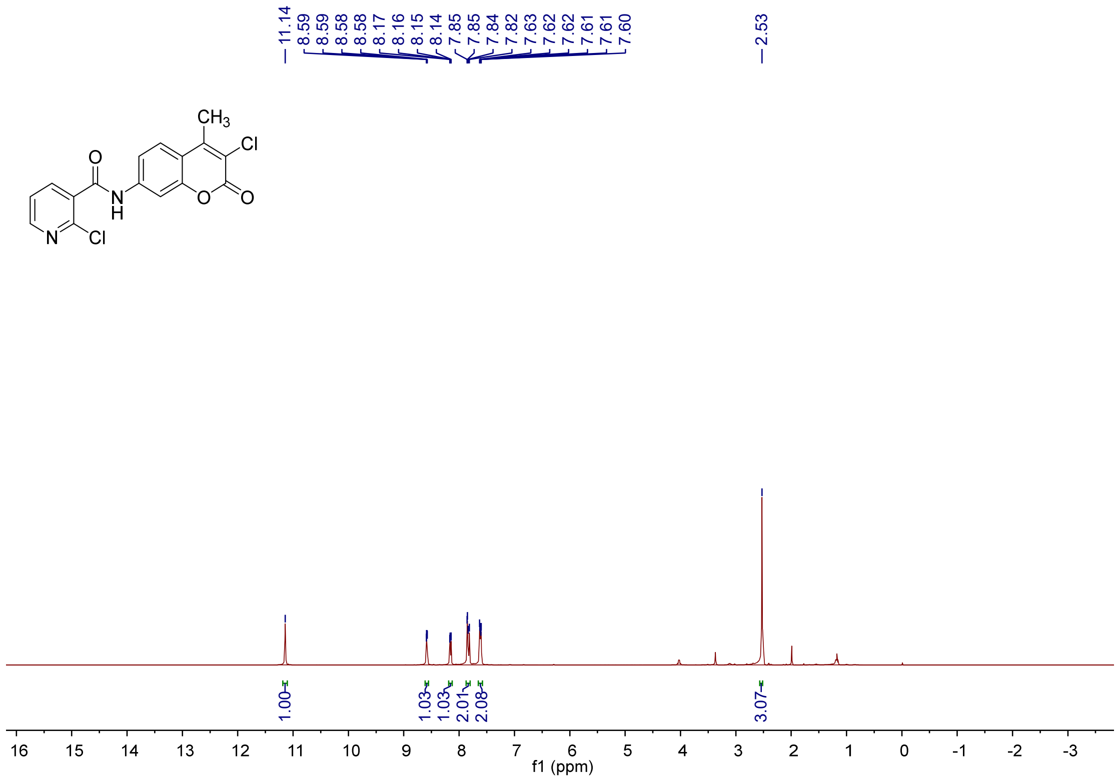

^{13}C NMR Spectrum of 5s (101 MHz, DMSO-*d*$_6$)

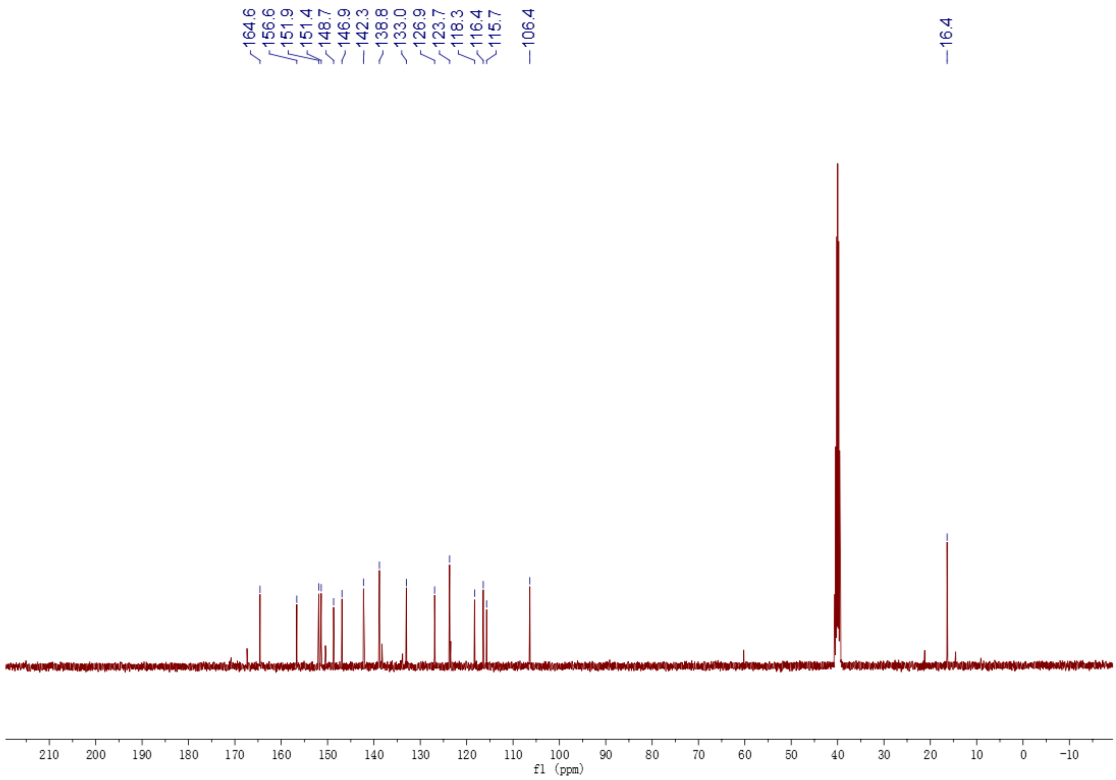

HRMS Spectrum of 5s

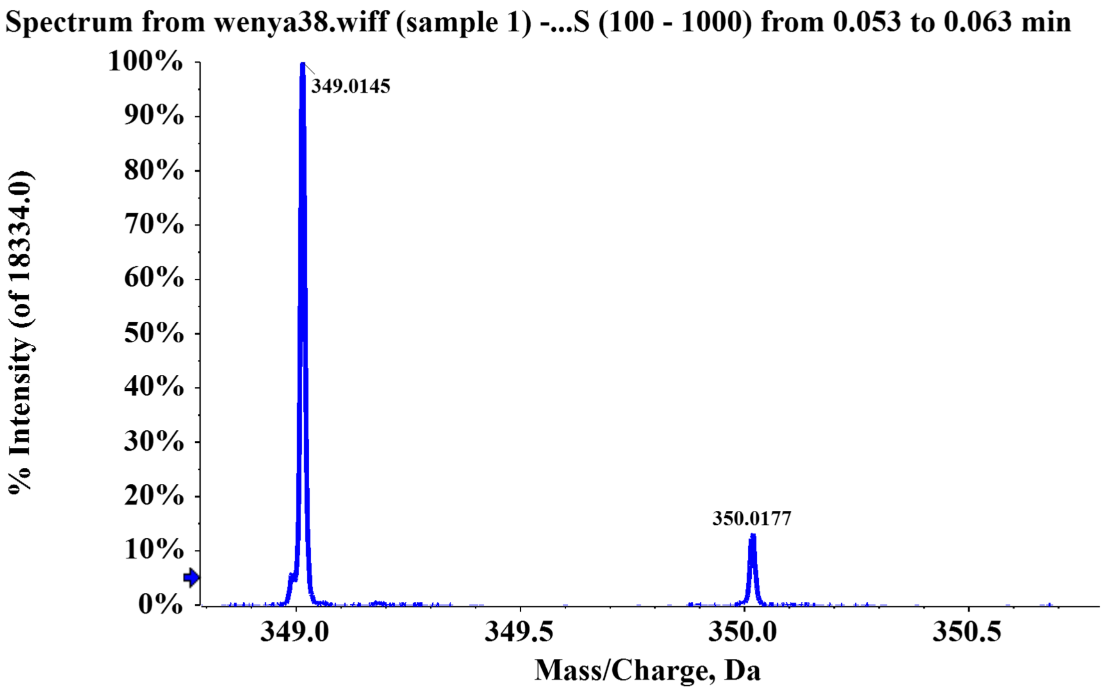

^{1}H NMR Spectrum of 5t (400 MHz, DMSO-d_6)

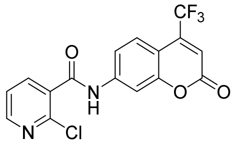

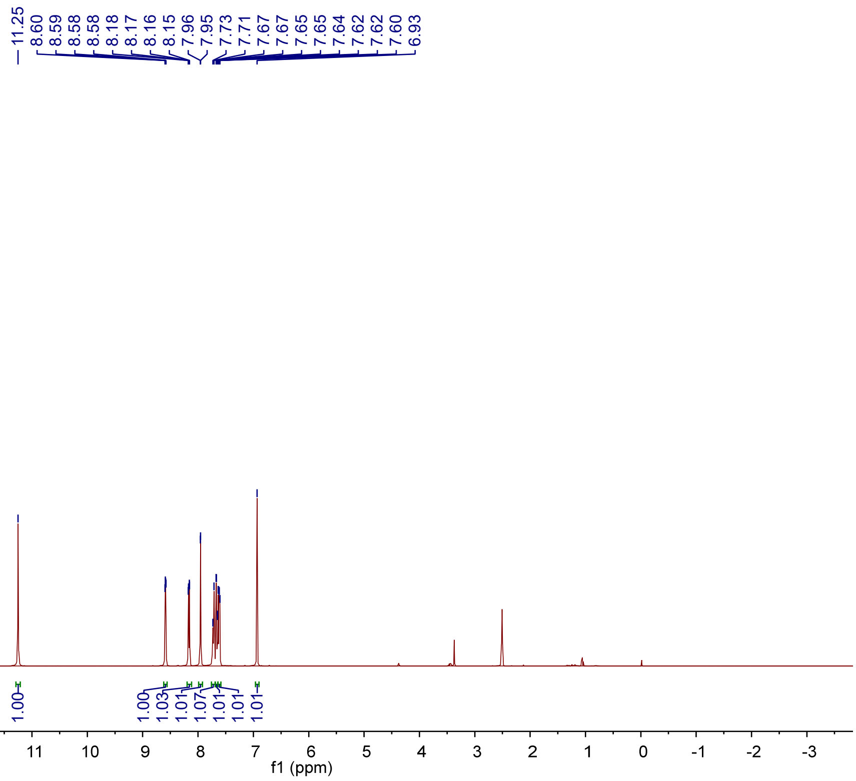

^{13}C NMR Spectrum of 5t (101 MHz, DMSO-d_6)

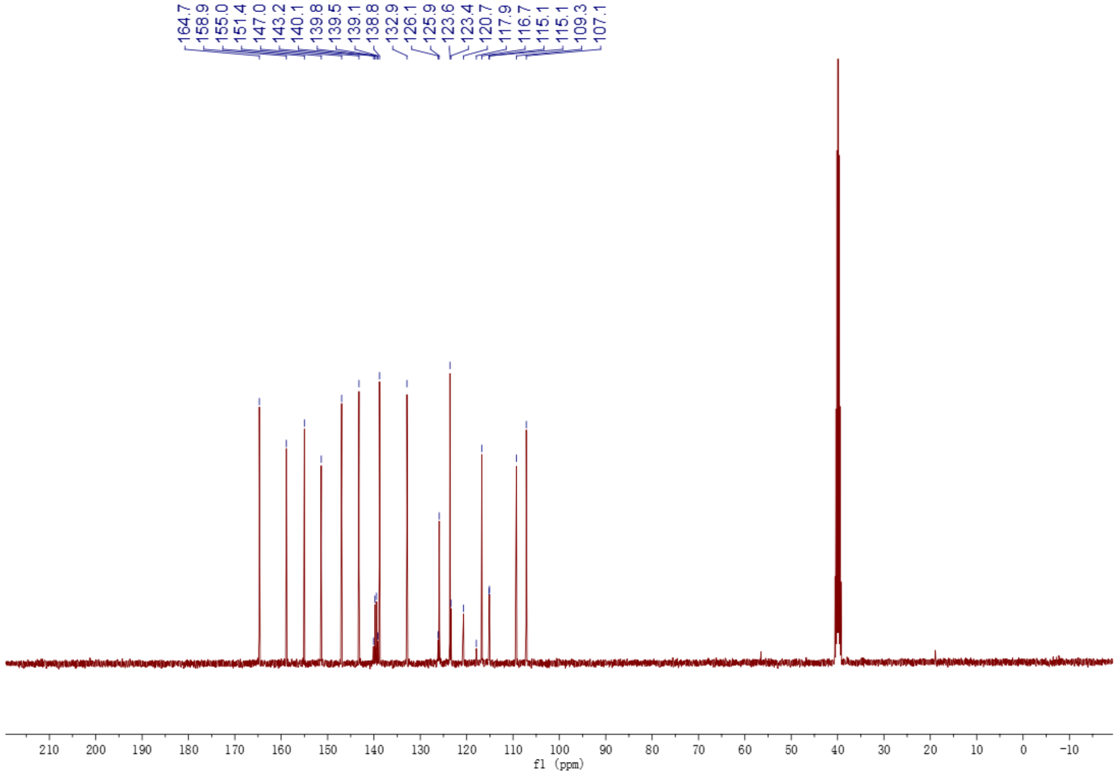

HRMS Spectrum of 5t

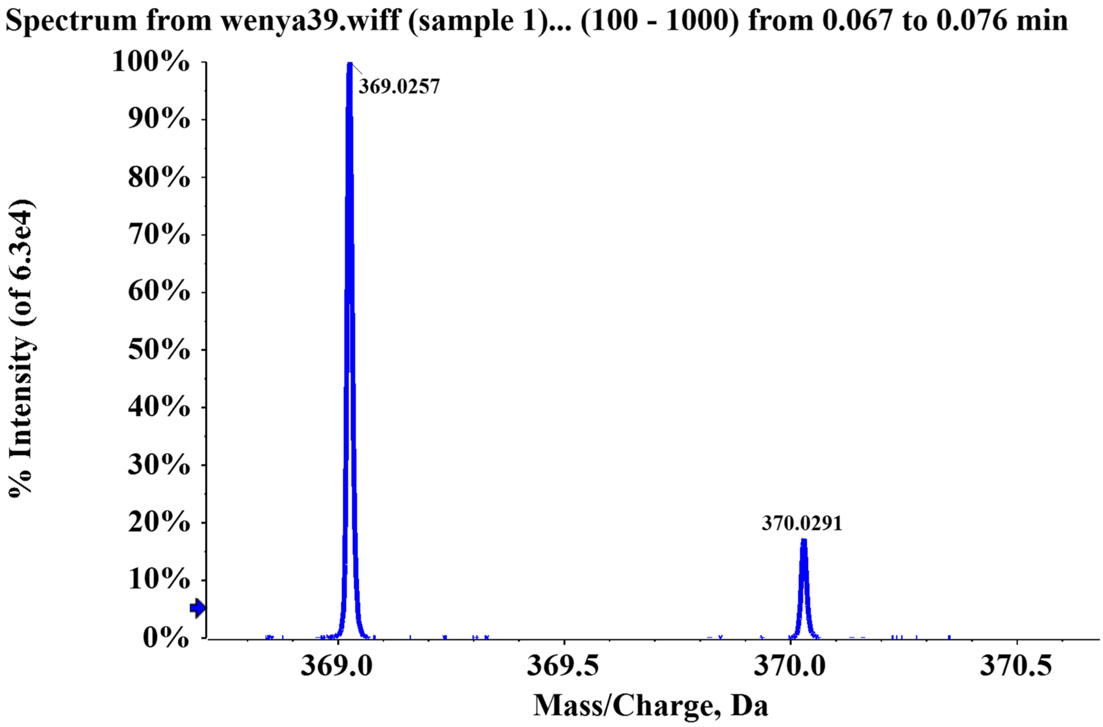

^{1}H NMR Spectrum of 6a (400 MHz, DMSO-d_6)

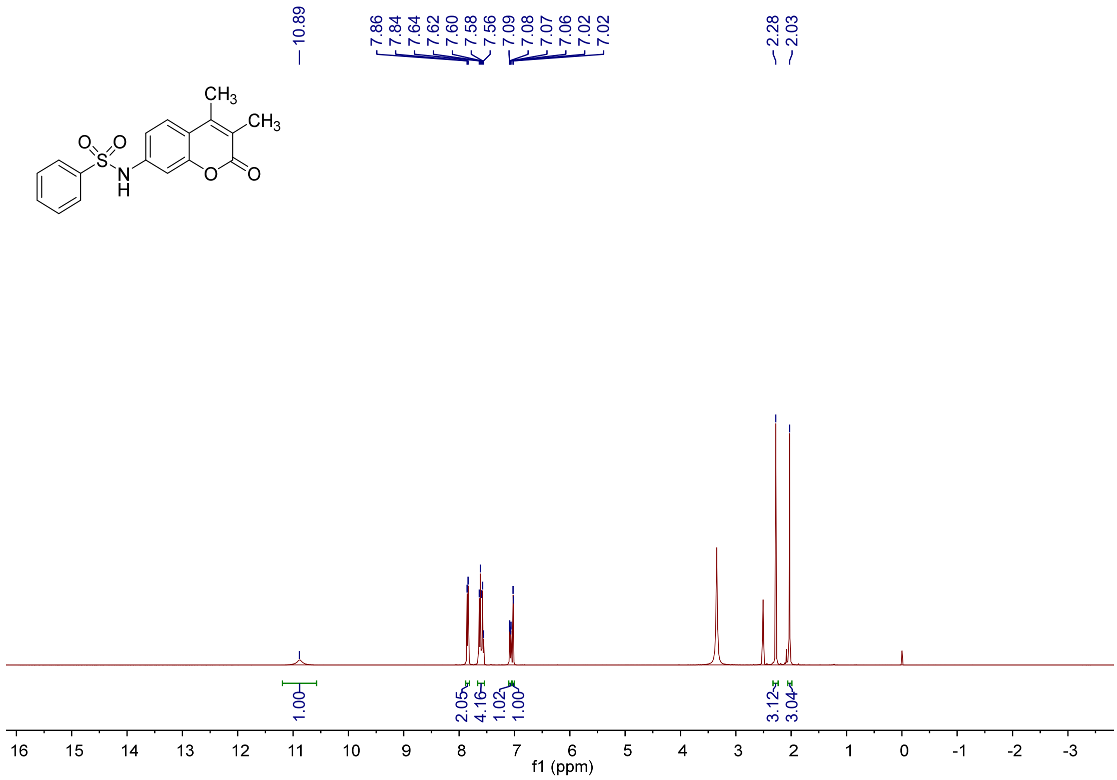

^{13}C NMR Spectrum of 6a (101 MHz, DMSO-*d*$_6$)

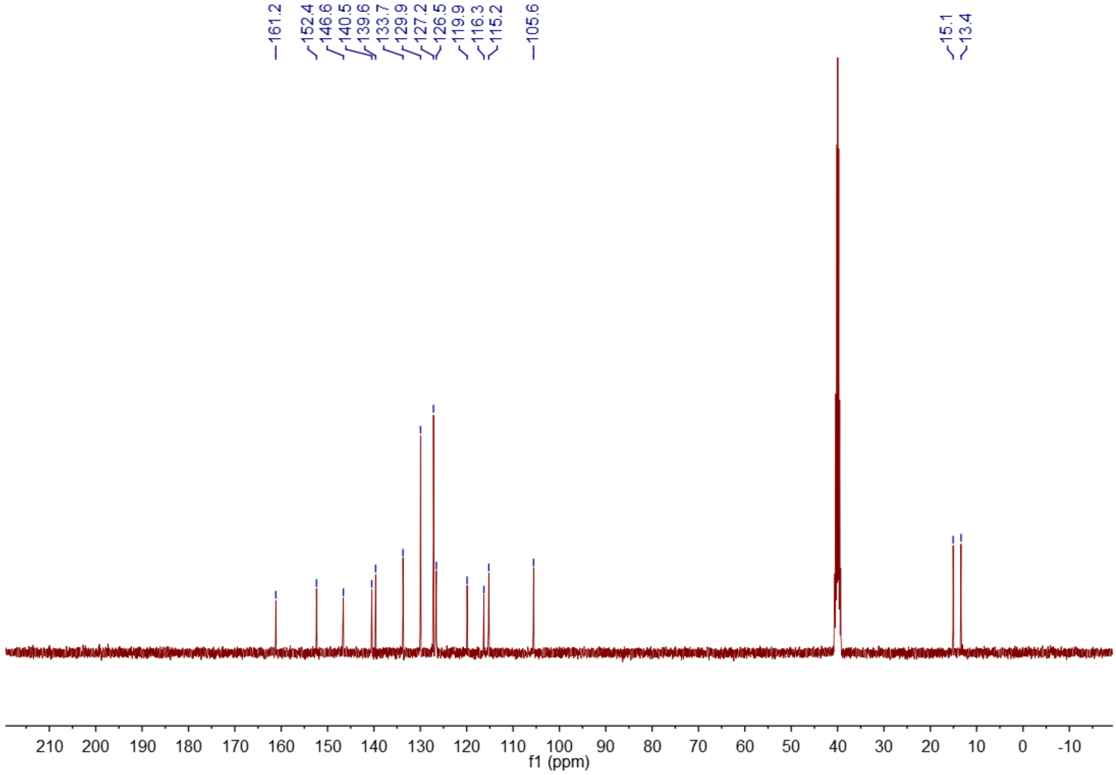

HRMS Spectrum of 6a

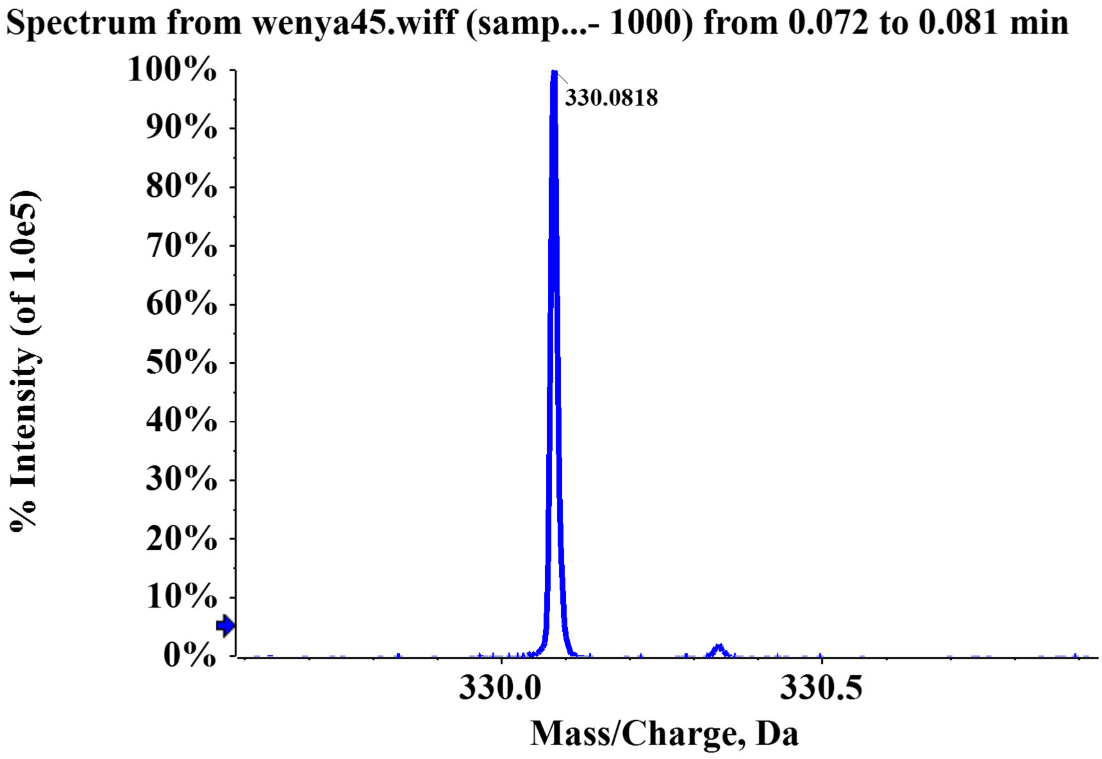

^{1}H NMR Spectrum of 6b (400 MHz, DMSO-d_6)

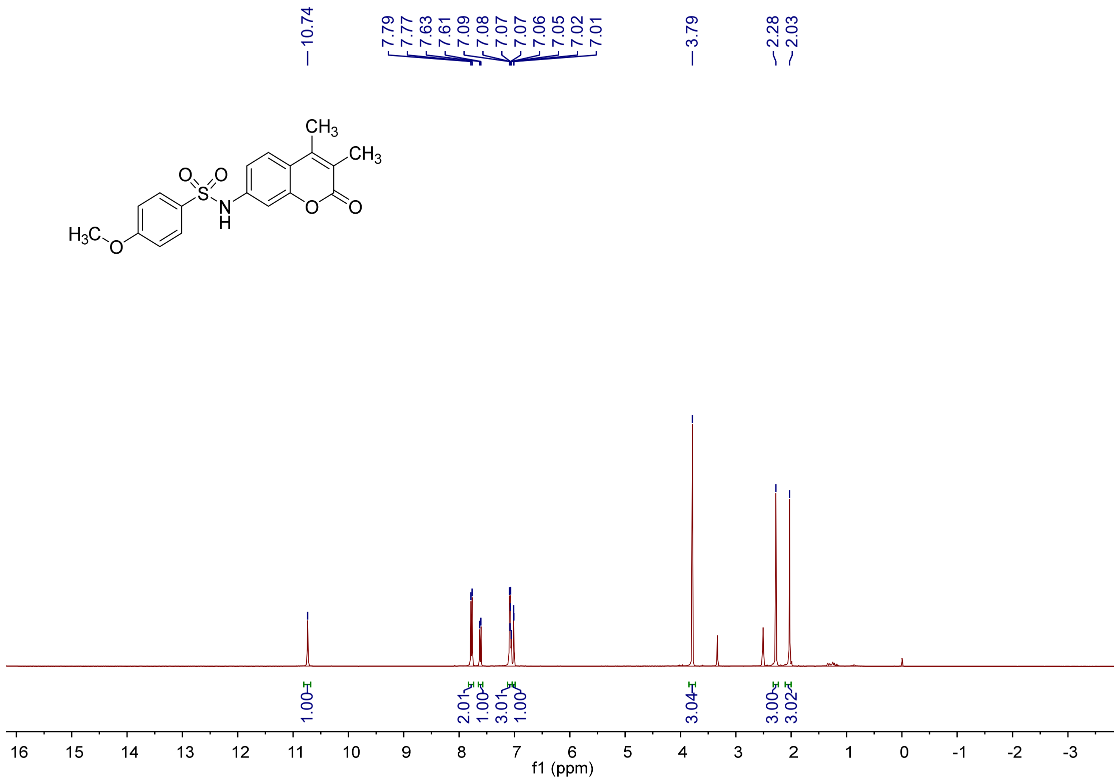

^{13}C NMR Spectrum of 6b (101 MHz, DMSO-d_6)

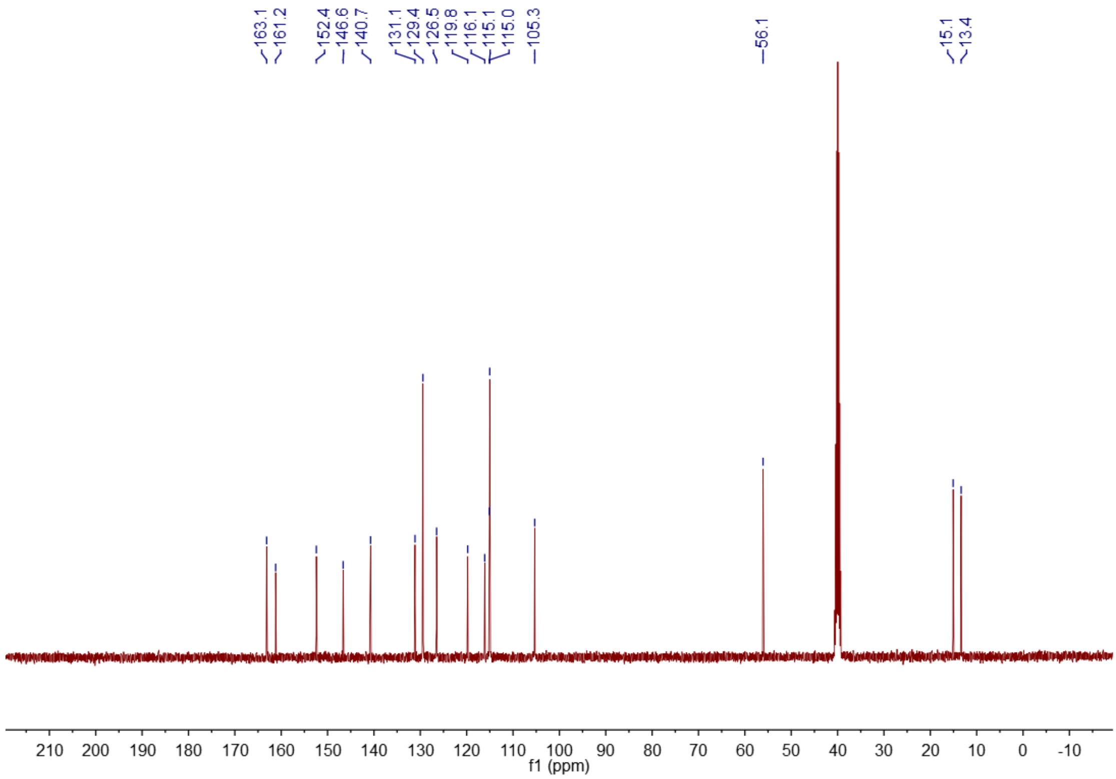

HRMS Spectrum of 6b

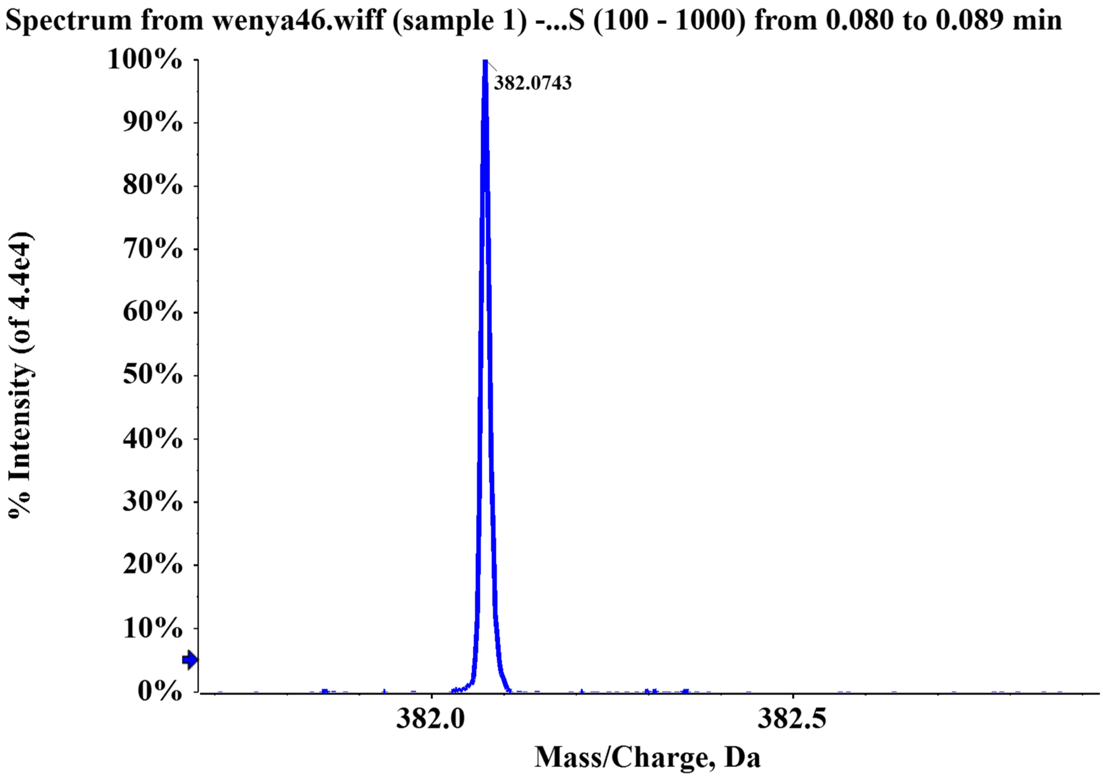

^{1}H NMR Spectrum of 6c (400 MHz, DMSO-d_6)

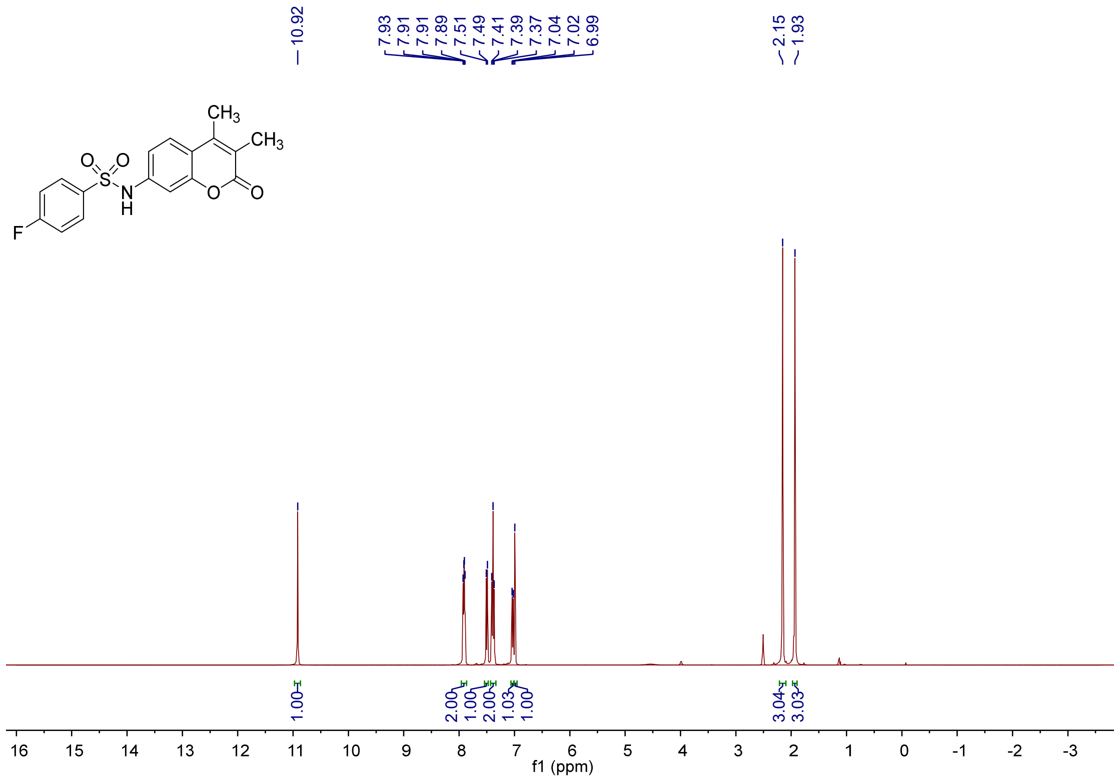

^{13}C NMR Spectrum of 6c (101 MHz, DMSO-d_6)

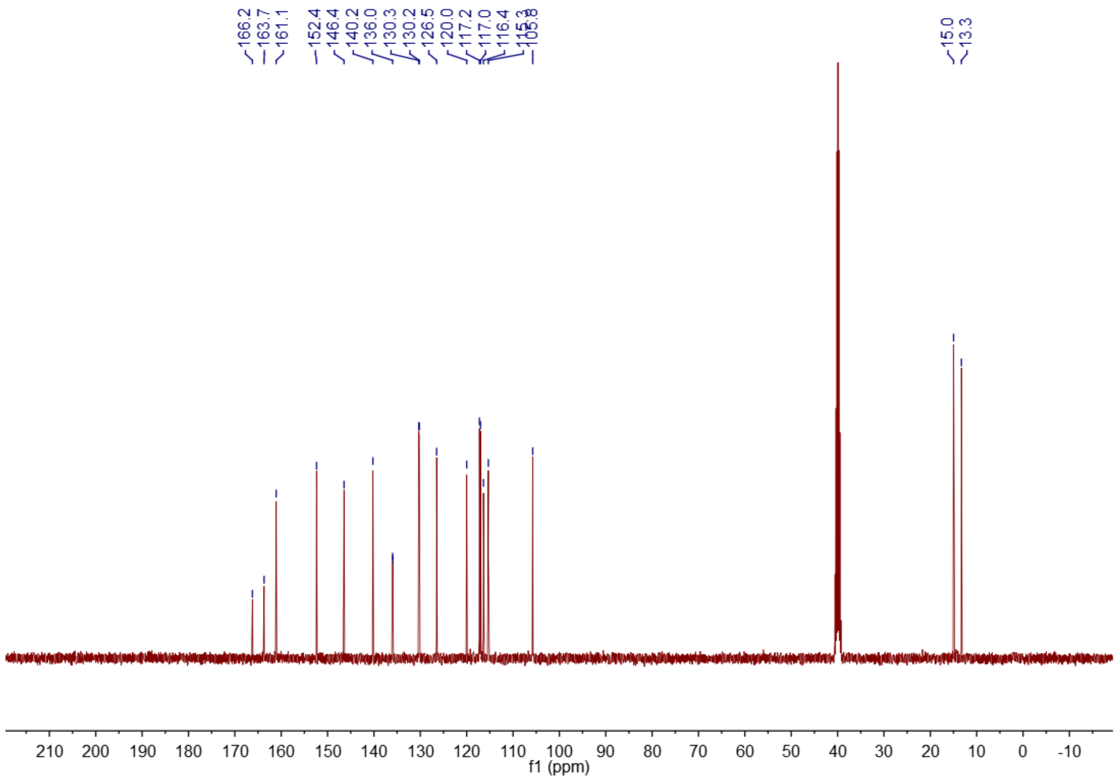

HRMS Spectrum of 6c

Spectrum from wenya47.wiff (sample 1) - w... MS (100 - 1000) from 0.084 to 0.093 min

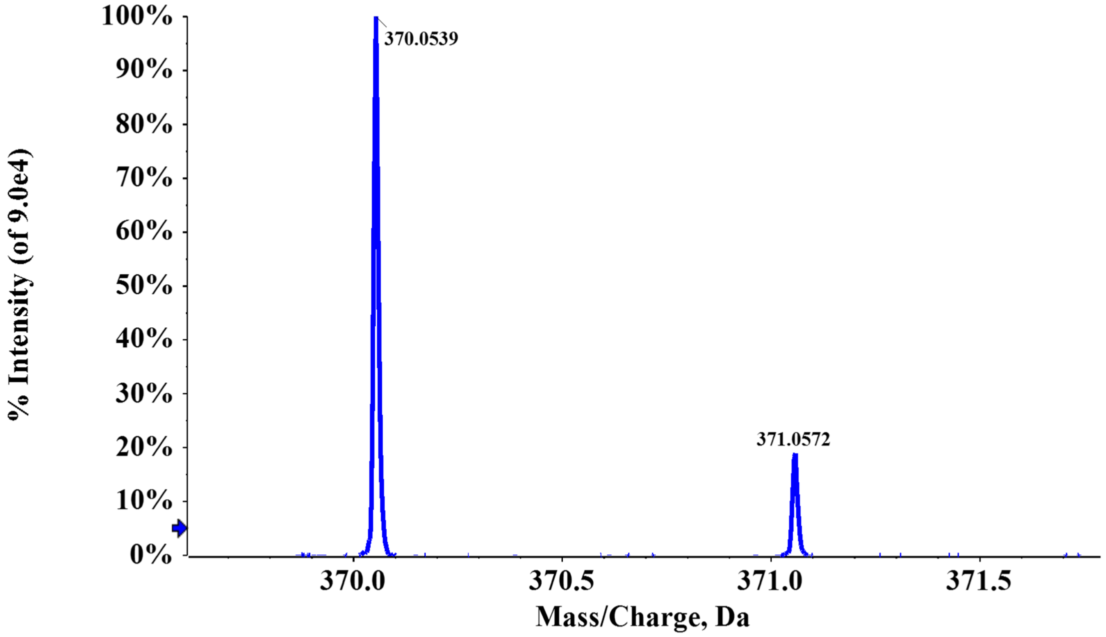

^{1}H NMR Spectrum of 6d (400 MHz, DMSO-d_6)

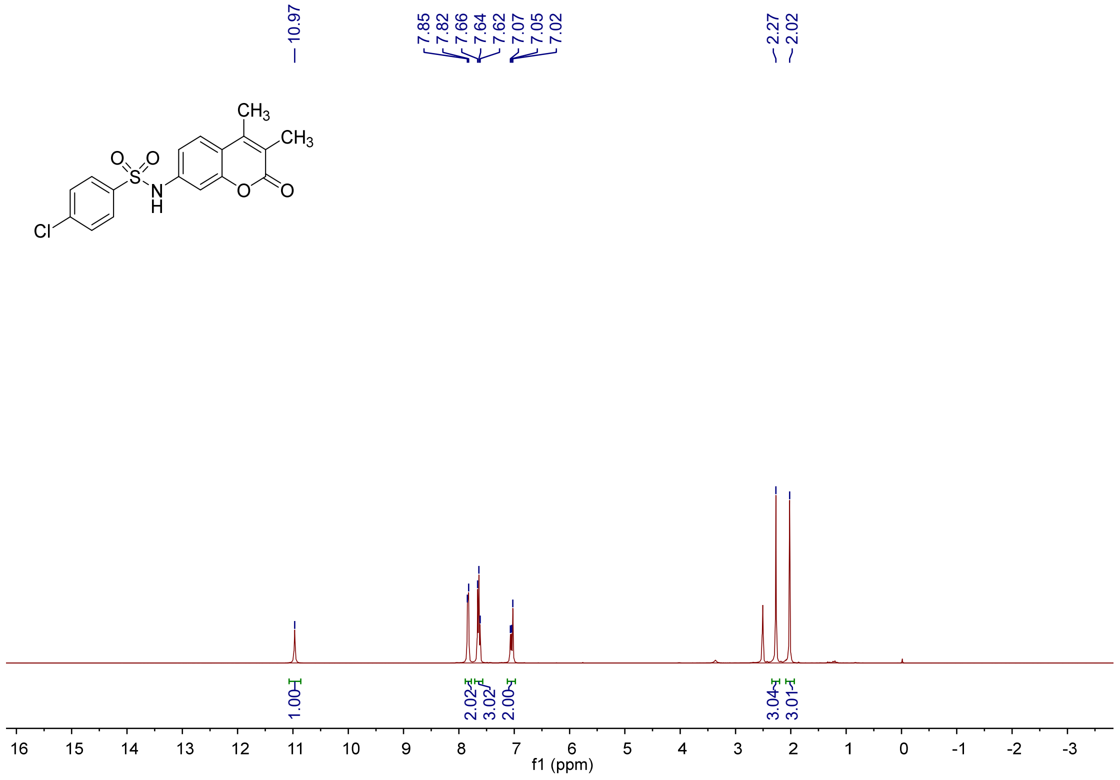

^{13}C NMR Spectrum of 6d (101 MHz, DMSO-d_6)

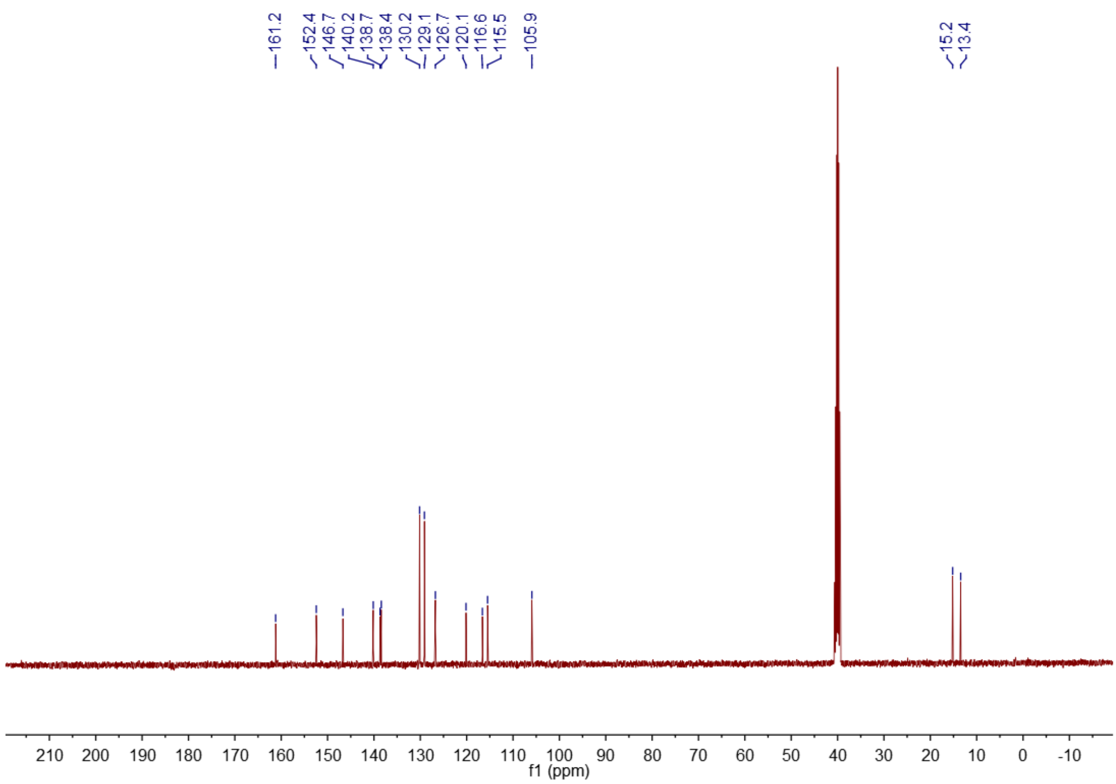

HRMS Spectrum of 6d

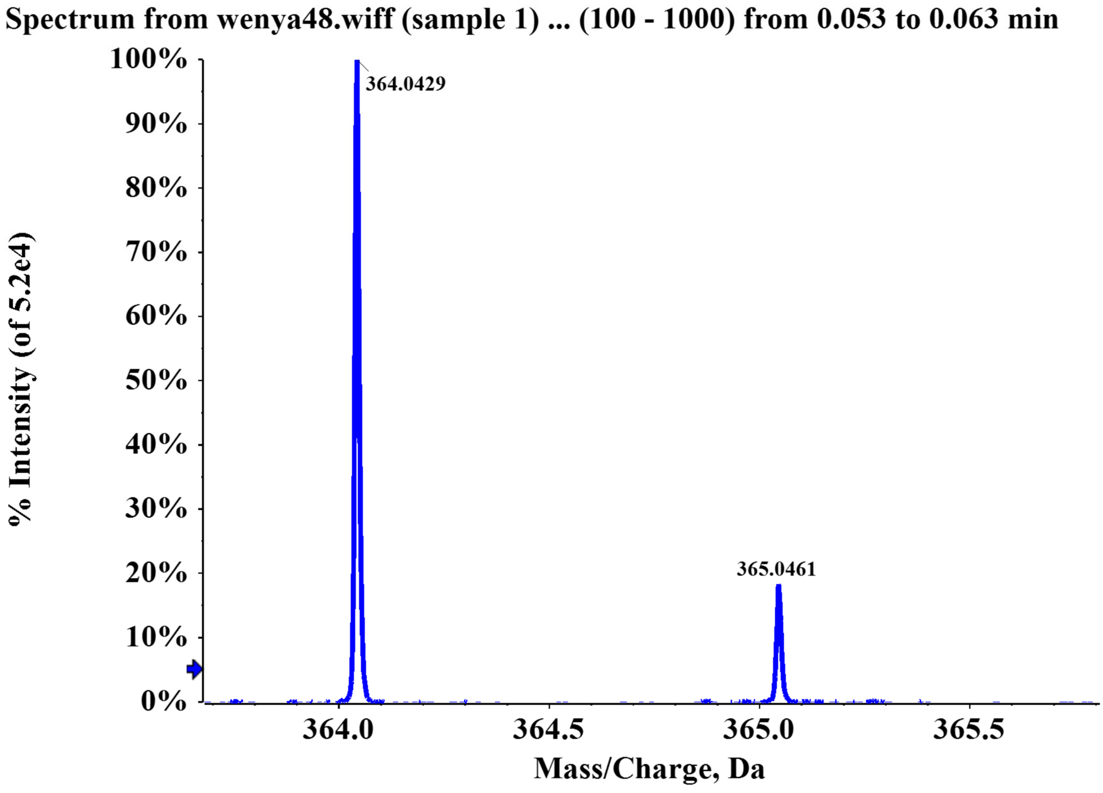

^{1}H NMR Spectrum of 6e (400 MHz, DMSO-d_6)

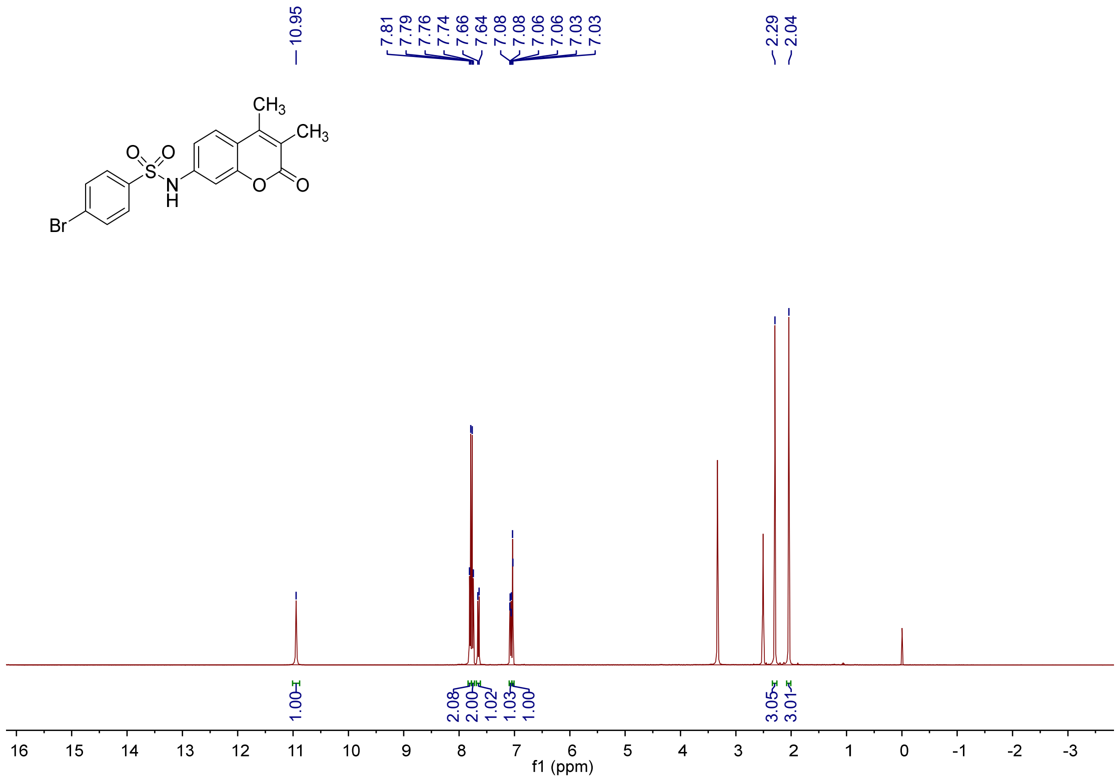

^{13}C NMR Spectrum of 6e (101 MHz, DMSO-d_6)

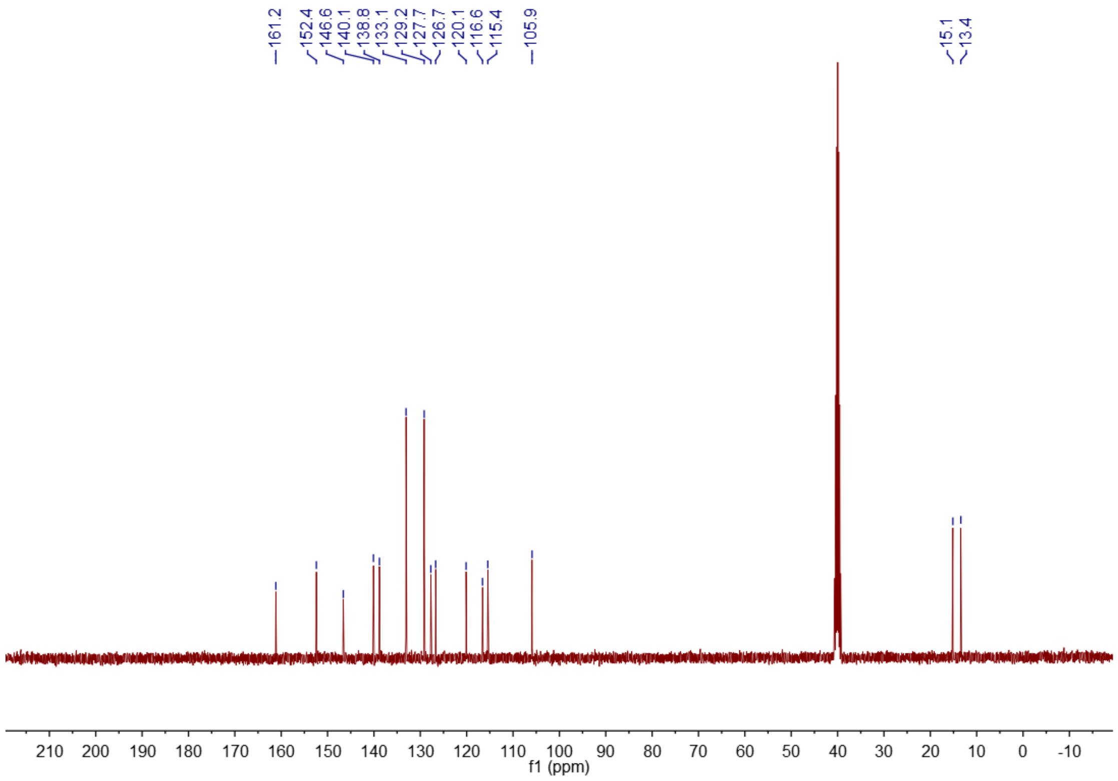

HRMS Spectrum of 6e

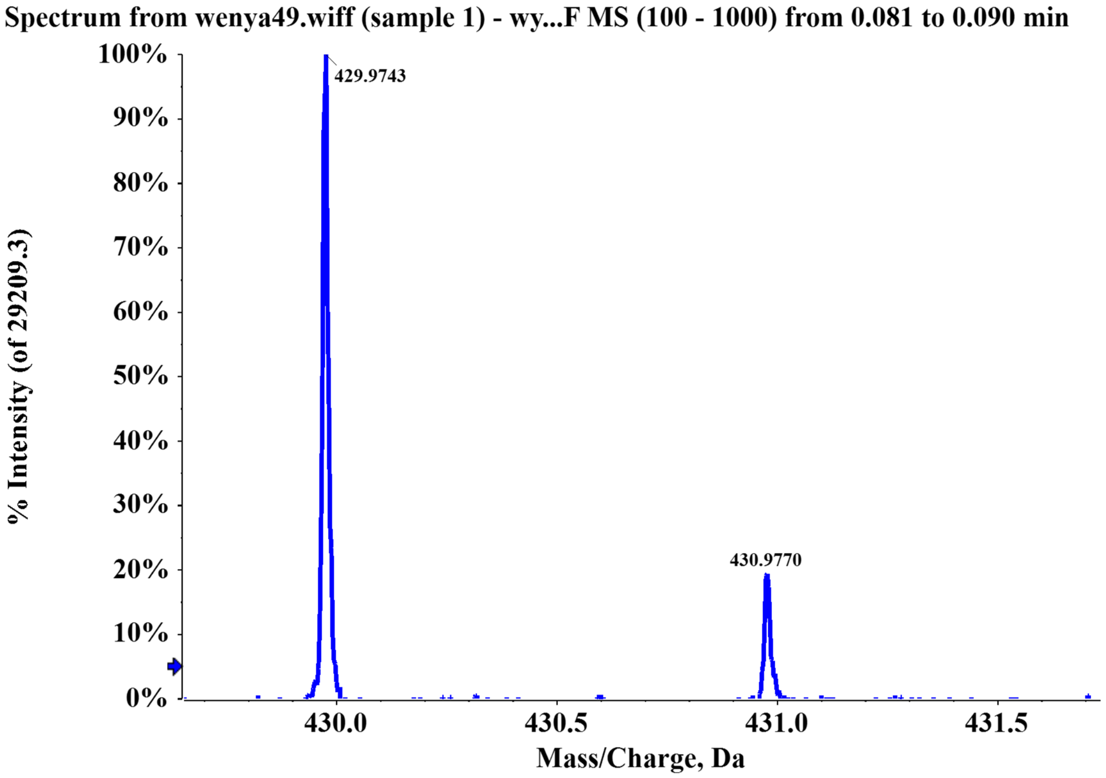

^{1}H NMR Spectrum of 6f (400 MHz, DMSO-d_6)

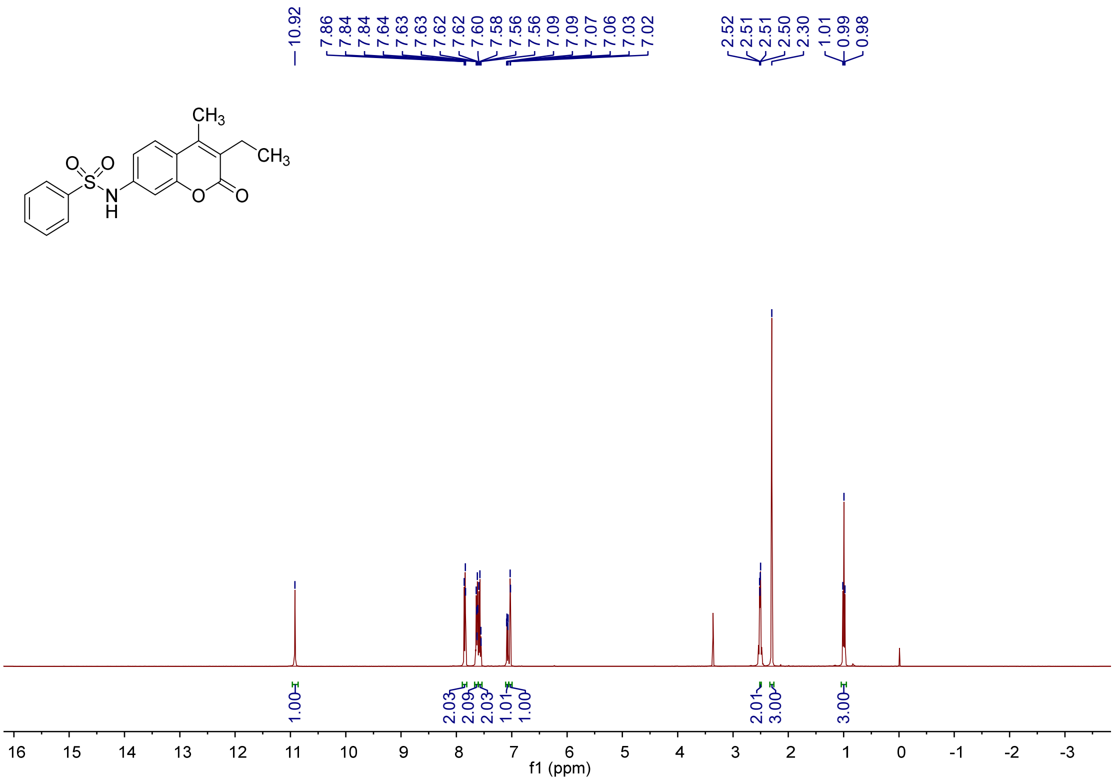

^{13}C NMR Spectrum of 6f (101 MHz, DMSO-d_6)

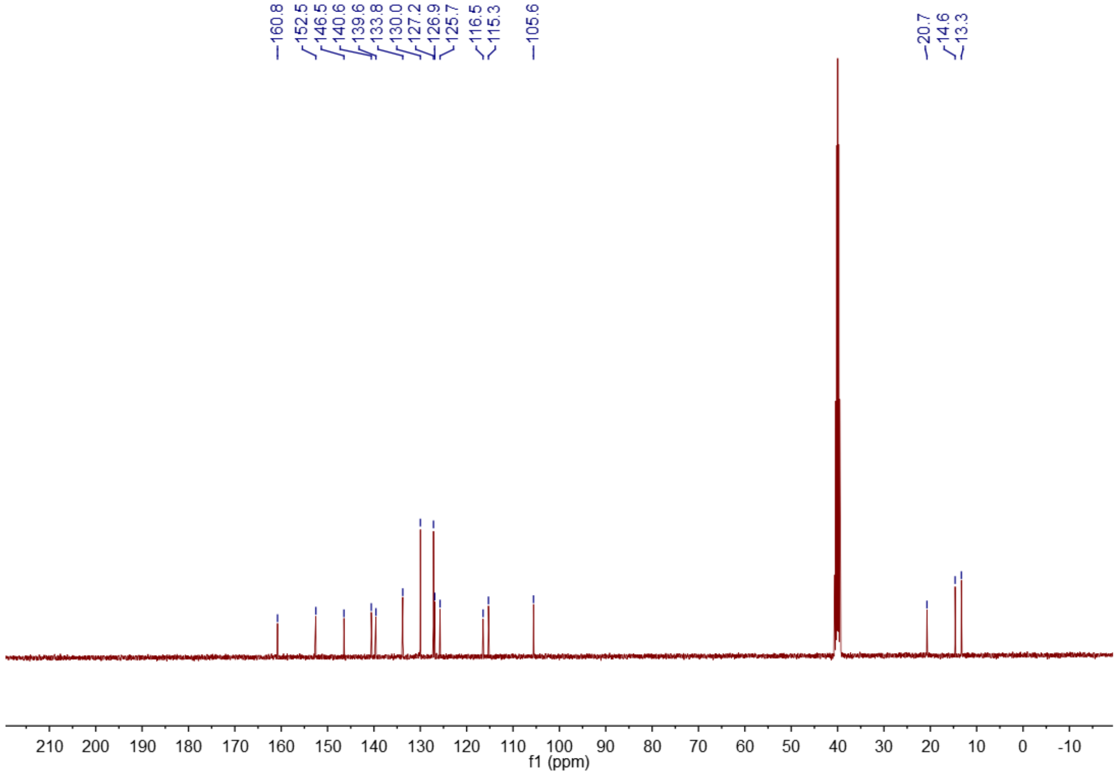

HRMS Spectrum of 6f

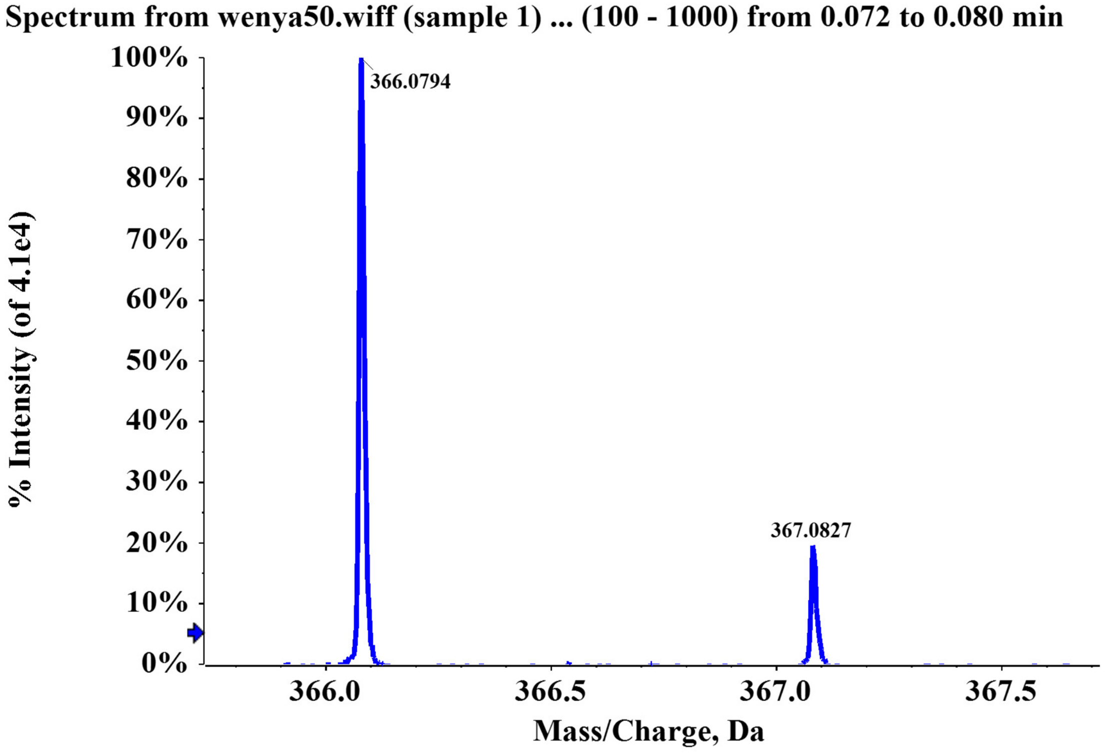

^{1}H NMR Spectrum of 6g (400 MHz, DMSO-d_6)

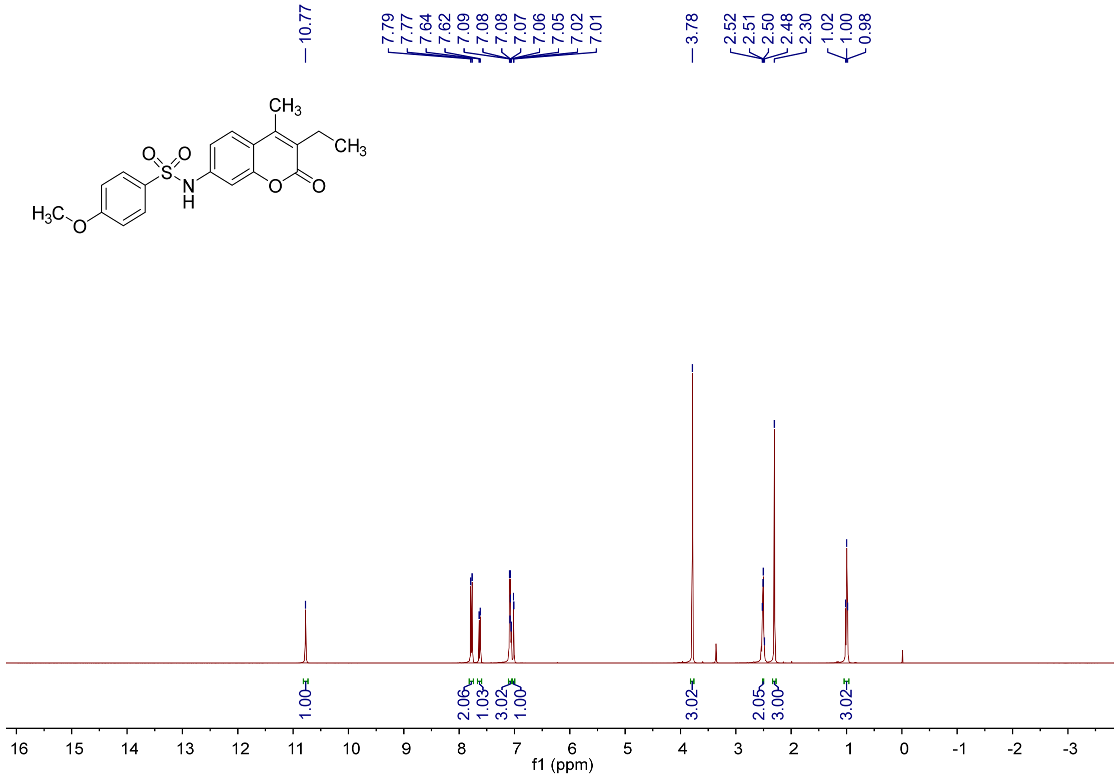

^{13}C NMR Spectrum of 6g (101 MHz, DMSO-d_6)

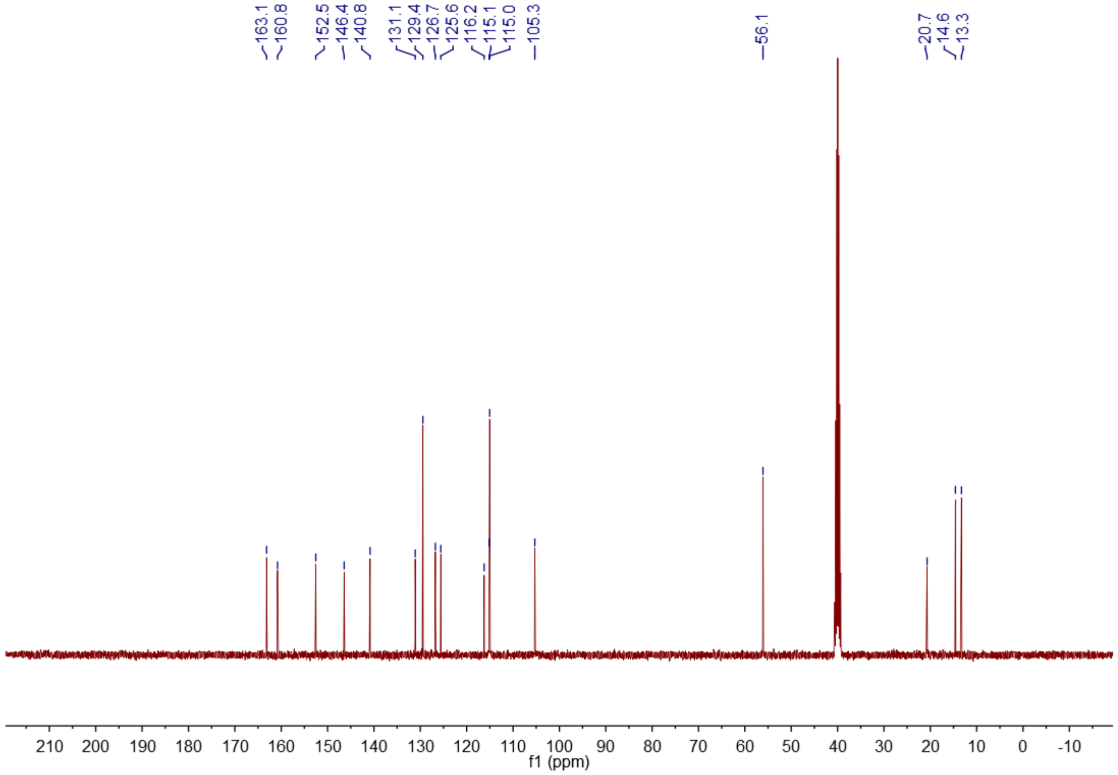

HRMS Spectrum of 6g

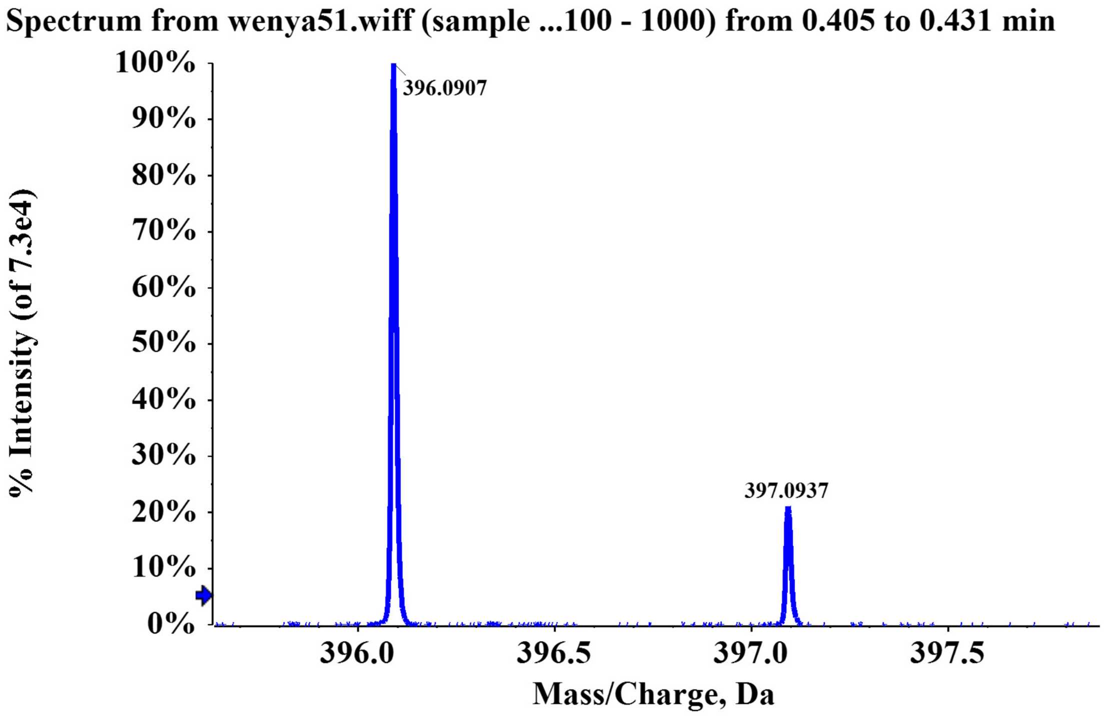

^{1}H NMR Spectrum of 6h (400 MHz, DMSO-d_6)

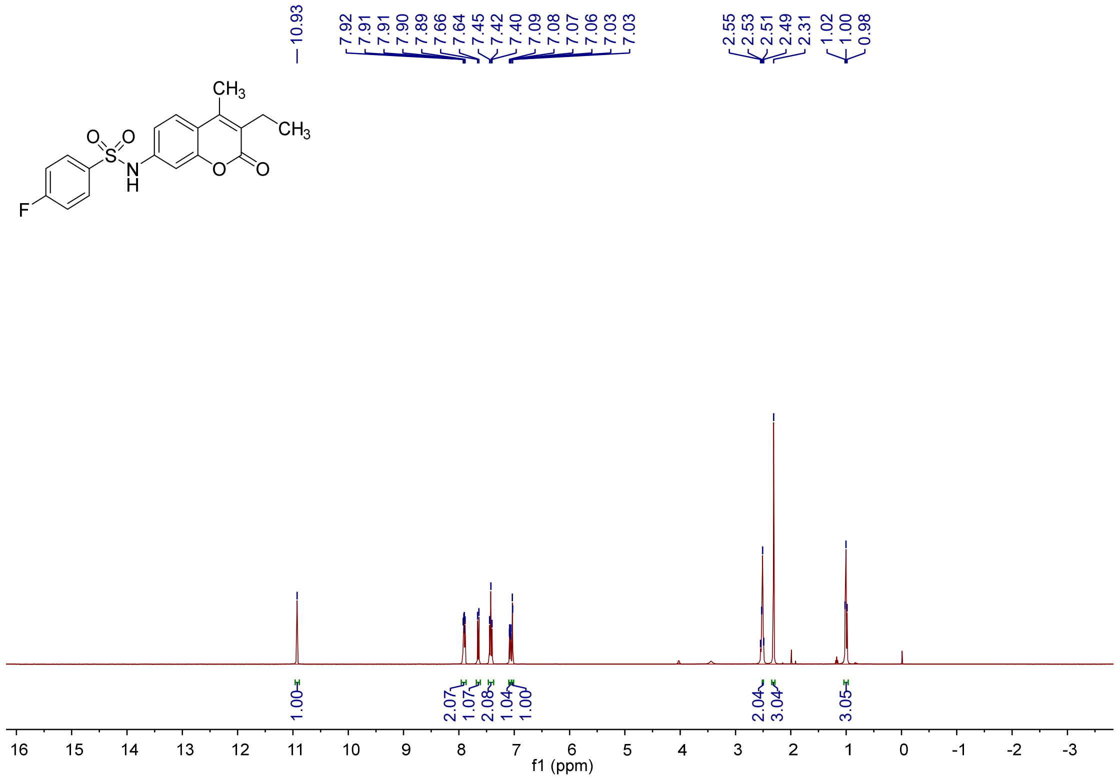

^{13}C NMR Spectrum of 6h (101 MHz, DMSO-d_6)

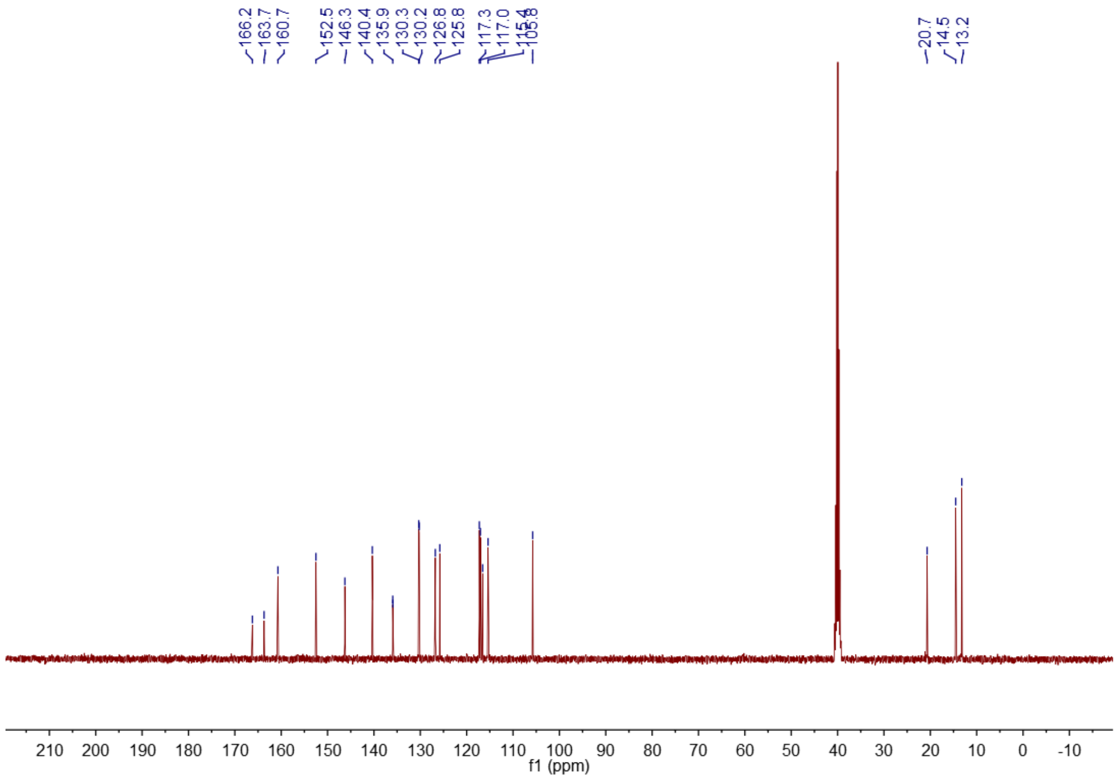

HRMS Spectrum of 6h

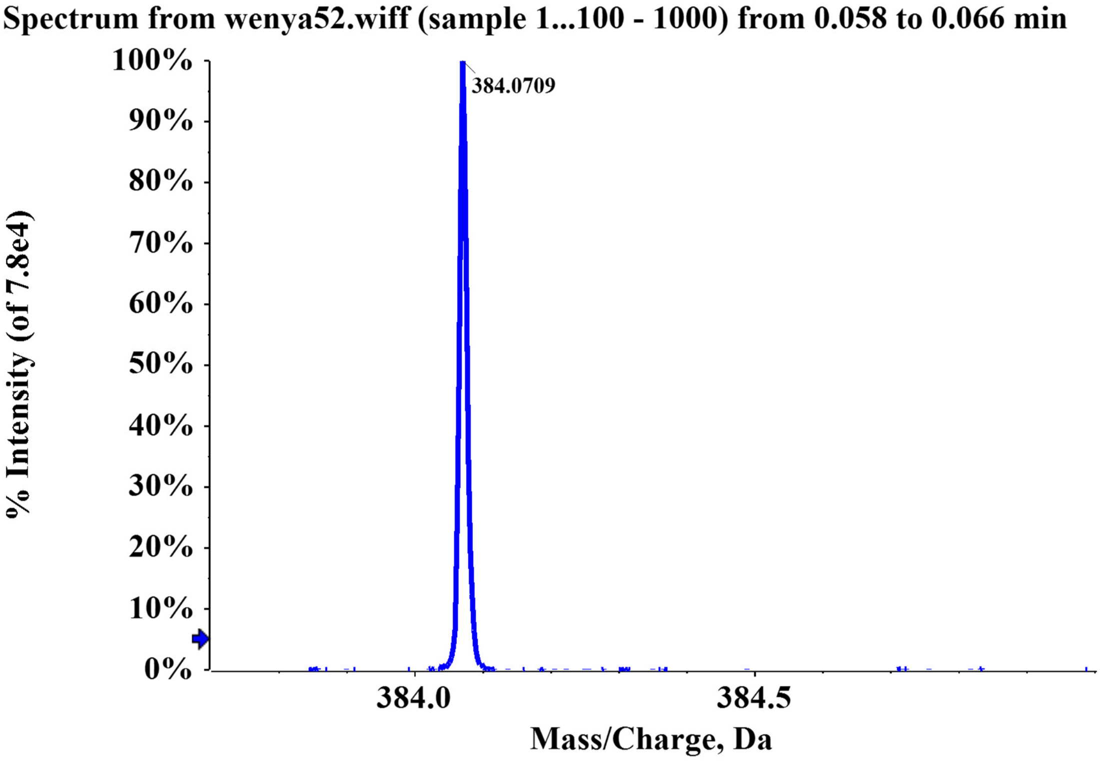

^{1}H NMR Spectrum of 6i (400 MHz, DMSO-d_6)

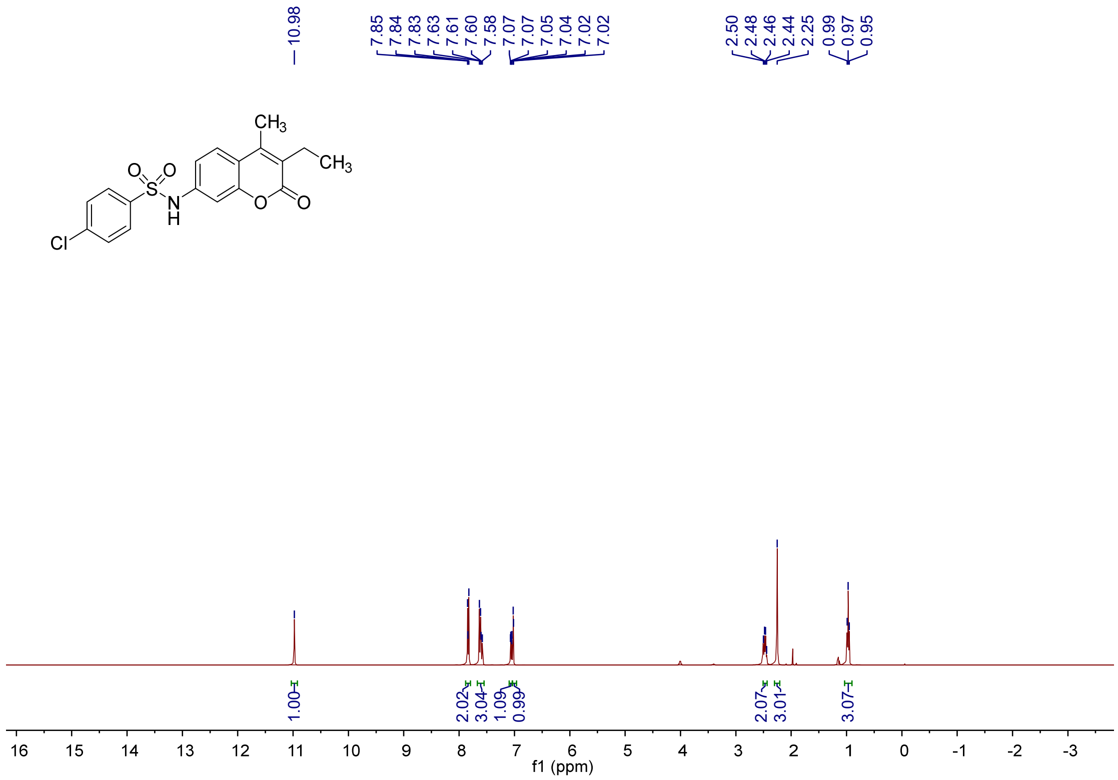

^{13}C NMR Spectrum of 6i (101 MHz, DMSO-d_6)

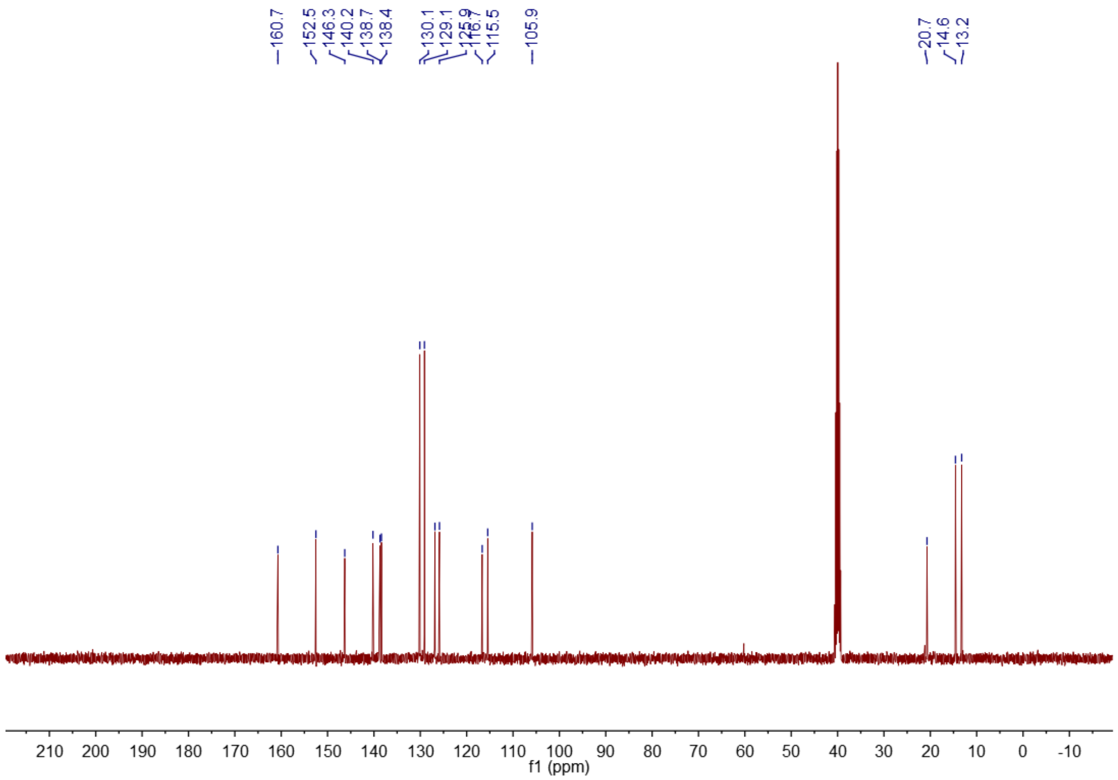

HRMS Spectrum of 6i

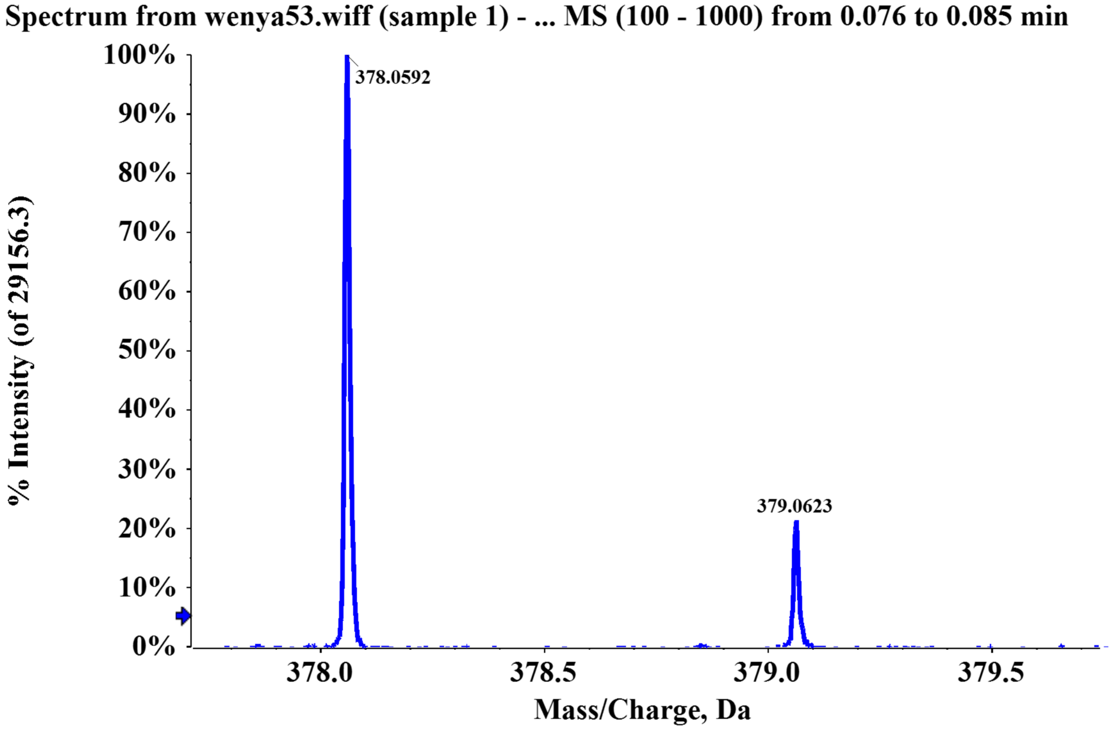

^{1}H NMR Spectrum of 6j (400 MHz, DMSO-d_6)

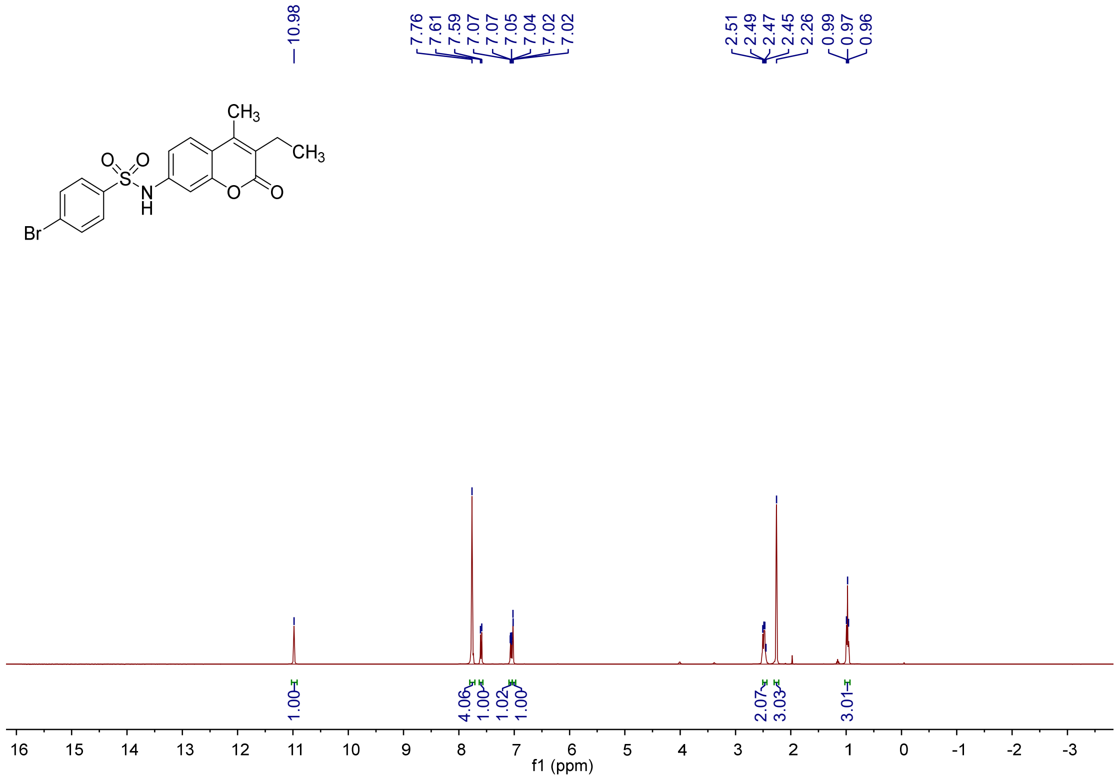

^{13}C NMR Spectrum of 6j (101 MHz, DMSO-d_6)

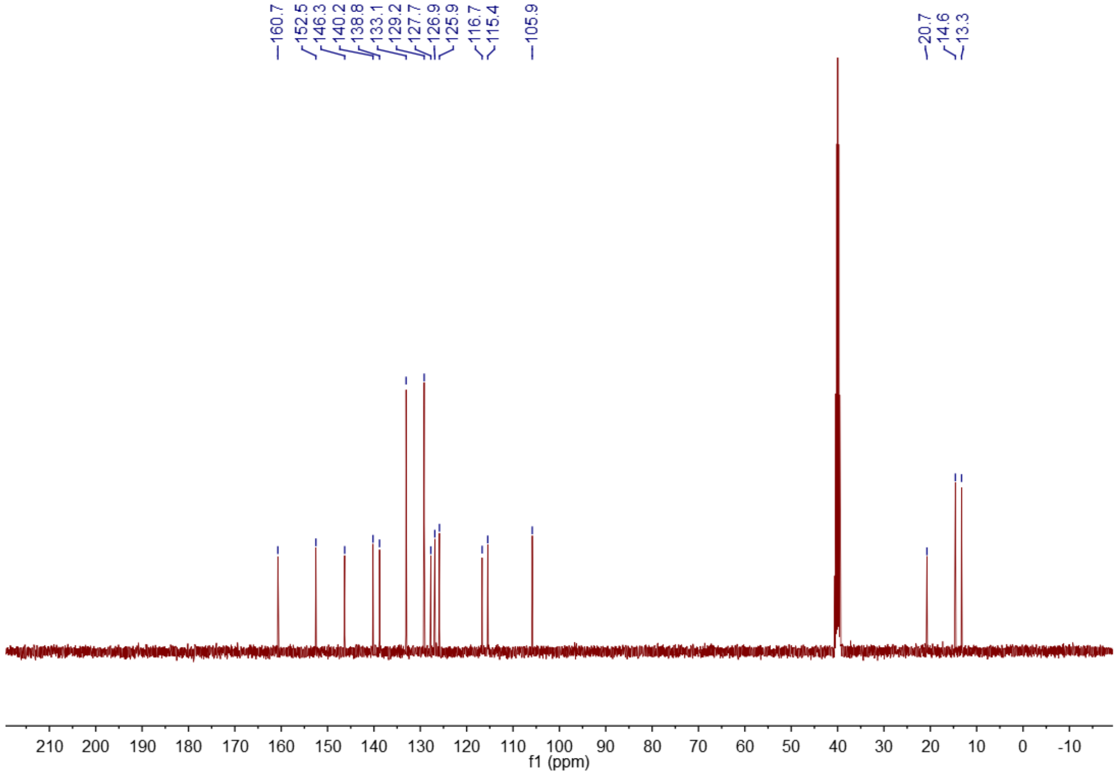

HRMS Spectrum of 6j

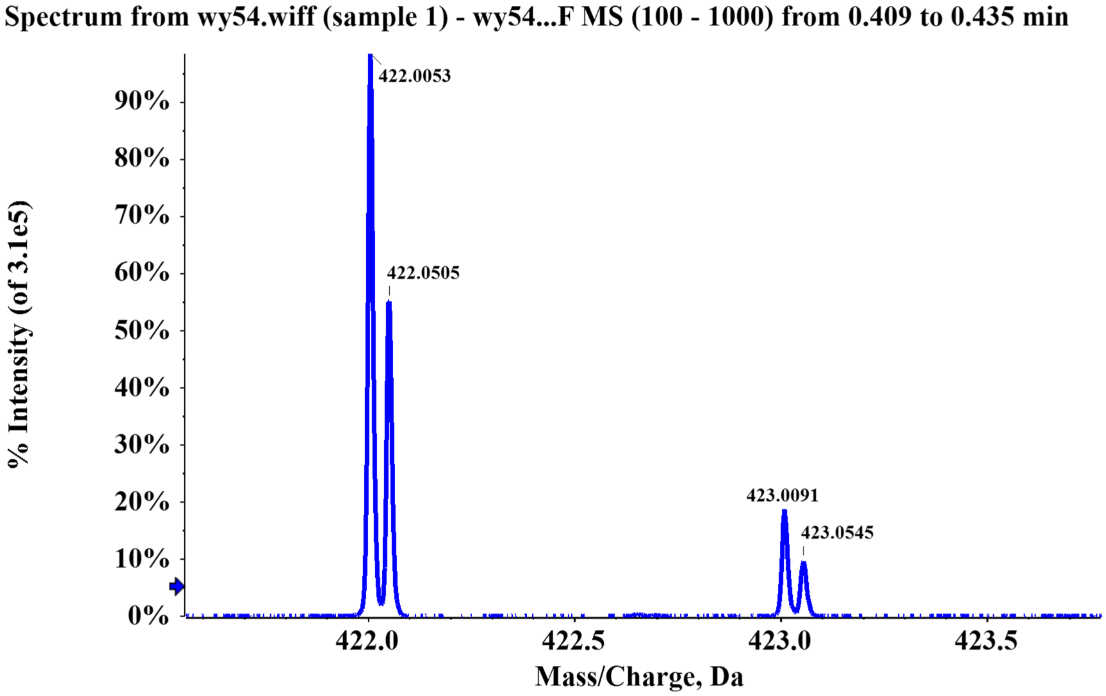

^{1}H **NMR Spectrum of 6k (400 MHz, DMSO-*d*$_6$)**

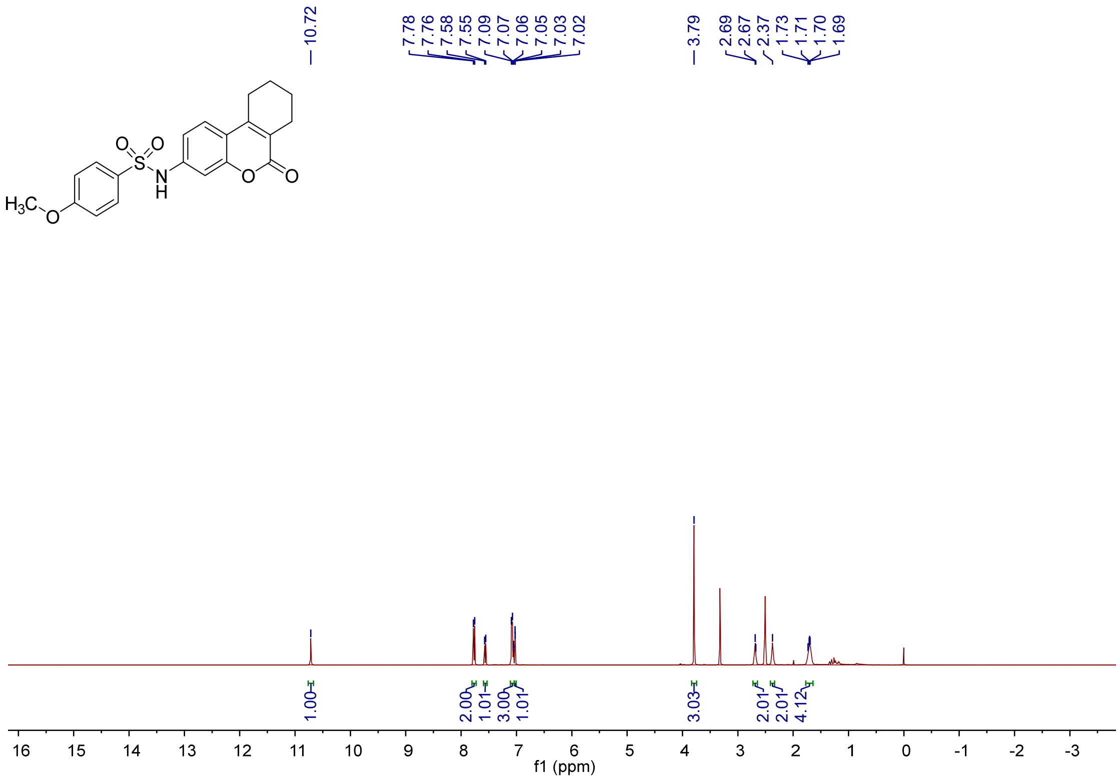

^{13}C NMR Spectrum of 6k (101 MHz, DMSO-*d*$_6$)

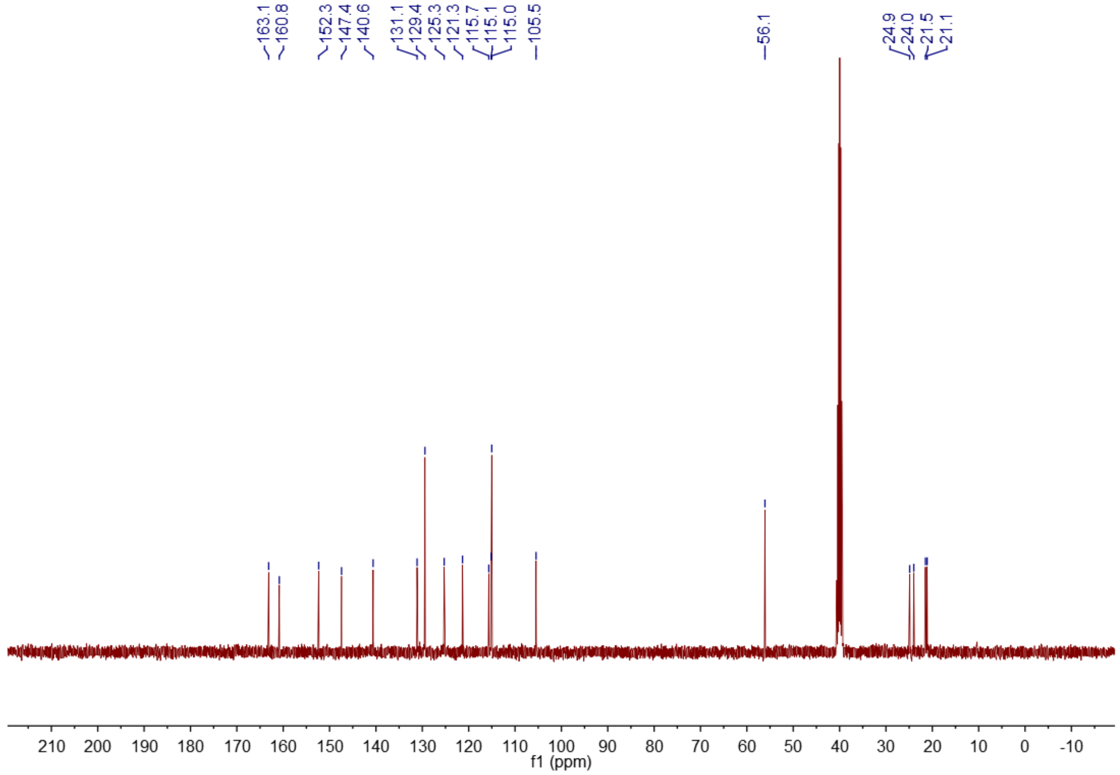

HRMS Spectrum of 6k

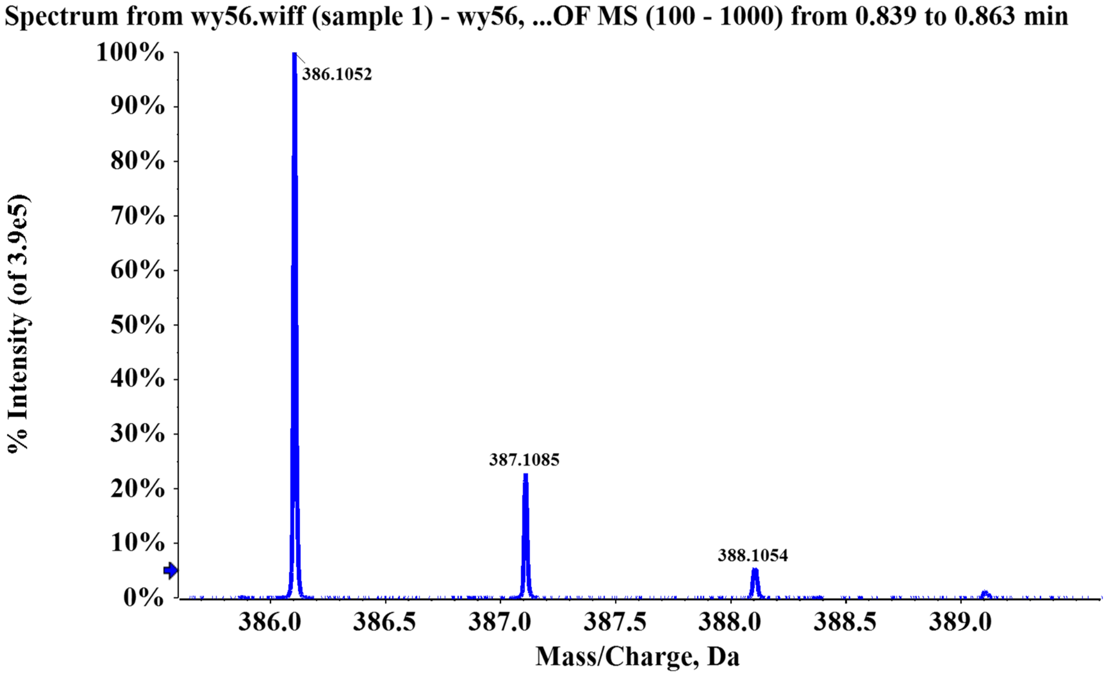

^{1}H NMR Spectrum of 6l (400 MHz, DMSO-d_6)

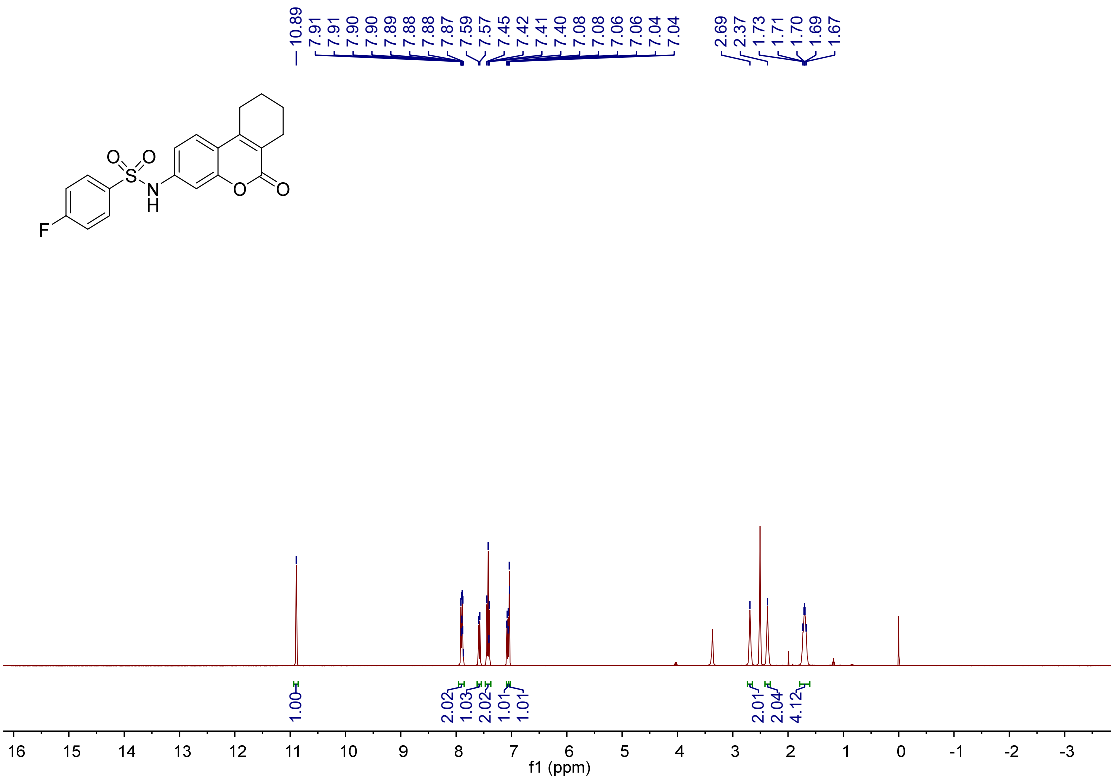

^{13}C NMR Spectrum of 6l (101 MHz, DMSO-d_6)

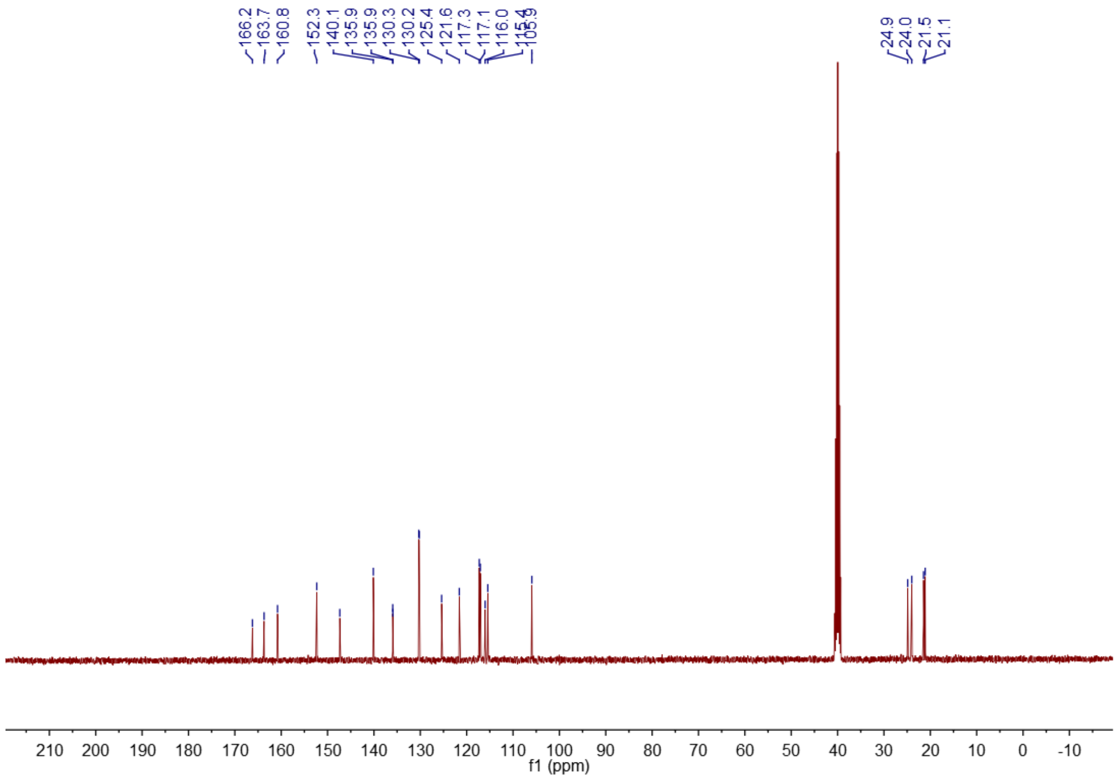

HRMS Spectrum of 6l

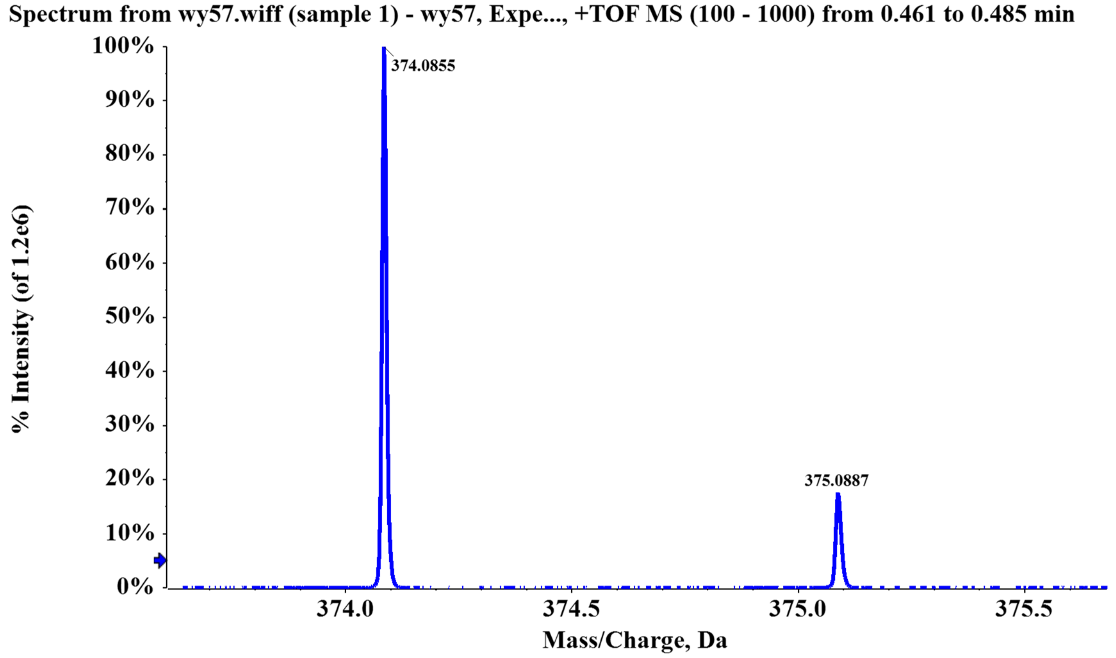

^{1}H NMR Spectrum of 6m (400 MHz, DMSO-d_6)

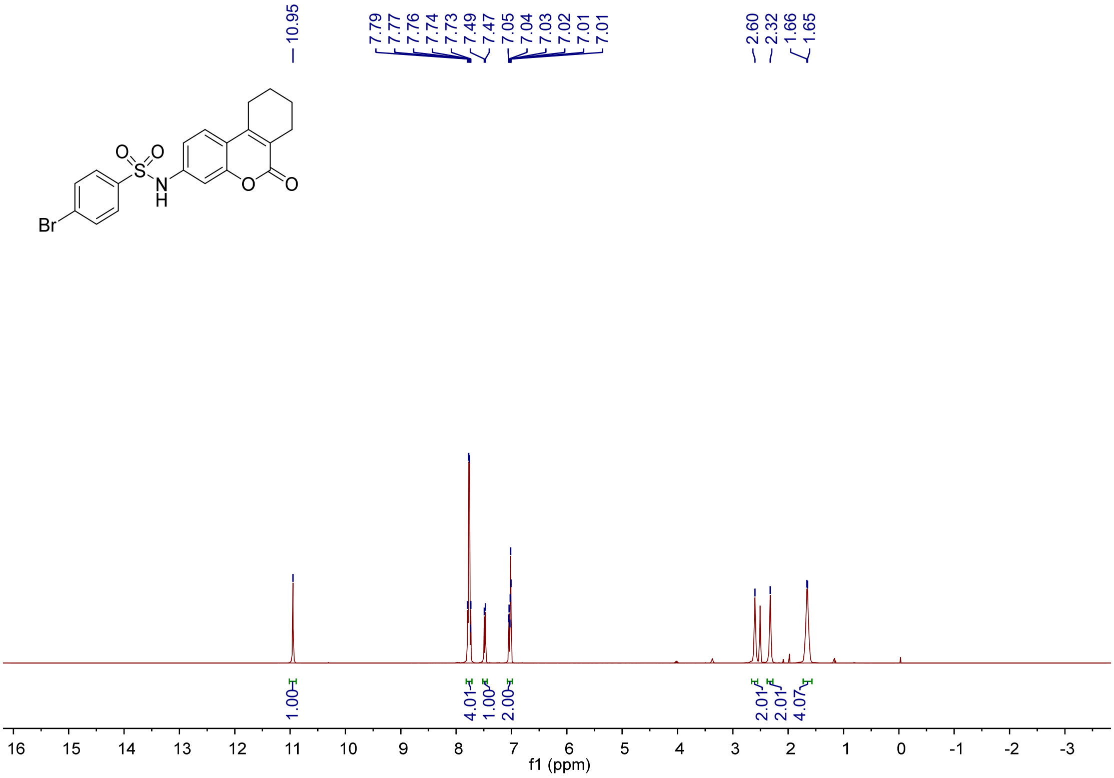

^{13}C NMR Spectrum of 6m (101 MHz, DMSO-d_6)

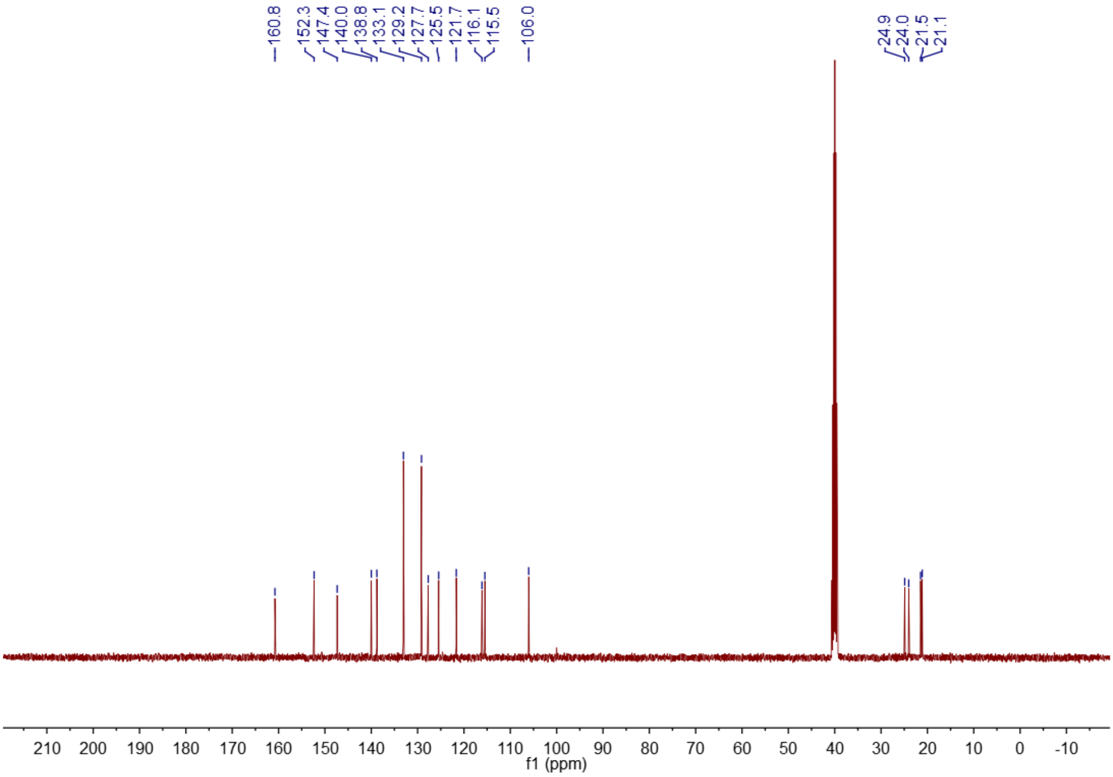

HRMS Spectrum of 6m

Spectrum from wy59.wiff (sample 1) - wy59, Ex... +TOF MS (100 - 1000) from 0.754 to 0.779 min

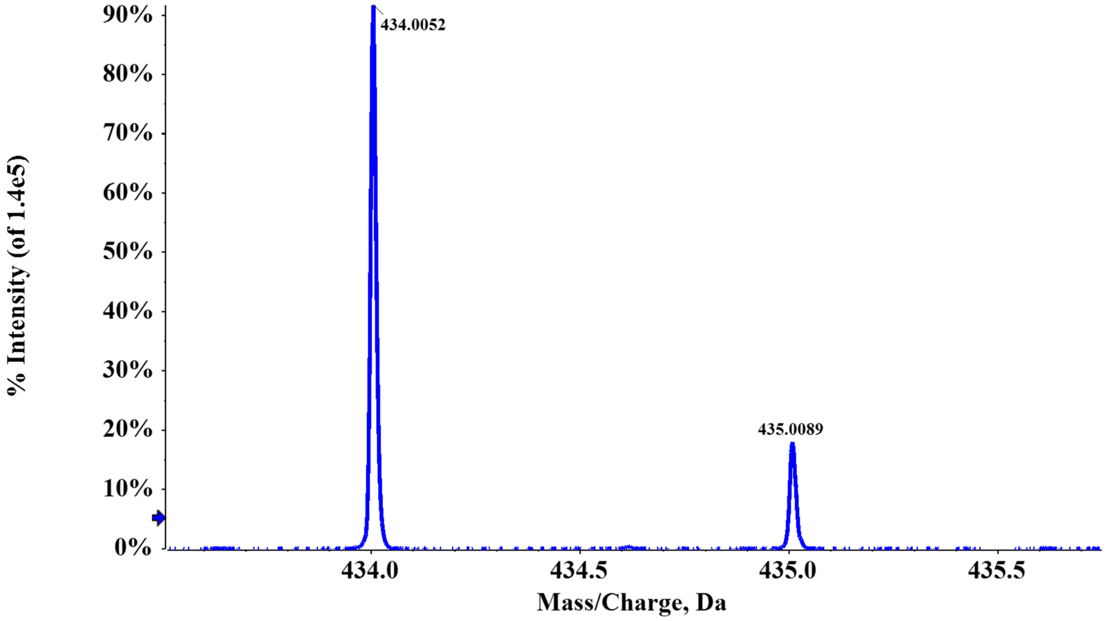

^{1}H NMR Spectrum of 6n (400 MHz, DMSO-d_6)

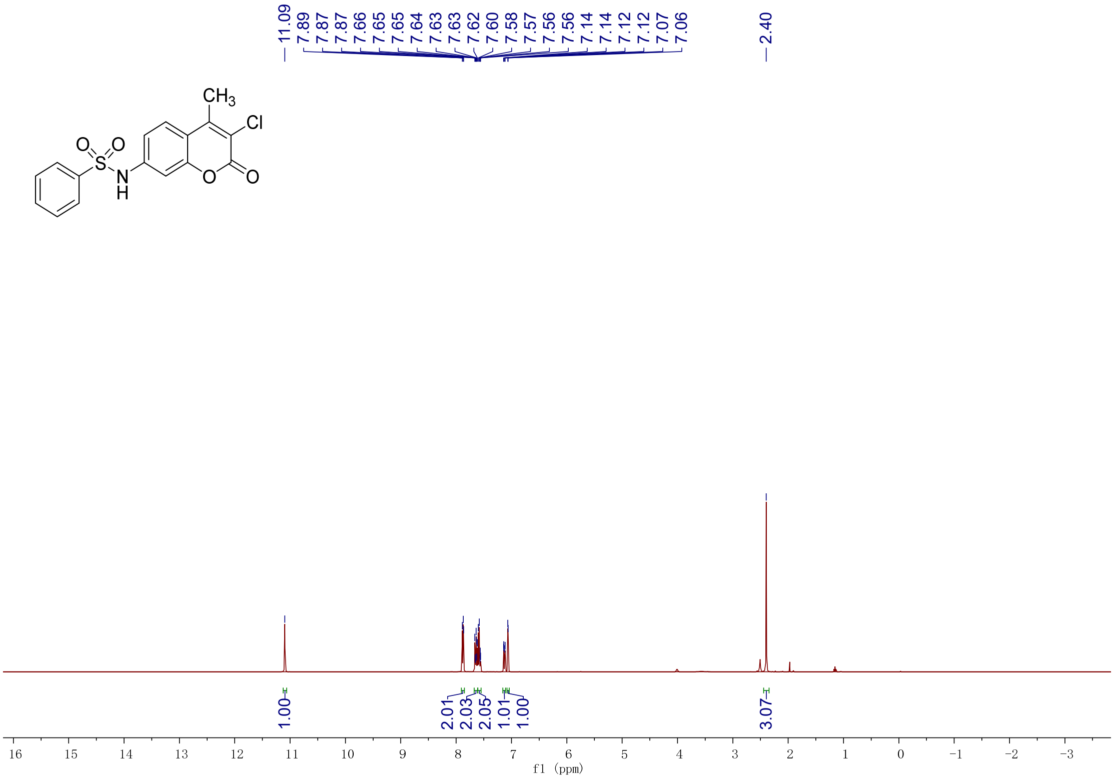

^{13}C NMR Spectrum of 6n (126 MHz, DMSO-d_6)

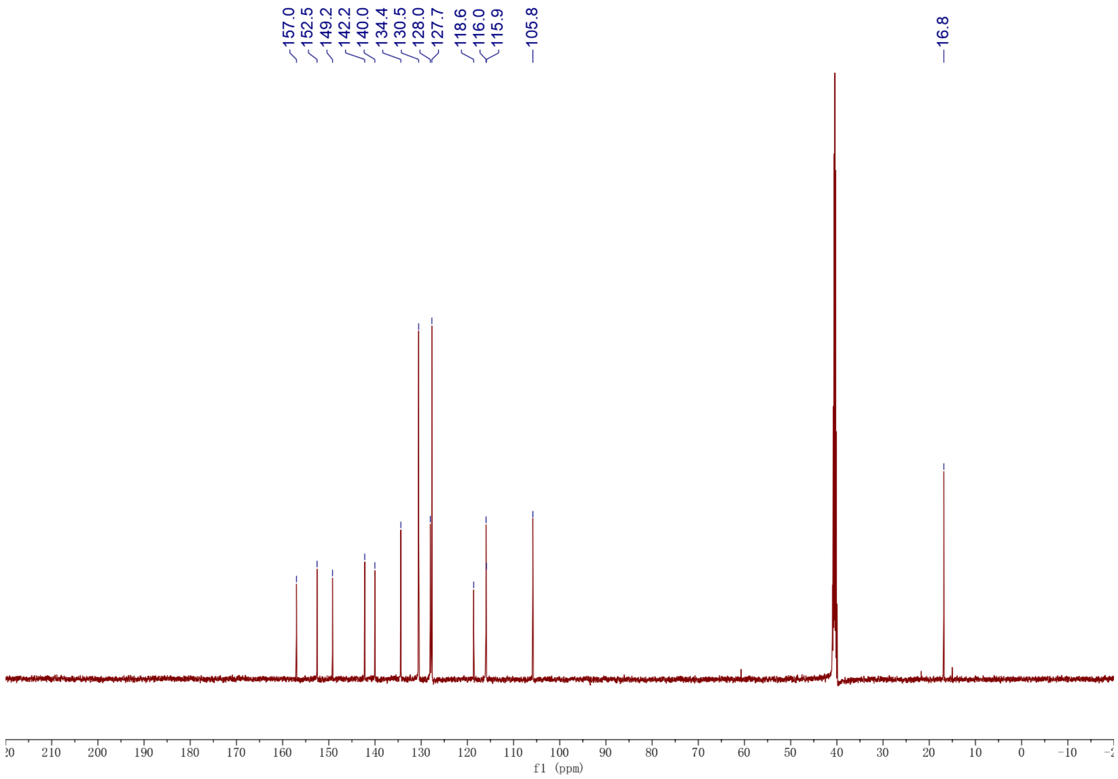

HRMS Spectrum of 6n

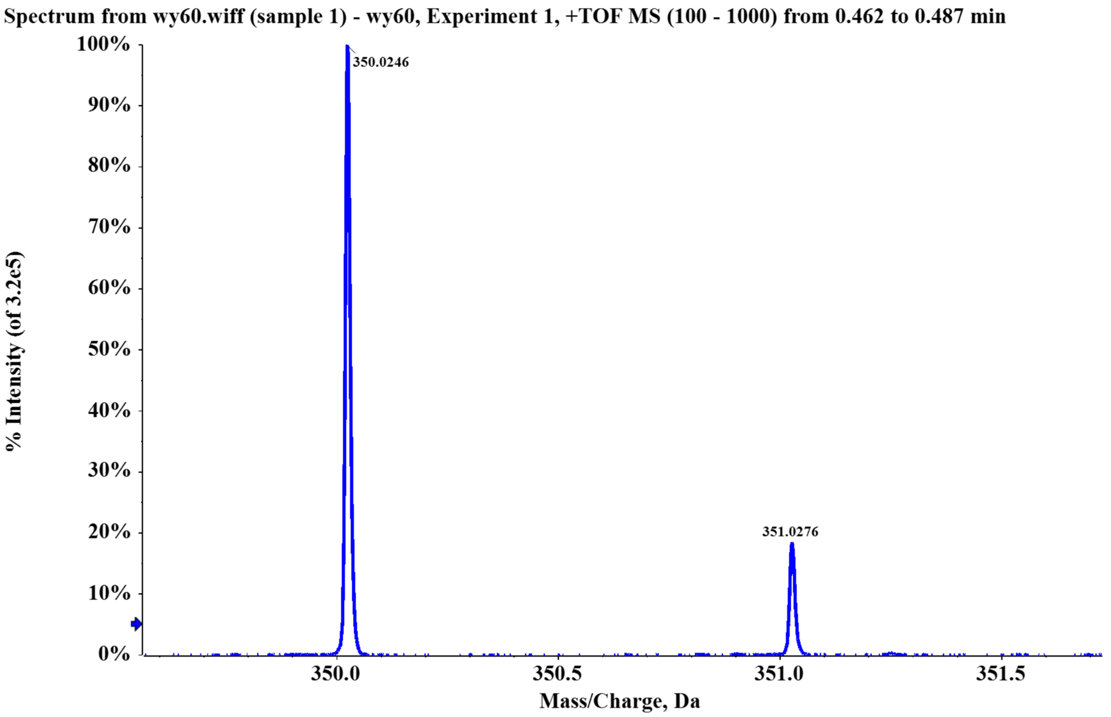

^{1}H NMR Spectrum of 6o (400 MHz, DMSO-d_6)

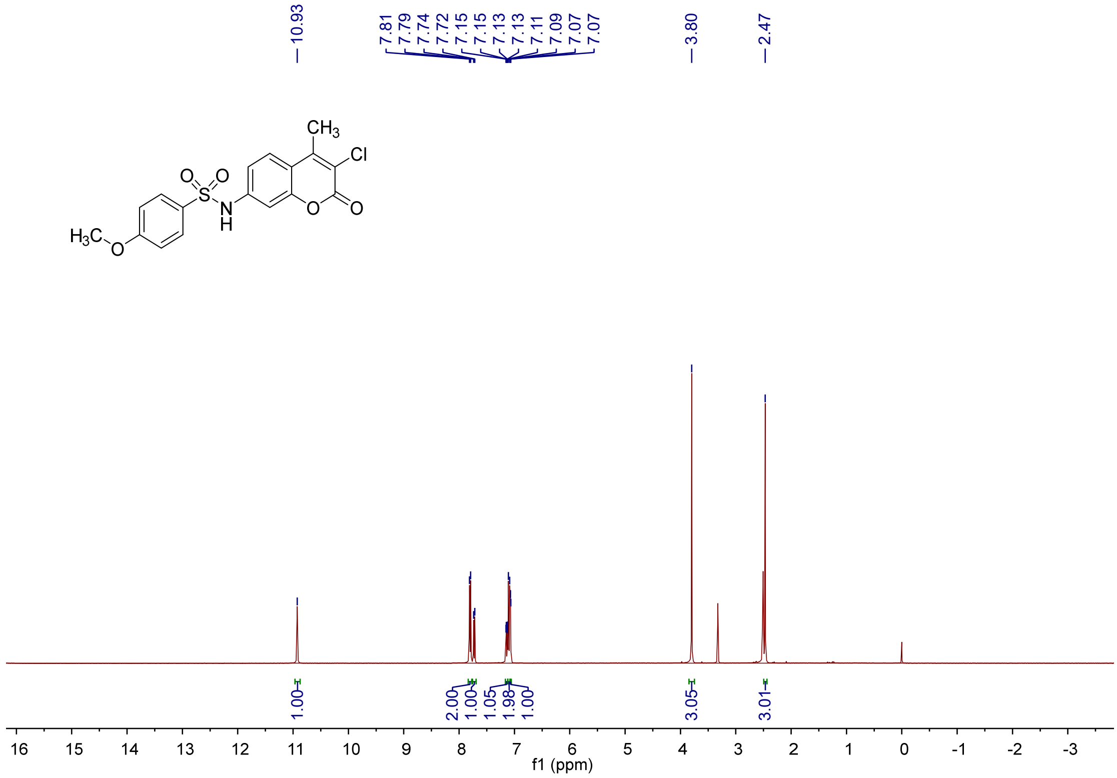

^{13}C NMR Spectrum of 6o (101 MHz, DMSO-d_6)

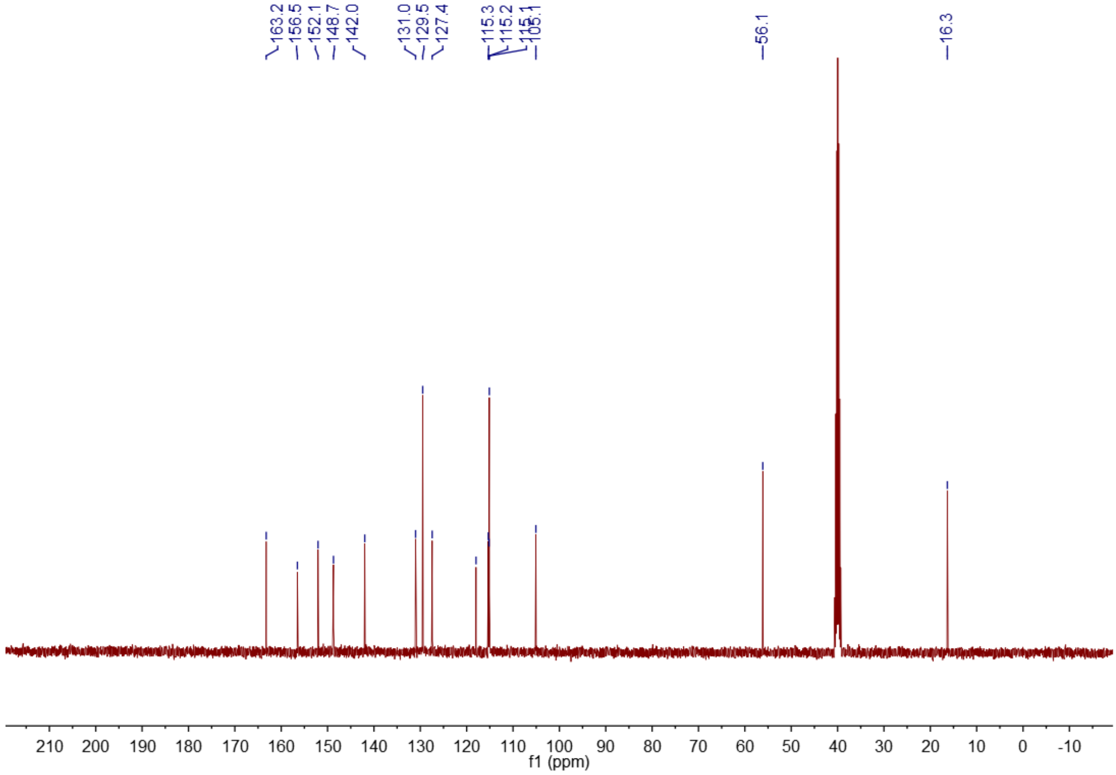

HRMS Spectrum of 6o

Spectrum from wy61.wiff (sample 1) - wy61... MS (100 - 1000) from 0.338 to 0.363 min

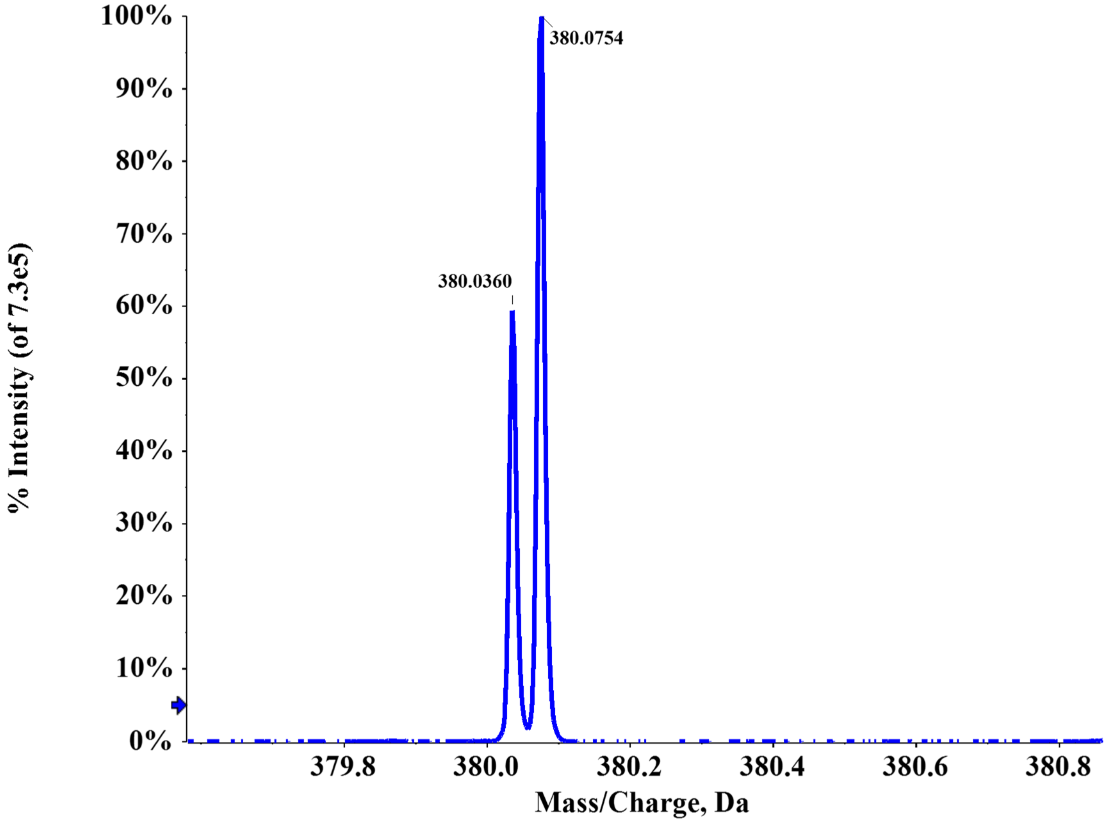

^{1}H NMR Spectrum of 6p (400 MHz, DMSO-d_6)

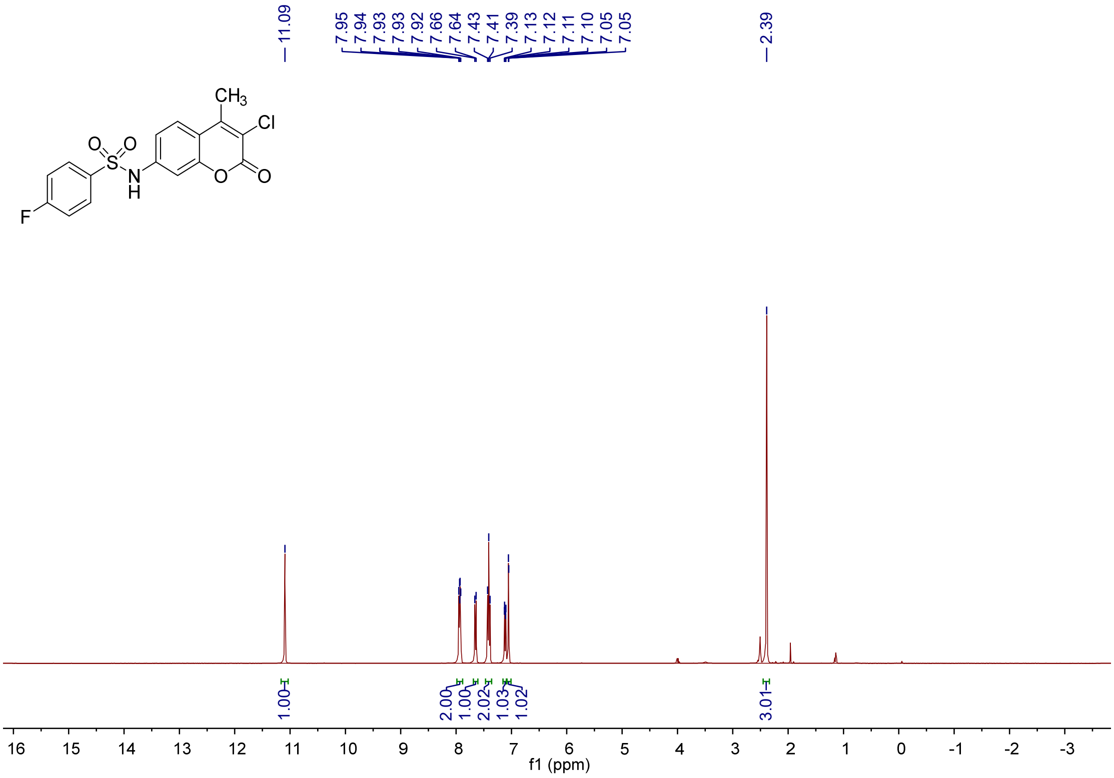

^{13}C NMR Spectrum of 6p (101 MHz, DMSO-d_6)

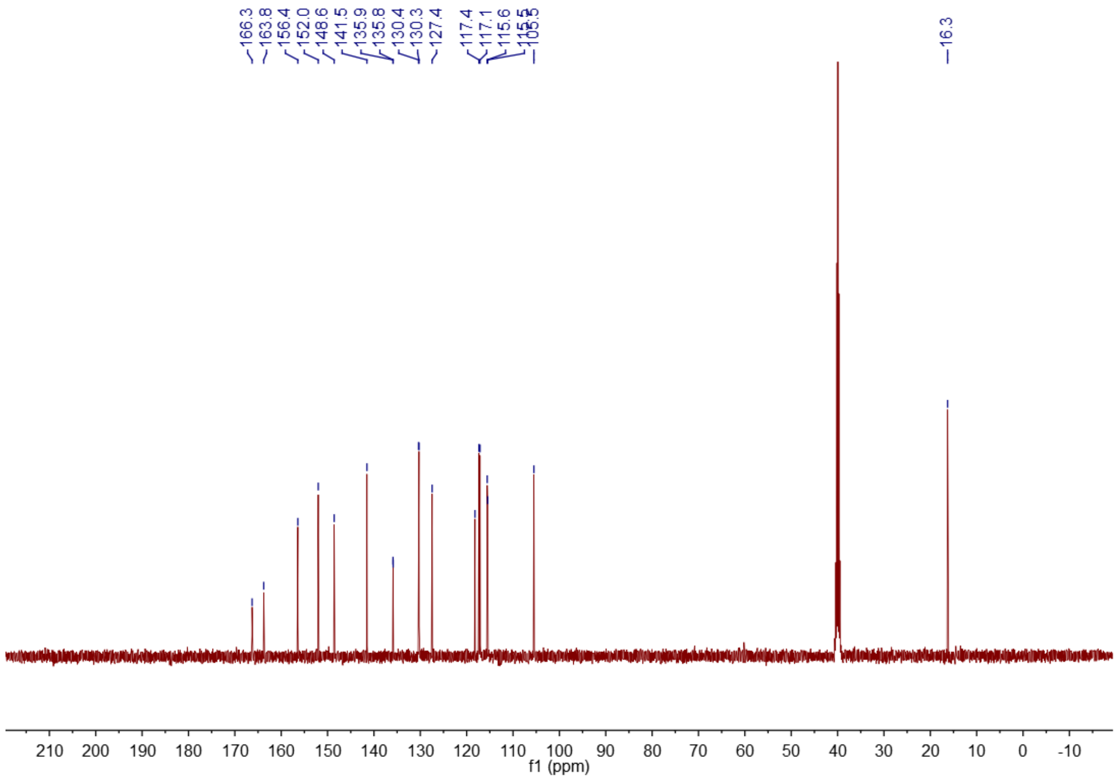

HRMS Spectrum of 6p

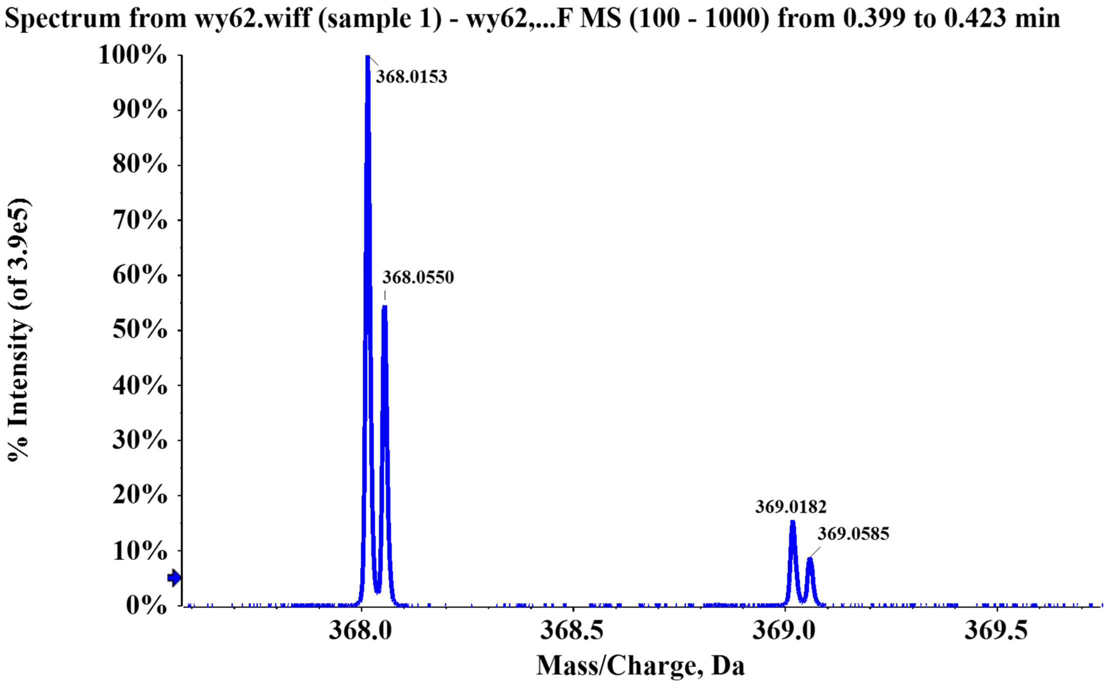

^{1}H NMR Spectrum of 6q (400 MHz, DMSO-d_6)

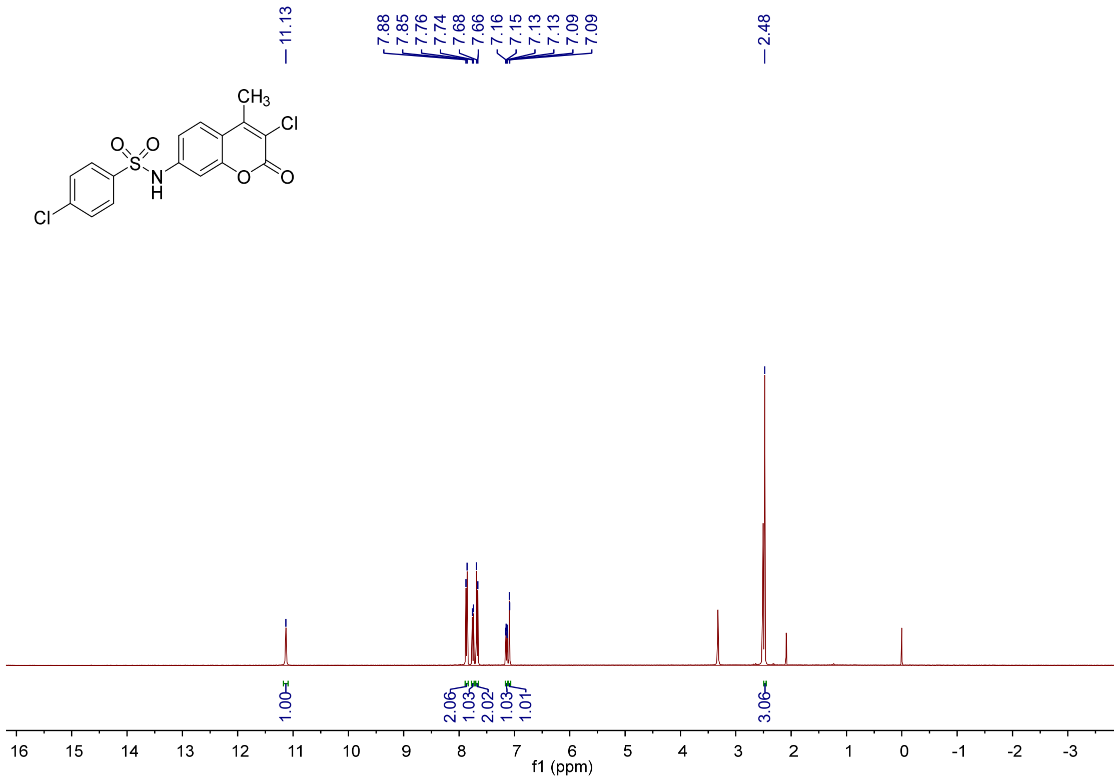

^{13}C NMR Spectrum of 6q (101 MHz, DMSO-d_6)

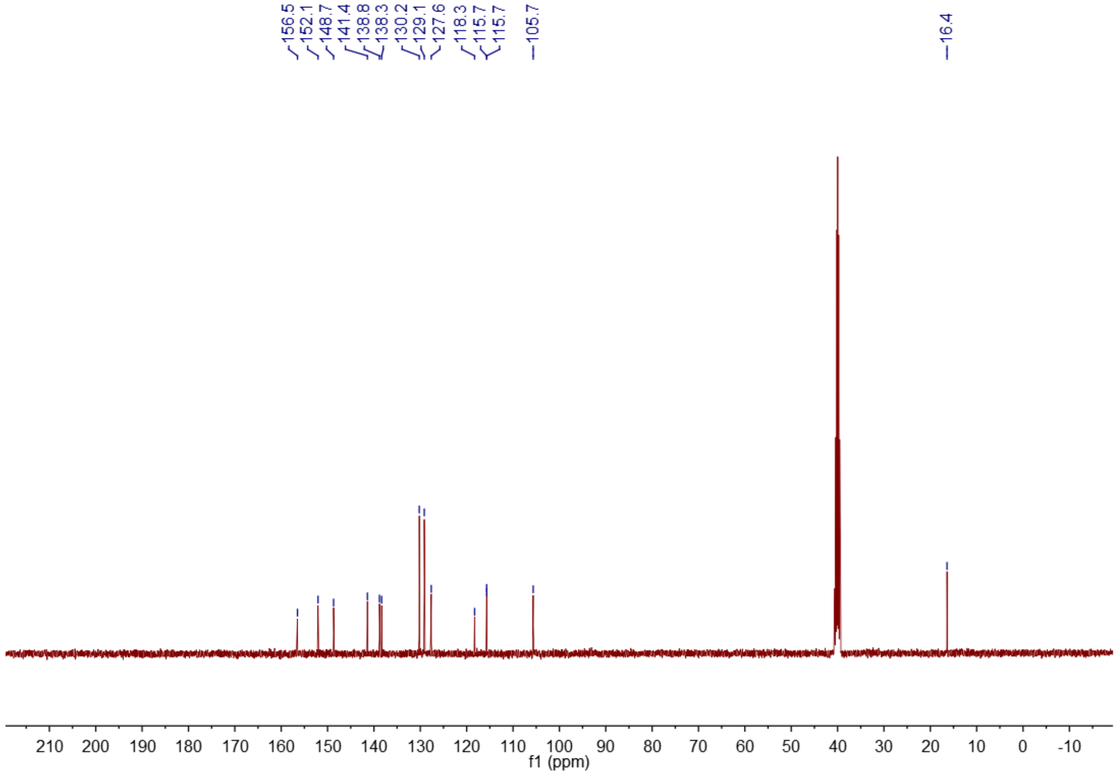

HRMS Spectrum of 6q

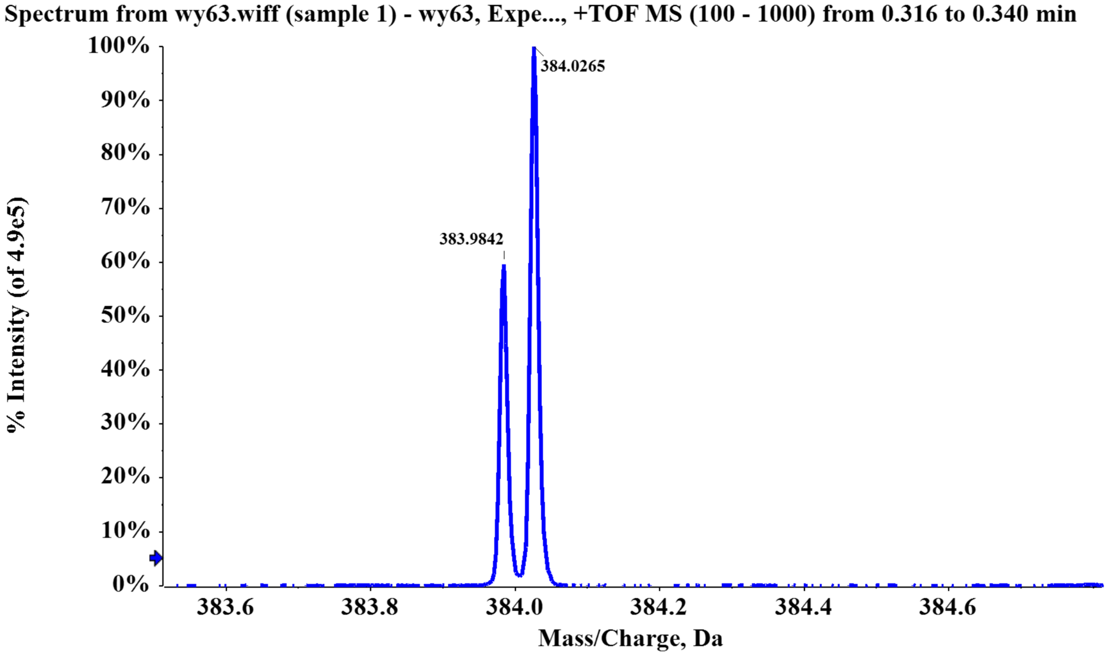

^{1}H NMR Spectrum of 6r (400 MHz, DMSO-d_6)

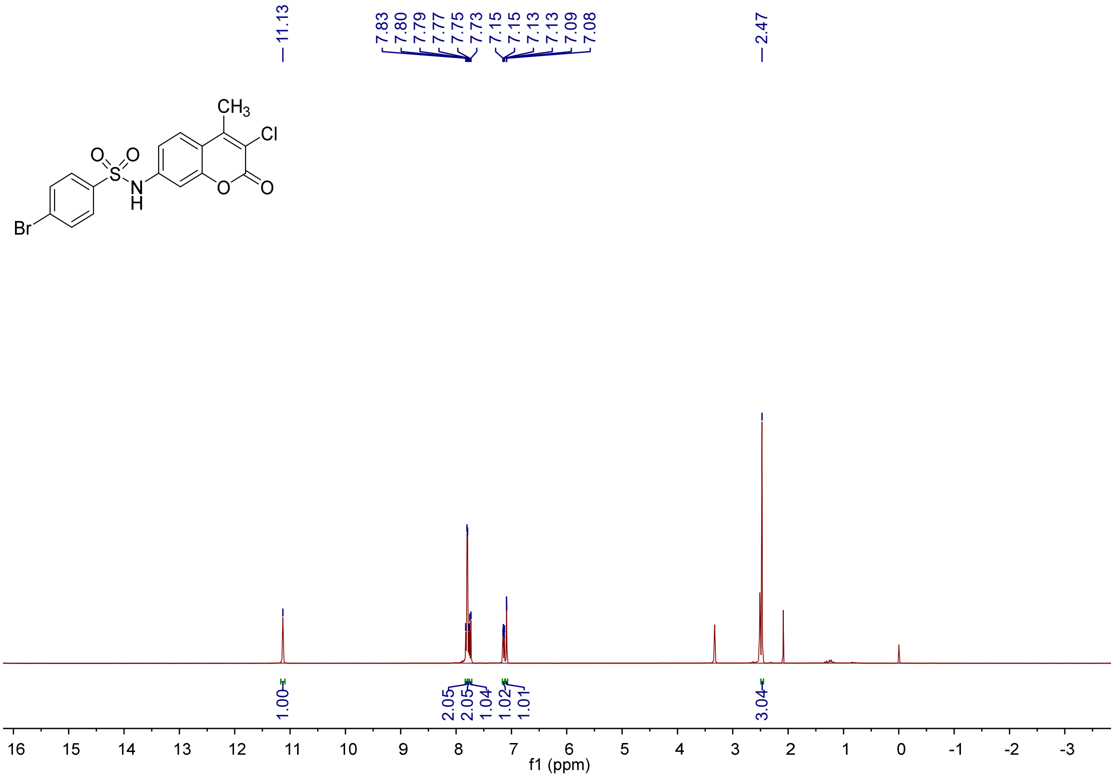

^{13}C NMR Spectrum of 6r (101 MHz, DMSO-d_6)

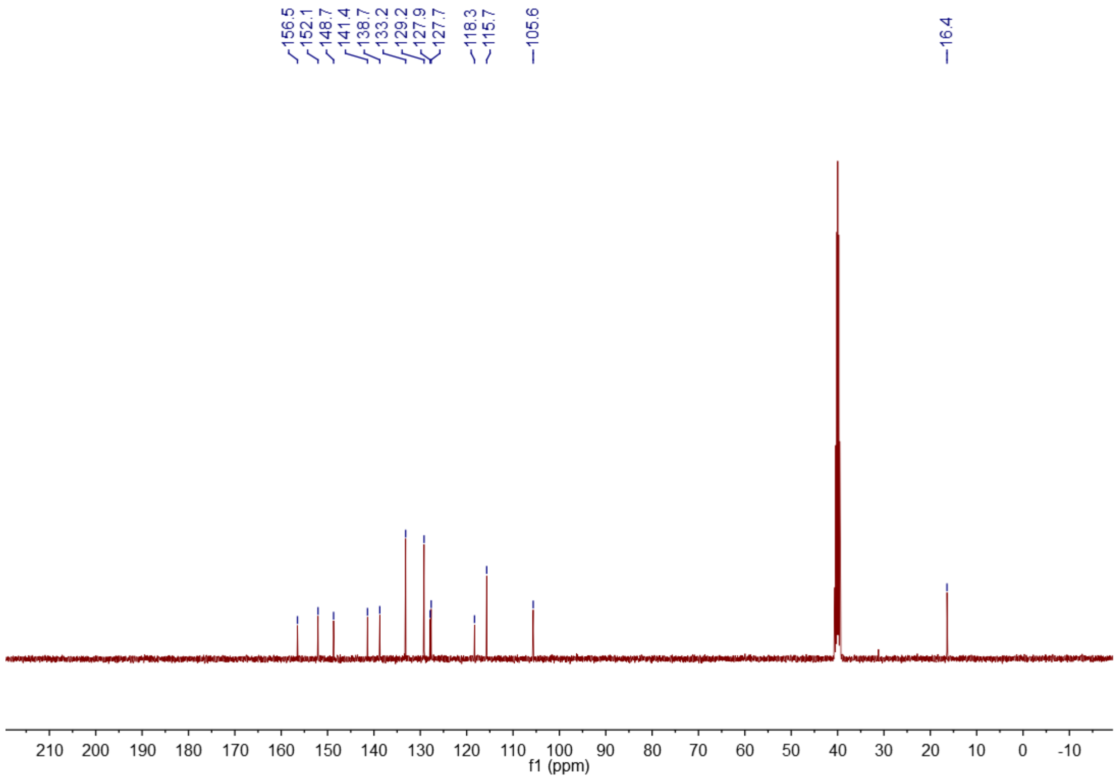

HRMS Spectrum of 6r

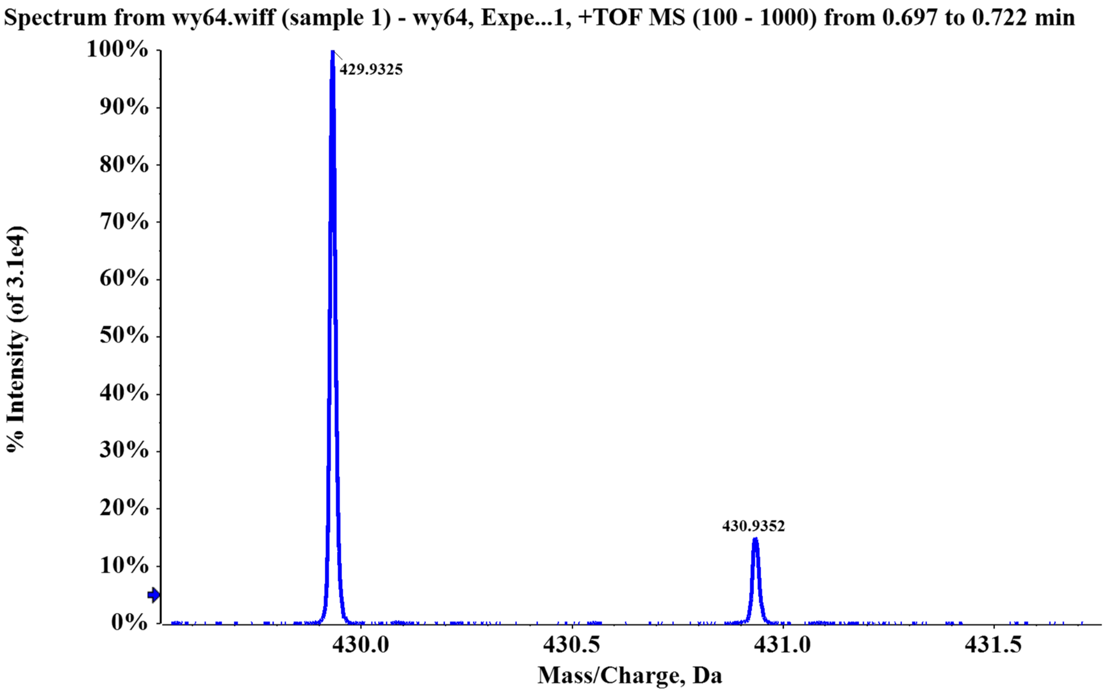

Supplement: Supplementary file 1 [file molecules-27-06904-s001.zip › molecules-1949730-supplementary.pdf]
